# ERK1 and ERK2 MAPK are key regulators of distinct gene sets in zebrafish embryogenesis

**DOI:** 10.1186/1471-2164-9-196

**Published:** 2008-04-28

**Authors:** SF Gabby Krens, Maximiliano Corredor-Adámez, Shuning He, B Ewa Snaar-Jagalska, Herman P Spaink

**Affiliations:** 1 Institute of Biology, Leiden University, Wassenaarseweg 64, 2333 AL Leiden, The Netherlands

## Abstract

**Background:**

The MAPK signaling proteins are involved in many eukaryotic cellular processes and signaling networks. However, specific functions of most of these proteins in vertebrate development remain elusive because of potential redundancies. For instance, the upstream activation pathways for ERK1 and ERK2 are highly similar, and also many of their known downstream targets are common. In contrast, mice and zebrafish studies indicate distinct roles for both ERKs in cellular proliferation, oncogenic transformation and development. A major bottleneck for further studies is that relatively little is known of i*n vivo *downstream signaling specific for these kinases.

**Results:**

Microarray based gene expression profiling of ERK1 and ERK2 knockdown zebrafish embryos at various stages of early embryogenesis resulted in specific gene expression signature sets that showed pronounced differences in gene ontology analyses. In order to predict functions of these genes, zebrafish specific *in silico *signaling pathways involved in early embryogenesis were constructed using the GenMAPP program. The obtained transcriptome signatures were analyzed in the BMP, FGF, Nodal and Wnt pathways. Predicted downstream effects of ERK1 and ERK2 knockdown treatments on key pathways responsible for mesendoderm development were confirmed by whole mount in situ hybridization experiments.

**Conclusion:**

The gene ontology analyses showed that ERK1 and ERK2 target common and distinct gene sets, confirming the difference in knockdown phenotypes and diverse roles for these kinases during embryogenesis. For ERK1 we identified specific genes involved in dorsal-ventral patterning and subsequent embryonic cell migration. For ERK2 we identified genes involved in cell-migration, mesendoderm differentiation and patterning.

The specific function of ERK2 in the initiation, maintenance and patterning of mesoderm and endoderm formation was biologically confirmed.

## Background

ERK1 and ERK2 (Extra-cellular signal Regulated protein Kinases) are most likely the best studied members of the mitogen activated protein kinase (MAPK) proteins. Despite much effort and their biological and medical importance, still relatively few *in vivo *downstream targets of these kinases have been identified conclusively, especially when considering the numerous cellular events and signaling networks they are involved in [1]. Most of the target proteins and downstream genes have been identified by *in vitro *studies using cell culture systems. Specific roles for both ERKs are described for cellular proliferation, as mouse embryos fibroblasts (MEF) isolated from *erk1*-/- mice grew faster than wild type cells. The tumorgenicity of transplanted NIH 3T3 cells stably expressing an oncogenic form of Ras in nude mice was largely inhibited by co-transfection of ERK1, but not by ERK2 or p38 [2]. In diseases, ERK1 and ERK2 can display distinct cellular functions, as has been shown for the formation of cancer [3]. The upstream activators MEK1 and MEK2 have also been shown to play a role in human diseases such as Cardio-Facio-Cutaneous (CFC) syndrome [4]. In addition, divergent roles for ERK1 and ERK2 were already shown by the different effect of the knockout studies performed in mice since *erk1*-/- mice are viable and fertile [5], while *erk2*-/- mice die *in utero *before embryonic day (E) 8.5 [6].

To study and compare the developmental roles of ERK1 and ERK2 we used specific morpholino antisense oligonucleotides (MO), to block translation of ERK1 and ERK2. We previously showed that saturated knockdown conditions of ERK2 led to severe phenotype, as ERK2MO morphants did not go into epiboly, whereas ERK1MO morphants still developed further and entered gastrulation stages. In addition, immuno-histochemical studies showed that ERK phosphorylation was completely abolished in the blastula margin of ERK2 morphants, indicating that ERK2 is the active ERK MAPK in the margin and essential for epiboly initiation and further progression of the developmental program (Krens et al., manuscript in preparation). Possibly ERK2 also functions in mesendodermal differentiation processes in the blastula margin, as FGF is known to activate the canonical MAPK pathway in a Ras dependent manner (reviewed by Gotoh and Bottcher [7,8]). The severe phenotype of ERK2 morphants indicate that ERK2 has a more dominant role than ERK1 during early developmental processes, as also suggested by the mice knockout phenotypes.

Here we aim to further determine specific downstream gene targets of ERK1 and ERK2 during vertebrate development, by performing expression profiling analysis using a microarray approach. We compared the expression profiles of ERK1 and ERK2 knockdown embryos, using specific morpholino antisense oligonucleotides (MO), which specifically block the translation of a gene of interest into a functional protein [9]. Recently developed software programs and web-based analysis tools, e.g. Rosetta Resolver, GenMAPP and GeneTOOLS eGOn were used for the processing and comparisons of large expression datasets and biological interpretation of the data and to facilitate the prediction of interconnections between developmental signaling pathways that were tested by biological assays (qPCR and in situ hybridizations).

Analysis of the obtained data revealed that ERK1MO and ERK2MO knockdown affect signature sets of common target genes, as well as signature sets of specific genes. Surprisingly, we also identified gene sets in which the expression patterns were anti-correlated. Several signature marker genes identified in this study were confirmed by quantitative real time PCR and in situ hybridization. We performed signaling pathway analysis on the obtained ERK1 and ERK2 transcriptome signatures, using the GenMAPP software program [10,11] for the analysis of important signaling cascades during early vertebrate development. These include BMP, FGF, Nodal and Wnt signaling pathways [12]. For ERK1 knockdown we identified a connection with genes involved in dorsal-ventral patterning and subsequent embryonic cell migration. For ERK2 knockdown we identified a connection with genes involved in mesoderm and endoderm initiation, differentiation and patterning. Many of these genes also play a role in morphogenic cell migration processes during later stages of development. The outcomes of the predictions for ERK2 knockdown on developmental signaling were confirmed by in situ hybridization experiments indicating that ERK2 controls mesoderm and endoderm initiation, maintenance and patterning.

## Results

### Distinct gene expression signature sets of ERK1 and ERK2 knockdown embryos

**Figure 1:**
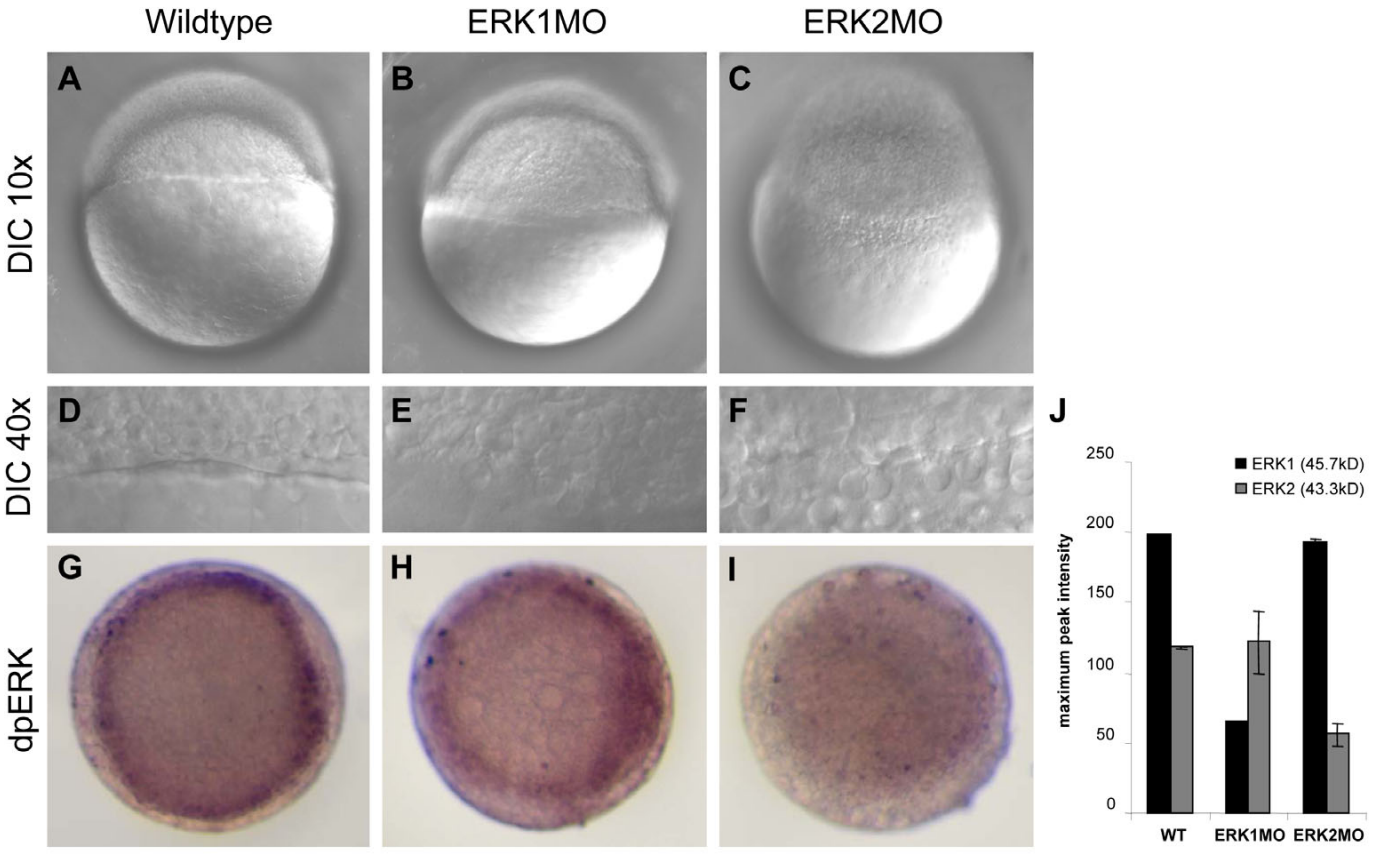
**Phenotype and Function analysis of morpholino mediated knockdown of ERK1 and ERK2**. Differential interference contrast (DIC) microscopy of 4.5 h old embryos using a 10× objective (A,B,C,) or an enlargement of the margin, using a 40× objective (D,E,F) Wild type (Wt) and ERK1 morphants are at approximately 30% epiboly stage and undergo epiboly, whereas ERK2 morphants do not initiate epiboly. Localization of active ERK (dpERK) was detected by immuno-localization in wild type, ERK1MO and ERK2MO injected embryos at 4.5 hpf (G-I) by phospho-specific ERK antibody. The level of dpERK was lower in ERK1 morphants compared to wild type embryos, whereas ERK2 morphants hardly showed any active ERK staining, (A-C); lateral view, animal pole to top, (G-I); animal pole view, dorsal to right. The bar graphs in (J) represent the quantification of a western blot analysis of zebrafish wild type, ERK1MO and ERK2MO injected embryos, probes with a global ERK1 antibody (Santacruz). This antibody recognizes both zebrafish ERK1 (45.7 kD) and ERK2 (43.3 kD) protein. The bars represent the maximum pixel-intensity measured in duplo and clearly show the specific knockdown of either ERK1 or ERK2 by the corresponding morpholino.

A morpholino knockdown approach was used to block translation of either ERK1 or ERK2 by injection of 0.4 mM (= 3.4 ng/embryo) morpholinos (MO) targeting ERK1 (ERK1MO) or ERK2 (ERK2MO). The knockdown embryos, also referred to as morphants, showed severe phenotypes after depletion of ERK2. These embryos did not enter epiboly at 4.5 hpf and the blastula cells remained on top of the yolk, preventing further development of the embryo (Fig. 1C). In addition, ERK2 knockdown induces disorganization of the margin. (Fig. 1F). Wild type embryos reached 30% epiboly at this time (Fig. 1A,D) [13]. In contrast, ERK1 morphants did not show any obvious phenotypes at this point yet and had entered epiboly (Fig. 1B,E). However, ERK1 morphants did show strong phenotypes at later stages in embryogenesis (manuscript in preparation and additional file 2).

The specificity of ERK1 and ERK2 knockdown phenotypes was rescued by co-injection of synthetic mRNA (data not shown, manuscript in preparation), western blot analysis (Fig. 1J) and immuno-localization in wildtype, ERK1MO and ERK2MO injected embryos at 4.5 hpf (Fig. 1G–I) using a phospho-specific ERK antibody (dpERK). ERK1 morphants (Fig. 1H) show similar levels of dpERK staining at the dorsal margin compared to wild type embryos (Fig. 1G), but the dpERK signal in ERK1 morphants is reduced at the ventral half of the margin. ERK2MO injected embryos hardly show any dpERK staining and the active ERK signal is depleted from the marginal ring in these embryos (Fig. 1I). Quantification of a western blot analysis of zebrafish wild type, ERK1MO and ERK2MO injected embryos, probes with a global ERK antibody (Santa Cruz Biotechnology), recognizes both zebrafish ERK1 (45.7 kD) and ERK2 (43.3 kD) protein clearly shows the specific knockdown of either ERK1 or ERK2 by the corresponding morpholino (Fig. 1J).

Addition of different MEK specific inhibitors (U0126 or PD98059, Cell Signaling technologies), did not result in the same phenotypes as obtained by the ERK2MO mediated knockdown. The inhibiting effects of these drugs were confirmed by Western blot analysis, but apparently these effects were not efficient enough to block epiboly (data not shown). Because it is not possible to address the specific functions of either ERK1 or ERK2 using these chemical inhibitors, we did not proceed with these experiments.

These data prove the functionality of the morpholino-mediated knockdown of either ERK1 or ERK2. In addition, the severe phenotype of ERK2 morphants indicate defects in crucial early developmental processes and most likely affects the expression levels of a larger number of genes than knockdown of ERK1.

### Distinct ERK-knockdown gene expression profiles in time

**Figure 2:**
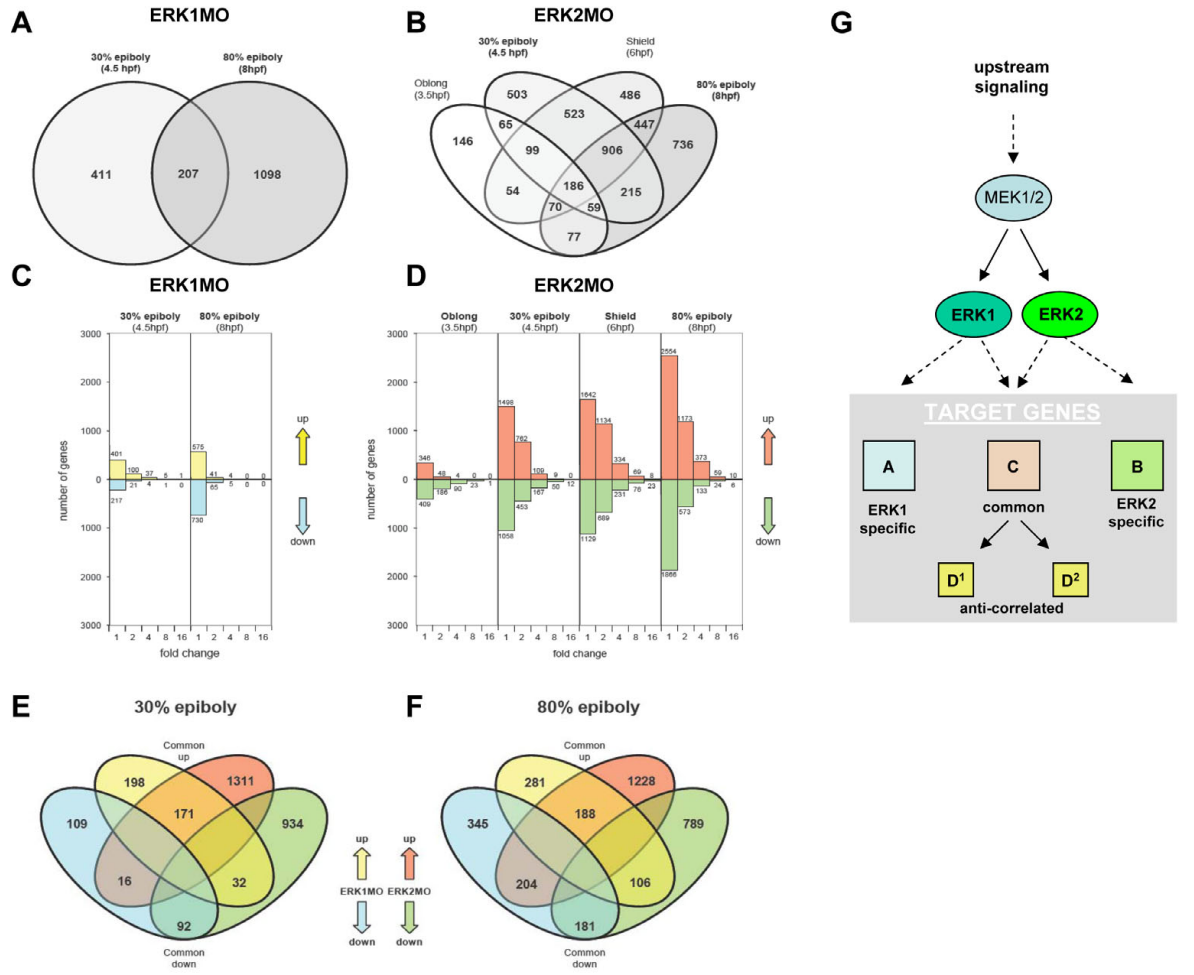
**Comparison of the ERK1 and ERK2 knockdown gene expression profiles in time**. (A,B) Venn diagrams showing the gene expression profiles in time for ERK1 and ERK2 knockdown respectively. (C,D): graphs representing the number of genes that showed changes in expression, as well as their fold of change (greater than 1, 2, 4, 8, and 16 fold changes) upon knockdown of ERK1 (C; 4.5 and 8hpf) or ERK2 (D; 3.5, 4.5, 6 and 8hpf) at (p < 10^-5^). Knockdown of ERK2 affects the expression of more genes, and with a higher fold of changes than knockdown of ERK1, but increased in time for both conditions. (E,F); Venn diagrams, comparing ERK1 versus ERK2 expression profiles at 30% epiboly and 80% epiboly respectively. The signatures of ERK1 and ERK2 morphants are split in up- and down-regulated genes in the graphs (C,D) and Venn diagrams (E,F). By doing so, the Venn diagrams also shows the numbers of specifically up and down regulated genes, common up and down regulated genes, and two anti-correlated gene pools (up-regulated in ERK1MO down-regulated in ERK2MO and down-regulated in ERK1MO up-regulated in ERK2MO); yellow = up-regulated by ERK1MO (ratio > 1), blue = down-regulated by ERK1MO (ratio <1), red = up-regulated by ERK2MO (ratio > 1), green = down-regulated by ERK2MO (ratio <1). (G) A model for the downstream ERK1 and ERK2 signaling pathway, showing distinct functions for ERK1 and ERK2 in gene regulation. Panels (A) represents ERK1 specific genes, (B) ERK2 specific genes, (C) common genes, and D^1 ^and D^2^, are representing two different anti-correlated gene pools as sub-populations of the common gene pool C.

To identify specific gene pools affected by the knockdown of ERK1 or ERK2, and to identify possible downstream targets, microarray based transcriptome analysis was performed using Agilent zebrafish microarrays. As a control for aspecific morpholino effects, a standard control morpholino (GeneTools Philomath, OR, USA) was injected in the same concentration. This did not result in any phenotypes during zebrafish development. The RNA from these standard control MO injected embryos was used as a reference to compare the transcriptomes of both ERK1MO and ERK2MO injected embryos. We annotated the Agilent 22K-zebrafish microarray chip by BLAST searches with all oligonucleotide sequences in the zebrafish genome. From the complete number of 21495 oligonucleotides from the Agilent 22K zebrafish chip, 16675 oligonucleotides were assigned a Unigene ID (build #105). The phenotypic effect of ERK2 depletion was observed at 30% epiboly indicating an altered gene expression profile at earlier stages. Therefore we analyzed the gene-expression profile of ERK2 morphants at more time points than ERK1 morphants (Fig. 2A,B). We obtained gene expression profiles for ERK1 morphants at 4.5 hpf and 8 hpf, and for ERK2 morphants at 3.5 hpf, 4.5 hpf, 6 hpf and 8 hpf (equivalent to oblong-stage, 30% epiboly, shield-stage and 80% epiboly time points), as shown in figure 2C and 2D.

Comparison of the gene expression profiles of ERK1 and ERK2 morphants at various stages showed a larger number of Unigene identifiers with significant changes (p < 10^-5^) in ERK2 compare to ERK1 morphants in time, as illustrated in a Venn-diagram (Fig. 2A and 2B). These Venn diagrams also show that 207 genes are affected in expression at both 30% and 80% epiboly in ERK1 morphants, whereas in the ERK2 morphants time-series, we find 186 genes to be significantly changed in expression in all time points. At 30% and 80% epiboly the numbers of genes with an altered expression was larger and with a higher fold of change for ERK2 than for ERK1 morphants (Fig. 2C and 2D). This is in agreement with the phenotype of ERK2 knockdown embryos that indicates a more prominent role for ERK2 in early development (Fig. 1). The effect of ERK1 knockdown becomes more pronounced at 80% epiboly (Fig. 2A,C). This indicates that ERK1 may become relatively more important at later developmental stages.

Comparing the effect of ERK1 and ERK2 knockdown, we found distinct gene expression signature sets during embryonic development. (Fig. 2E,F and 2G). In addition to commonly affected genes (Fig. 2G, target gene pool C), we found distinct genes that were specifically regulated by either knockdown of ERK1 (Fig. 2G, target gene pool A); 198 vs. 281 up-regulated, 109 vs. 345 down-regulated at 30 or 80% epiboly respectively) or knockdown of ERK2 (Fig. 2G, target gene pool B); 1311 vs. 1228 up-regulated, 934 vs. 786 down-regulated), or genes which were regulated in an anti-correlated manner: 32 genes (30% epiboly) or 106 genes (8 hpf, equivalent to 80% epiboly time point) were up-regulated by knockdown of ERK1 whereas they were down-regulated by knockdown of ERK2 (Fig. 2G, target gene pool D^1^; anti-correlated gene-pool 1) and 16 genes (30% epiboly) or 204 genes (8 hpf, equivalent to 80% epiboly time point) were down-regulated by knockdown of ERK1 whereas they were up-regulated by knockdown of ERK2 (Fig. 2G, target gene pool D^2^; anti-correlated gene-pool 2). These results confirm that ERK1 and ERK2 MAPK are key regulators of distinct gene signature sets during embryonic development. This is supported even when comparing multiple gene expression profiles from different developmental time-points (Fig. 2).

**Figure 3:**
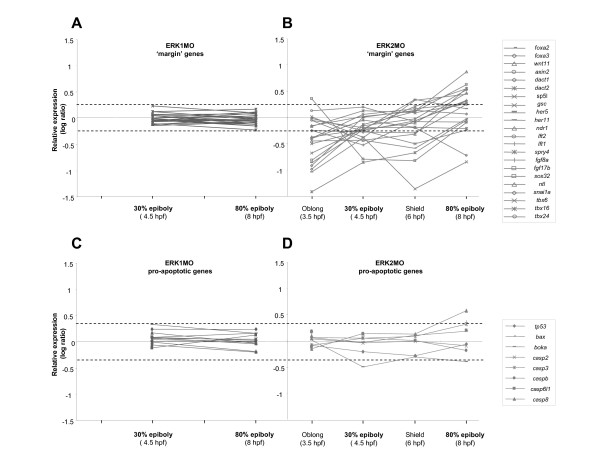
**Trend lines of the expression levels of 'margin genes' in ERK1 and ERK2 morphants, during early developmental stages**. (A,B) The relative expression (log ratio) of a number of a number of genes, selected for their described expression in the margin at the onset of epiboly, is plotted over different developmental stages in ERK1 and ERK2 morphants. The expression levels of the selected genes hardly changes in time in ERK1 morphants, whereas in ERK2 morphants the trend of the selected genes reveals a possible showed stabilization in their expression and possible subsequent recovery in time. Dotted line indicates the limits of the expression levels for the selected margin-genes in ERK1 morphants. (C,D) Trend lines for genes involved in apoptosis. Only in ERK2 morphants an increase in apoptotic genes (*casp3 *and *casp8*) was observed. Overlapping time-points are indicated in bold (30% and 80% epiboly).

Because we observed a strong activated ERK signal in the margin at the onset of epiboly, we compared the expression levels of a selection of genes that are described to be expressed in the margin at the onset of epiboly in time. To do so, a gene-expression trend-line of the selected margin genes for ERK1 and ERK2 morphants was constructed (Fig. 3A,B). Most of the selected genes did not give a significant difference in time upon ERK1 knockdown, suggesting different developmental functions for ERK1, whereas in ERK2 morphants the expression levels of most of the selected 'margin'-genes was affected. A common trend in the expression-levels of the selected genes was observed upon ERK2 depletion, as most genes showed stabilization in their expression levels between 30% epiboly and shield stage, and even recovery between 6 to 8 hpf. Despite this, the presumptive blastula cells remained on top of ERK2 morphants. This indicates that the obtained gene expression profiles of later stages of ERK2 morphants (6hpf and 8hpf) are the results of a prolonged epiboly arrest, most likely due to multiple secondary developmental defects (Fig. 2 and 3). To analyze possible apoptotic effects in the ERK2 morphants, we also made similar trend lines with a selection of genes that are associated with apoptotic responses (Fig. 3C,D). The apoptotic responses by the ERK1MO treatment are minimal and also ERK2MO injected embryos do not show obvious responses in the earlier stages (3.5–6hpf). However, the apoptosis responsive genes *casp8 *and *casp3 *revealed an increased expression at 8hpf in ERK2 morphants. These combined results were the rational for limiting the further comparisons of the effects of ERK1 and ERK2 knockdown at 30% epiboly.

The identified gene-sets of correlated and anti-correlated regulated genes by knockdown of either ERK1 or ERK2 at 30% epiboly are listed and annotated [see Additional file 1, tables 1 and 4]. To identify the ERK1MO and ERK2MO specific genes, we focused on the genes that were most significantly affected. Therefore we used the following criteria: the absolute fold change must be at least 1.5 in each independent replicate and the common p-value provided by the error-model taking into account all hybridizations must be smaller than 10^-5^. The genes that were only found in either ERK1MO or ERK2MO gene-pools were manually annotated and assigned gene designations [see Additional file 1, tables 5 and 6].

### Quantitative real time PCR analyses confirm the different ERK1- and ERK2- knockdown gene expression profiles

**Figure 4:**
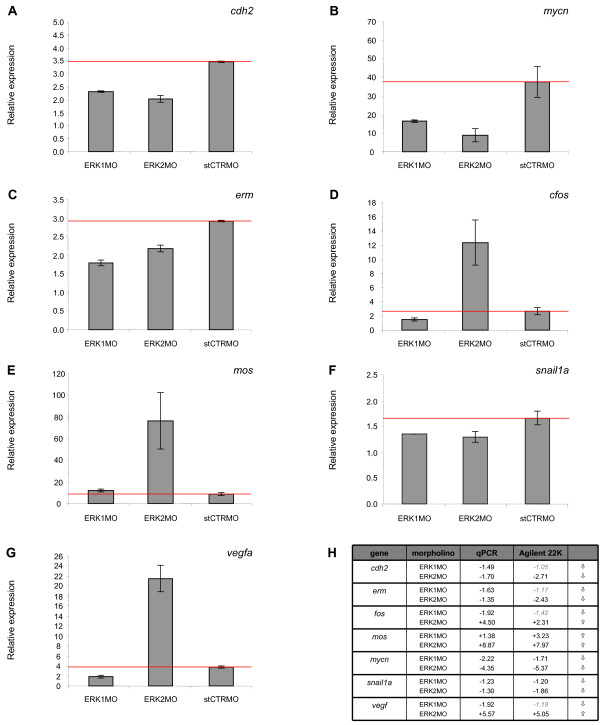
**Quantitative real-time PCR confirmation of the microarray results**. (A-G) qPCR was performed on seven genes that showed differential regulation of expression in response to knockdown of either ERK1, ERK2 and the standard control MO control:*cdh2 *(NM_131081), *mycn *(NM_212614), *erm *(NM_131205), *cfos *(NM_205569), *mos *(NM_205580), *snai1a *(NM_131066) and *vegf *(NM_131408), correlated to the β-actin housekeeping gene. A comparison of the fold changes in expression of these genes, detected by qPRC assay and microarray, are listed in a table (H). ⇧ = induction of expression, ⇩ = repression of expression, compared to the standard control MO (A-C, red line).

To confirm the results of the microarrays experiments, quantitative reverse transcriptase PCR analysis was performed on seven regulated genes at 4.5 hpf (30% epiboly) that were chosen as hallmarks of the differences between the ERK1 and ERK2 morphant expression profiles. The expression levels were tested on the same RNA samples as used for the microarray analysis for *cdh2 *(cadherin 2, neuronal, NM_131081), *mycn *(v-myc, myelocytomatosis viral related oncogene, neuroblastoma derived, NM_212614), *erm *(ets related protein erm, NM_131205), *cfos *(FBJ murine osteosarcoma viral oncogene homolog, NM_205569), *mos *(moloney murine sarcoma viral oncogene homolog, NM_205580), *snai1a *(snail homolog 1a *Drosophila*, NM_131066) and *vegf *(vascular endothelial growth factor A, NM_131408) (Fig. 4). β-actin was taken as reference to determine the relative expression levels of the selected genes in ERK1MO, ERK2MO and standard control MO injected embryos. The obtained qPCR data show that the expression levels of *cdh2*, *mycn, erm *and *snai1a *are down-regulated in both ERK1MO and ERK2MO conditions, compared to standard control MO (Fig. 4A,B,C and 4F), whereas the expression level of *mos *is up-regulated in both ERK1MO and ERK2MO. The analysis of *cfos *and *vegf *confirmed the anti-correlated regulation comparing ERK1 and ERK2 knockdown to standard control MO conditions. Both genes are down-regulated by knockdown of ERK1 and up-regulated by knockdown of ERK2, compared to the expression-level of *fos *and *vegf *in standard control MO treated embryos (Fig. 4D,G).

In summary, the qPCR data confirmed the change in expression levels of the selected genes as observed by microarray analysis for all genes tested, thereby confirming the unique gene expression profiles for ERK1MO and ERK2MO mediated knockdown in early zebrafish development at 4.5 hpf (30% epiboly).

### Gene Ontology (GO) analysis

**Figure 5:**
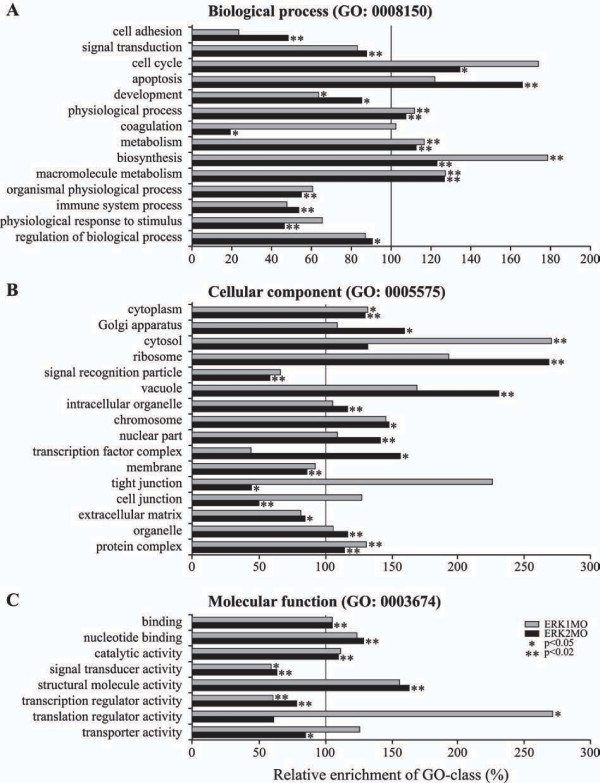
**Statistical comparison of the Gene-Ontology distribution within the gene expression profiles, in ERK1 versus ERK2 knockdown embryos. **(A) Biological process (GO:0008150), (B) Cellular component (GO:0005575) and (C) Molecular function (GO:0003674). ERK1MO and ERK2MO were compared to the whole 22K Agilent chip, based on the Unigene-ID identifiers. The graph depicts the relative fold of enrichment (x-axis) of the statistically selected GO-clusters (y-axis), within the ERK1 and ERK2 knockdown gene-pools. ERK1MO in gray, ERK2MO in black (* = P < 0.05, ** = P < 0.01). Values greater than 1 were considered over-represented, values less than1 are considered as under-represented.

The gene expression signatures of the ERK1 and ERK2 morphants were used to perform gene ontology (GO) analysis. This provides an unbiased biological gene enrichment analysis based on biological properties (GO-terms) assigned per gene. Gene ontologies describe gene products in terms of their associated biological processes (GO:0008150), cellular components (GO:0005575) and molecular functions (GO:0003674) in a species-independent manner. The results of this analysis showed a significant relative over- or under-representation of the number of Unigene IDs in ERK1 versus ERK2 morphants within the GO categories (Fig. 5). For ERK1 and ERK2 knockdown signature sets we obtained remarkable differences in the significantly enriched categories in the highest analyzed GO-level (level 4): for instance 5 vs. 14 enriched GO-terms are associated with Biological processes (Fig. 5A), 3 vs. 15 enriched GO terms are associated with cellular components (Fig. 5B) and 3 vs. 7 enriched GO terms are associated with Molecular functions (Fig. 5C), respectively.

Comparing the ERK1 and ERK2 knockdown signature sets various particular differences in over- or under-represented GO-terms were found. For example, both the GO-terms cell cycle (GO:0007049) and apoptosis (GO:0006915) are significantly enriched upon ERK2 knockdown. However, looking at the gene-lists in more detail inhibitory factors of apoptosis are mostly down-regulated, whereas positive regulators of cell cycle were up-regulated, indicating that apoptosis was not induced by the depletion of ERK2 at 30% epiboly (also see Fig. 2D) confirming our earlier conclusion from the time series. Cell adhesion (GO:0007155) and the cellular component GO terms 'tight junction' and 'cell junctions' are only significantly under-represented in the signature set of ERK2 morphants. Regulation of cell adhesion and the organization of tight- and cell-junctions are crucial for cell migration processes. Specifically for ERK1 knockdown a significantly enrichment of the 'translator regulator activity' (GO:0030528) GO-cluster was found. In contrast, the relative enrichment of this GO term in ERK2 morphants showed an under-representation. A significant overrepresentation of the GO term biosynthesis in ERK1 morphants correlates with these observations.

The GO-enrichment analysis showed that the number of genes within the GO-cluster 'development' (GO:0007275) were significantly under-represented for both ERK1 (19 genes) and ERK2 (136 genes) morphants. From the 19 development-related genes whose expression was affected by ERK1 knockdown, 12 genes (63%) were not found in the ERK2 knockdown signature set. This supports the notion that ERK1 and ERK2 may have distinct functions during embryogenesis by affecting the gene-expression of common and distinct genes sets during vertebrate development.

### GenMAPP Pathways for zebrafish

**Figure 6:**
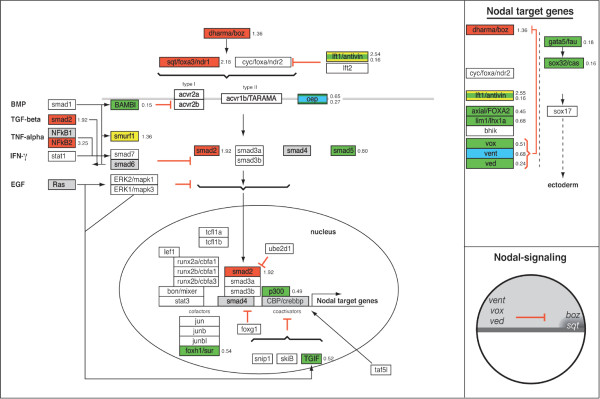
**Analysis of Nodal signaling processes in ERK1MO and ERK2MO gene expression profiles**. The Nodal signaling pathways has been overlaid with gene-expression colour criterion and ratios of gene-expression from the program GenMAPP: yellow = up-regulated by ERK1MO (ratio > 1), blue = down-regulated by ERK1MO (ratio <1), red = up-regulated by ERK2MO (ratio > 1), green = down-regulated by ERK2MO (ratio <1), gray = gene is not present on the Agilent zebrafish 22k microarrays or in the GenMAPP database, white = not significantly changed. The genes that were affected in their expression in both ERK1 and ERK2 morphants show multicolored gene-boxes with the expression ratios for both conditions depicted on the right of the gene; the ration for ERK1 knockdown at the top and ERK2 knockdown below. At the right side of the figure a list of responsive target-genes is listed for the Nodal signaling pathway. The bottom right of the figure shows a small representation of the predicted Nodal signaling activity (dark gray) in the wild type embryos, based on the potential range of signals and the expression patterns and range of antagonists adopted from Schier and Talbot (2005), late blastula stage, lateral view, dorsal to right and animal pole to top.

**Figure 7:**
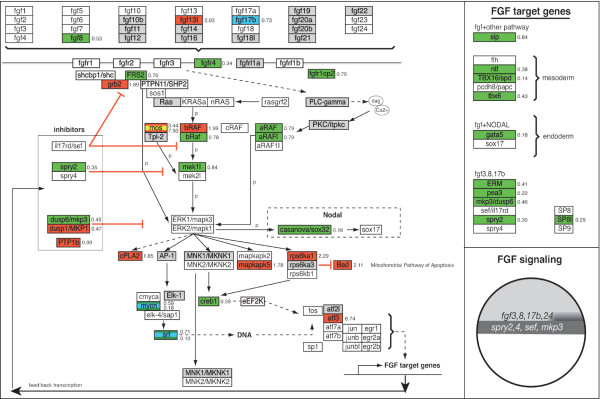
**Analysis of FGF signaling processes in ERK1MO and ERK2MO gene expression profiles**. The FGF signaling pathways has been overlaid with gene-expression colour criterion and ratios of gene-expression from the program GenMAPP: yellow = up-regulated by ERK1MO (ratio > 1), blue = down-regulated by ERK1MO (ratio <1), red = up-regulated by ERK2MO (ratio > 1), green = down-regulated by ERK2MO (ratio <1), gray = gene is not present on the Agilent zebrafish 22k microarrays or in the GenMAPP database, white = not significantly changed. The genes that were affected in their expression in both ERK1 and ERK2 morphants show multicolored gene-boxes with the expression ratios for both conditions depicted on the right of the gene; the ration for ERK1 knockdown at the top and ERK2 knockdown below. At the right side of the figure a list of responsive target-genes is listed for the FGF signaling pathway. The bottom right of the figure shows a small representation of the predicted FGF signaling activity (dark gray) in the wild type embryos, based on the potential range of signals and the expression patterns and range of antagonists adopted from Schier and Talbot (2005), late blastula stage, lateral view, dorsal to right and animal pole to top.

**Figure 8:**
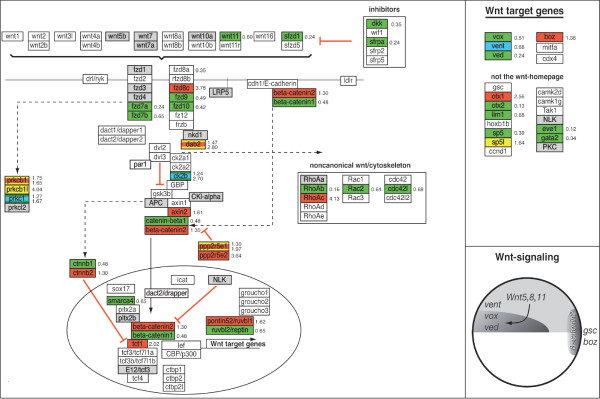
**Analysis of Wnt signaling processes in ERK1MO and ERK2MO gene expression profiles**. The Wnt signaling pathways has been overlaid with gene-expression colour criterion and ratios of gene-expression from the program GenMAPP: yellow = up-regulated by ERK1MO (ratio > 1), blue = down-regulated by ERK1MO (ratio <1), red = up-regulated by ERK2MO (ratio > 1), green = down-regulated by ERK2MO (ratio <1), gray = gene is not present on the Agilent zebrafish 22k microarrays or in the GenMAPP database, white = not significantly changed. The genes that were affected in their expression in both ERK1 and ERK2 morphants show multicolored gene-boxes with the expression ratios for both conditions depicted on the right of the gene; the ration for ERK1 knockdown at the top and ERK2 knockdown below. At the right side of the figure a list of responsive target-genes is listed for the Wnt signaling pathway. The bottom right of the figure shows a small representation of the predicted Wnt signaling activity (dark gray) in the wild type embryos, based on the potential range of signals and the expression patterns and range of antagonists adopted from Schier and Talbot (2005), late blastula stage, lateral view, dorsal to right and animal pole to top.

**Figure 9:**
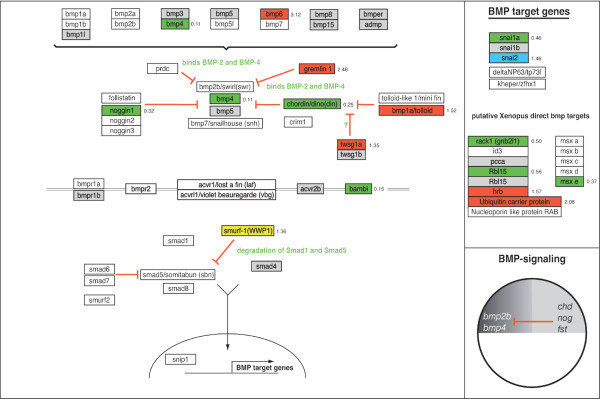
**Analysis of BMP signaling processes in ERK1MO and ERK2MO gene expression profiles**. The BMP signaling pathways has been overlaid with gene-expression colour criterion and ratios of gene-expression from the program GenMAPP: yellow = up-regulated by ERK1MO (ratio > 1), blue = down-regulated by ERK1MO (ratio <1), red = up-regulated by ERK2MO (ratio > 1), green = down-regulated by ERK2MO (ratio <1), gray = gene is not present on the Agilent zebrafish 22k microarrays or in the GenMAPP database, white = not significantly changed. The genes that were affected in their expression in both ERK1 and ERK2 morphants show multicolored gene-boxes with the expression ratios for both conditions depicted on the right of the gene; the ration for ERK1 knockdown at the top and ERK2 knockdown below. At the right side of the figure a list of responsive target-genes is listed for the BMP signaling pathway. The bottom right of the figure shows a small representation of the predicted BMP signaling activity (dark gray) in the wild type embryos, based on the potential range of signals and the expression patterns and range of antagonists adopted from Schier and Talbot (2005), late blastula stage, lateral view, dorsal to right and animal pole to top.

To further analyze putative down stream targets of ERK1 and ERK2 involved in early development, we focused on essential signaling pathways that are involved in early embryonic differentiation and patterning; Nodal, FGF, Wnt and BMP-signaling pathways (Fig. 11) [12]. For our study, we used the signaling pathway analyzing software program, GenMAPP (Gene Microarray Pathway Profiler) [10,11]. This program is designed for viewing and analyzing gene expression data in the context of biological pathways and allows microarray-mediated gene expression signature sets to be displayed on biological (signaling) pathways [10]. In contrast for human and mouse gene expression data-sets, where most signaling pathways are available for this program, there are no GenMAPP pathways based on zebrafish literature available yet for analyzing our gene expression datasets. Therefore, we first generated the *in silico *GenMAPP pathways for the zebrafish Nodal, FGF, (canonical) Wnt and BMP signaling pathways (Fig. 6, 7, 8, 9). This provides a valuable tool for the research community that makes use of zebrafish. The construction of these GenMAPP signaling pathways is based on what is specifically described in literature for zebrafish development, supported by the described knowledge for other vertebrate signaling processes and canonical signaling models, found on the Science's STKE Connections Map Database [14]. Although it is clear that the Nodal, FGF, Wnt and BMP pathways are all interconnected, resulting in a complex signaling network, we performed a pathway-based analysis focusing on separate signaling pathways since the ways these signaling pathways exactly interconnect on a molecular scale is hardly understood yet.

### Pathway Analysis of ERK1MO and ERK2MO mediated knockdown expression profiles

The Unigene ID linked ERK1MO and ERK2MO signature sets that were used for GeneMAPP analysis were not limited by fold change but instead we used all genes that had a combined p-value for changed expression, compared to the standard control morpholino treated embryos, smaller than 10^-5^. As previously mentioned, the number of genes that showed a changed expression in ERK2MO compared to ERK1MO injected embryos was far larger. Therefore, as expected, more genes with changed expression levels were found in the *in silico *GenMAPPs signaling pathways for ERK2MO, than for ERK1MO.

Knockdown of ERK1 did show only one gene (*smurf1*) with a significantly changed expression level within our BMP signaling GenMAPP (Fig. 9). However, more genes were affected in FGF signaling: *fgf17b *(-1.37 fold) the MAPKKK *mos*, (+3.448 fold), transcription factor *cmyc *(-1.71 fold) and *srf *(*serum response factor*, -1.39 fold) showed significant changes in expression. In the nodal pathway, the Nodal antagonist *lft1/antivin1 *(+2.55 fold) and the EGF-CFC co-receptor *oep *(one eyed pinhead, -1.53 fold) were the only components found to be affected in ERK1 morphants. Furthermore, the ventrally expressed Wnt8-mediated organizer inhibitory gene *vent *[15] was down-regulated (-1.46 fold, Fig. 8). Other genes involved in Wnt-signaling affected by ERK1 knockdown were *dab2 *(disabled homolog 2, +1.47 fold), *ck2b *(casein kinase II beta subunit, -1.24 fold) and ppp2r5e1 (Protein phosphatase 2A, regulatory B subunit, B56, +1.30 fold). These genes are also considered to be involved in early embryonic pattering pathways. Two genes involved in regulating gastrulation cell migration, *one-eyed pinhead *(*oep*) and *quattro *[16,17], were altered in expression.

The effect of depletion of ERK2 was far more severe in most of the analyzed signaling processes (Fig. 6,7,8,9). Key components of the FGF-pathway (*fgf8*, *fgfr4 frs2*, *bRaf*, *aRaf *and *mek1l*) and downstream target genes (*erm*, *eve1*, *pea*, *mkp3*, *spry2*, *ntl*, *spt/tbx16 *and *tbx6*) were down-regulated, indicating a block of the FGF-ERK pathway by ERK2 knockdown (Fig. 7). Expression of some of these (mesoderm) target genes is initiated by Nodal. The Nodal-genes like *boz*/*dharma*, *squint*/*ndr1 *and *smad2 *are up-regulated, whereas inhibiting genes lefty1 (*lft1*, -6 fold) and the ventral genes *vox *(-1.9 fold) and *ved *(-4 fold) are down-regulated in ERK2 morphants (Fig. 6). Other nodal signaling mediator genes that are down-regulated are *oep *(-4 fold), *p300 (*-2.03 fold), *foxh1*/*sur *(*schmalspur*, -2 fold) and the negative regulator of TGFβ signaling *TGIF *(-2 fold). The nodal-mediated endoderm gene *sox32*/*casanova*, expressed in the margin, was down-regulated (-6 fold), and also the downstream target-gene axial/foxA2 (-2 and -4 fold). Interestingly, *squint*/*ndr1 *also functions as a positive regulation of fibroblast growth factor receptor signaling pathway [18].

The Wnt ligand *Wnt11 *and receptors (*frz7a, 7b, 8a*, *9 *and *10*) and the central mediator *β-catenin1 *were down-regulated in ERK2 morphants, suggesting a severe inhibitory effect or even complete block of these pathways at this level (Fig. 8). This inhibition of the Wnt pathway is also supported by the up-regulation of axin2/*conductin*, a scaffold protein from the β-catenin destruction complex, responsible for the degradation of beta-catenin [19]. Down-regulation of the putative Wnt-target genes *vox*, *vent*, but also *otx2*, *sp5*, and *lim1 *further support impaired Wnt-signaling. However, ERK2 knockdown also led to the down-regulation of the inhibitors *dkk1 *and *sfrp1*, and up-regulation of the intracellular Wnt-signaling components *fxd8c*, *dab2*, β-*catenin2 *and *tcf1*.

The effect of ERK2 knockdown on BMP signaling is also complex, as *bmp4 *is up-regulated whereas *bmp1a*/*tolloid *and bmp6 are down-regulated (Fig. 9). This opposing effect is also found in the BMP antagonists, as *chordin *(*chd*) and the ventrally expressed membrane bound bmp-inhibitor *bambi *were down-regulated, whereas a different BMP antagonist *gremlin *is up-regulated. Adding to this complexity is the fact that the agonist *twisted gastrulation *(*twsg1a*) is up-regulated. The results clearly show that that dorsal-ventral patterning and also mesoderm patterning is severely affected but it is difficult to speculate about the downstream effects of all these changes of expression in the BMP pathway.

### Biological confirmation of Pathway Analysis based prediction

**Figure 10:**
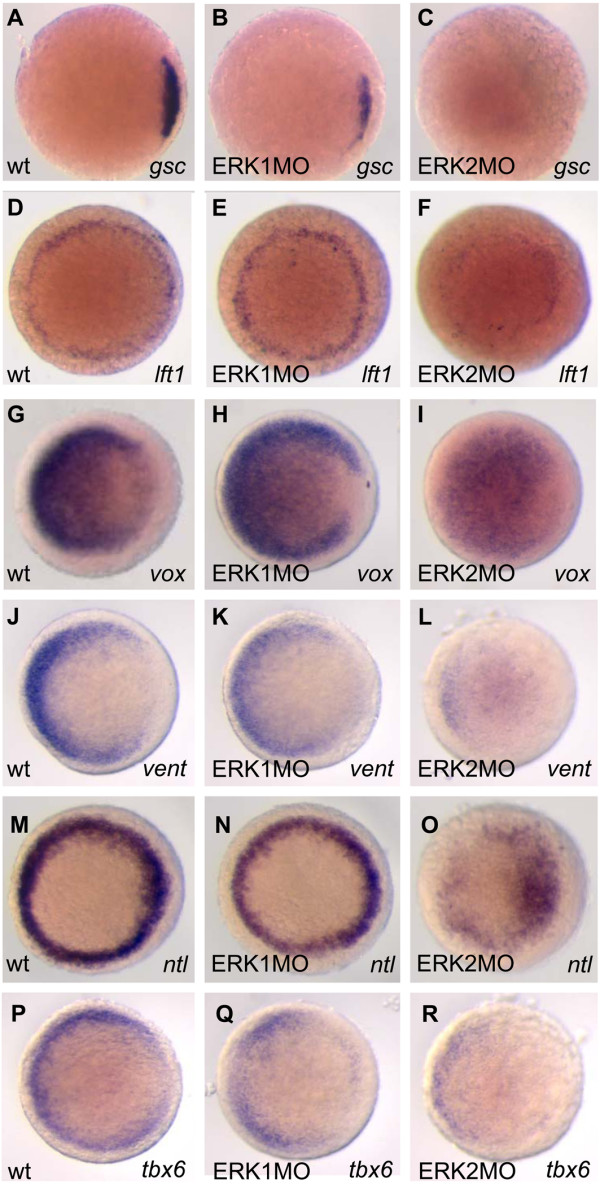
**Effects of ERK1 and ERK2 knockdown affect developmental signaling pathways confirmed by whole mount in situ hybridization**. The zebrafish embryos were injected with 3.4 ng ERK1MO (B,E,H,K,N,Q) or ERK2MO (C,F,I,L,O,R) and in situ expression patterns, were compared to wild type embryos (A,D,G,J,M,P). The embryos were fixed at 4.5hpf, processed for whole mount in situ hybridization, and imaged (animal pole view, dorsal to right). A,B,C: *goosecoid *(gsc, presumptive shield/dorsal organizer); D,E,F: *lft1/antivin*, (blastula margin); G,H,I: *vox *(expressed in blastula, but the dorsal most region); J,H,L *vent *(ventral blastula margin); M,N,O, *notail *(*ntl*, blastula margin); P,Q,R,: *tbx6 *(margin)

**Figure 11:**
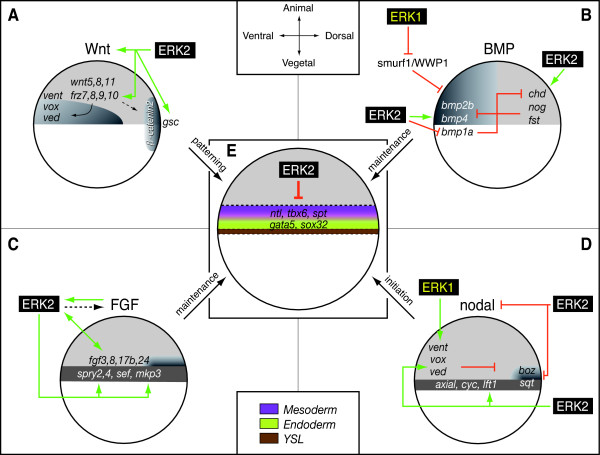
**ERK1 and ERK2 knockdown differently affect signals involved in patterning of the early embryo**. (A-D): Schematic representation of the effects of ERK1 and ERK2 knockdown on the activities of Nodal, FGF, Wnt and BMP signaling pathways in late blastula embryos. (E): effect of ERK2 knockdown (ERK2MO) on early embryonic mesendoderm differentiation. The representation of predicted signaling activity in the wild type embryos is based on the potential range of signals, the expression patterns and range of antagonists, adopted from Schier and Talbot (2005). The combined signaling activities from these pathways are responsible for the differentiation and fate-map of the late blastula/early gastrula stage of the zebrafish embryo (E, late blastula stage, lateral view, dorsal to right, animal pole to top). In the zebrafish embryo, dorsal ventral patterning starts as early as the 128-cell stages by accumulation of β-catenenin at the nuclei of the dorsal cells, rapidly followed by the expression of *goosecoid *(A). Soon after mid-blastula transition, β-catenin also activates the expression of a number of zygotic genes, including *chordin*, *bozozok *and *squint *(A and D), and FGF signals (C). These genes act to inhibit the action of ventralizing factors or induce mesoderm and endoderm cell fates at the dorsal side. Subsequently, the expression of these genes quickly spreads over the complete margin (E). To establish a mesodermal zone, induction processes occur at the animal – vegetal axis. Complex signaling processes are used by the embryo to induce mesoderm. In a over-simplified manner, it can be said that Nodal (D) signaling is involved in initiation of mesoderm formation, FGFs (C) and Wnt (A) are involved in maintaining the mesoderm state and BMPs (B) are involved in further patterning of the mesoderm [33]. Knockdown of ERK1 (ERK1MO) resulted in an increased expression of the BMP-inhibiting protein *smurf1*/*wwp1 *and the ventrally expresses gene *vent *(A). In addition also the mesoderm marker *tbx6 *showed a reduced dorsal expansion of its expression domain (Fig. 7K). Combined, this indicates a reduction of ventral signaling, possibly leading to a mild dorsalization of ERK1 knockdown embryos. ERK2 knockdown (ERK2MO) promotes Nodal signaling by repressing the expression of Nodal inhibitors (*vox*, *vent*, *ved *and *lft1*) (D). Furthermore, it perturbs FGF signaling (repression of *fgf8 *and components of the RAS-ERK pathway and down regulation of its target genes) and Wnt signaling (repressed expression of frizzled receptors and key components of the Wnt pathway). In addition, perturbed BMP signaling results in incorrect patterning of the mesoderm (B). In summary, this shows that mesendoderm differentiation is still initiated by Nodal signaling (D), but mesendoderm maintenance by FGF and Wnt signaling is defected. This results in reduced expression of mesoderm (*ntl*, *tbx6 *and *spt*) and endoderm (*gata5*, *sox32*) marker genes (B, C and E), showing that ERK2 is essential for mesendoderm differentiation (E).

To confirm predicted effects of the GenMAPP pathway analysis experimentally and to add information on the localization of expression, we performed whole mount in situ hybridization on ERK1 and ERK2 morphants at 30% epiboly with marker genes regulated by Nodal, BMP, Wnt and FGF (Fig. 6, 7, 8, 9 and Fig. 11). Different components of the Wnt-β-catenin pathway showed lower expression levels in ERK2 morphants. We showed that *goosecoid *(gsc) [20], a downstream marker gene for the Wnt pathway at early developmental stages (Fig. 10A–C) is not expressed in the ERK2 morphants. Knockdown of ERK1 did lead to a significant effect on the expression of *gsc*, but after knockdown of ERK2 no expression of *gsc *was detected by whole mount in situ hybridization. This confirms that canonical Wnt signaling was severely affected in ERK2 morphants, preventing subsequent expression of the Wnt-target gene *gsc*.

The *lefty 1 *(*lft1, antivin1*) gene is a member of the TGF-beta super-family that regulates left-right axis formation during embryogenesis via antagonistic activity against nodal, another TGF-beta super-family member. Expression starts at blastula stage, immediately after initiation of zygotic transcription, and is localized in the whole blastula margin at late blastula – 30% epiboly stage [21]. Whole mount in situ hybridization with lefty1 probe (Fig. 10D–F) at 30% epiboly shows a possible increase of *lefty1 *expression in ERK1 morphants (Fig. 10E), but the decrease of expression in ERK2 morphants (Fig. 10F) was clearly visible. As *lefty1 *is both an antagonists of Nodal signaling as well as a Nodal responsive gene, an increase of *lft1 *expression could mean that the signaling is sufficient and must be inhibited (ERK1MO), like in a wild type situation. A decrease in expression would mean that Nodal signaling not yet sufficient. Expression of mesoderm-genes in the margin indicates that (nodal mediated) mesoderm initiation took place in ERK2 morphants, however at a much reduced level (Fig. 10O,R).

In zebrafish, *vox *and *vent *interact with *bozozok *(*boz*), which is the earliest expressed dorsal-specific gene, and studies of *boz *embryos and the effects of ectopic *boz *expression indicate that it functions at the top of a hierarchy. *Vox *and *vent *are proposed to be repressors of *boz *expression since ectopic *vox *and *vent *eliminated the appearance of *boz *to establish the dorsal organize [15]. The expression signatures from the ERK1 and ERK2 morphants revealed that *vox *expression was not significantly changed in ERK1 morphants, but was down-regulated in ERK2 morphants., whereas for *vent *-expression this seemed to be opposite, as its expression was down-regulated in ERK1 morphants, but not significantly changed in ERK2 morphants (Fig. 6). The expression patterns of these genes revealed a possible reduction of *vox *expression in ERK1 morphants, which was more obvious on the putative dorsal side of the embryo where a clear cap was observed. The expression of *vent *was also reduced in ERK1 morphants and did not extend as far dorsally (K) compared to wild type embryos (J), indicating a mild dorsalization of ERK1 morphants. In ERK2 morphants, *vox *expression seemed to be reduced to a greater extend at the ventral side, but in the rest of the blastula the reduction of *vox *expression was not as significant and expression of *vent *was only detected at the ventral side of the blastula margin (L). In support of these finding, the expression of *boz *was found also to be up-regulated (+1.4 fold) in ERK2 morphants. Combined, these findings confirm that knockdown of ERK2 leads to impaired Wnt-mediated *vox *and *vent *expression which is reported to be involved in mesoderm patterning and maintenance.

The zebrafish *ntl *gene is, like the *tbx6 *gene, a member of the *Brachyury*-related T-box family of genes. *Notail *(*ntl*/*brachyury*) is involved in mesoderm development, as described in the legend to figure 11. At 30% epiboly *ntl *is expressed in the blastula margin [22]. This expression is synergistically regulated by FGF and Nodal signaling pathways [23,24]. Both of these pathways show a negative regulation in the ERK2 morphants, as shown by the GenMAPP analysis (Fig. 6, 7, 8, 9). The negative effect on these pathways and the array-data itself suggested a down-regulation of the *ntl*-gene upon ERK2 knockdown, and was confirmed by whole mount in situ experiments (Fig. 10M–O). The *ntl *gene expression in ERK1 morphants was comparable to expression in wild type embryos, but *ntl *expression was decreased in ERK2 morphants. Strikingly, expression of *ntl *was not constant in the marginal ring, as stronger expression was detected in the putative dorsal side of the ERK2 morphants.

*Tbx6 *is exclusively expressed in the ventral mesendoderm and its expression is linked to ventral mesoderm specification [25]. In ERK1 morphants the in situ hybridization experiment showed that *tbx6 *expression was not extended as far dorsally as in wild type embryos, as *tbx6 *expression at the putative dorsal side of these embryos was severely reduced (Fig. 10P,Q). In ERK2 morphants, *tbx6 *expression was greatly reduced and was only detected at the ventral margin (Fig. 10R). In ERK2 morphants *tbx6*-expression an even more severe reduction of *tbx6 *expression was down-regulated (-2.3 fold).

The obtained results by whole mount in situ hybridization using *gsc*, *lft, vox, vent, ntl *and *tbx6*, confirm or support the predictions made by the GenMAPP analysis, as the changes in their expression levels are in agreement with the predictions obtained by the signaling pathway analysis of the microarray data.

## Discussion

Specific functions of most proteins in vertebrate development remain elusive because of potential redundancies. In this manuscript we present a case study that indicates that the combination of micro-array analysis and targeted knockdown of essential embryonic genes in zebrafish can provide new insights in the specific targets of key regulators of development. For this study we have chosen the mitogen activated protein kinase members ERK1 and ERK2 because they are involved in virtually all eukaryotic cellular processes and signaling networks but still little is known of their specific functions in development. The proteins show high amino acids identity and have redundancy potential; however this does not exclude specific target genes.

These archetypal signaling proteins are good examples for showing the power of this approach since the upstream activation pathways for ERK1 and ERK2 are highly similar, and many of their known downstream targets are common. In contrast to this, mice and zebrafish studies indicate distinct roles for both ERKs in cellular proliferation, oncogenic transformation and development. A major bottleneck for further studies is that relatively few *in vivo *downstream targets of these kinases and upstream activators such as MEK1 and MEK2 have been identified conclusively. Our manuscript uses microarray technology and bioinformatics to document the functional differences between the ERK1 and ERK2 proteins at the transcriptome level at different time points during zebrafish development. The obtained data is projected on a model of our current knowledge of several developmental signaling pathways. This gives new mechanistic insights in how ERK signaling is functioning and integrates with other known effectors of vertebrate embryogenesis.

### ERK1 and ERK2 target distinct genes during early zebrafish development

Comparison of the gene expression profiles of the ERK1 and ERK2 morphants during early embryogenesis, with standard control MO injected embryos as a shared reference, showed specific gene expression profiles. Distinct gene expression signatures were obtained for ERK1 and ERK2 knockdown embryos, proving that both ERK1 and ERK2 target specific gene pools during zebrafish embryogenesis (Fig. 2). The gene expression profiles of ERK1 and ERK2 knockdown embryos showed sets of genes that were commonly regulated, but also genes that was regulated in an anti-correlated manner, involved in cell cycle, proliferation, cell differentiation, metabolism, cytoskeleton dynamics, signal transduction, migration and transcription. This observation is in line with the notion that ERK1 and ERK2 may have specific downstream targets, as proposed in a model by Alison Lloyd [3], mainly based on the work of Vantaggiato et al. [2], where they show that co-transfection of either *erk1 *or *erk2 *with an oncogenic form of Ras, has different effects on proliferation and Ras-induced transformation. In addition to this, *erk1*-/- mice are viable and fertile [5], whereas disruption of *erk2 *is embryonic lethal due to defects in placenta formation, trophectoderm and mesoderm differentiation [6,26]. Activation of the upstream signaling of ERK have also shown a role for this pathway in diseases such as cardio-faciocutaneous syndrome and carcinogenesis. Furthermore a developmental role of MEKs was shown: *mek2 *knockout mice are phenotypically normal, whereas *mek1 *knockout mice die at embryonic day (E) 10.5 due to abnormal development and insufficient vascularization of the placenta [27,28]. Studies in mouse ES cells showed that ERK2 disruption does not interfere with proliferation of undifferentiated ES cells [29]. Although *erk1*-/- mice present normal mesoderm differentiation, they do show defective adipocyte formation [30]. The exact mechanisms for ERK signaling in adipocyte development, likely via the adipocyte-specific transcription factor peroxisome proliferator-activated receptor (PPAR)γ, is still under debate [29,31,32]. The significant over-representation of the GO-terms 'metabolism', 'biosynthesis' and 'macro-molecule biosynthesis' (Fig. 5) may indicate that also in zebrafish adipocyte-development is ERK1 dependent which would be in line with our suggestion, that role for ERK1 becomes more dominant at later developmental stages (Fig. 2). However, further studies at even later (larval) stages of development need to be performed to confirm this hypothesis.

The higher number of genes affected by the knockdown of ERK2 is in agreement with the severe phenotype of ERK2 knockdown embryos (Fig. 1 and Fig. 2). In order to understand the severe effects of ERK1/2 knockdown, we have to consider the results in the context of the known signaling pathways that govern developmental programs as proliferation, cell migration and differentiation processes. Therefore we first generated *in silico *signaling pathway, for analysis of important signaling pathways involved in early vertebrate development and performed analysis on the ERK1 and ERK2 transcriptome signatures using the GenMAPP software program. These include the Nodal, FGF, Wnt and BMP signaling pathways.

### ERK1 and ERK2 are involved in different developmental processes

For biological interpretation of the obtained expression profiles, analysis of gene ontology (GO) was used to indicate processes that are likely to be affected. Different gene ontology clusters showed a relative enrichment in ERK1 versus ERK2 knockdown gene expression signatures. Since the annotation of the zebrafish genome is the limiting factor in assigning biological functions we have focused on gene ontologies that are relatively well known and have further supported the analyses by manual annotation of our signature sets. This led for instance to the observation that the Biological GO-clusters "development" was significant under-represented for both ERK1 and ERK2 knockdown. More detailed analysis was performed using the signaling-pathway based GenMAPP gene map annotator and pathway profiler program. By performing complete gene expression profiles (p < 10^-5^) without a fold-change cut-off in pathway analyses, we address both primary and secondary effects related to ERK knockdown from a morphogenetic perspective. Our observations led us to propose a model for distinct effects of ERK1 and ERK2 knockdown in developmental signaling processes, by effecting common as well as distinct genes (Fig. 2G). Early embryo developmental processes include mesoderm formation, endoderm formation dorsal-ventral pattering, anterior-posterior patterning and gastrulation movements. To establish a mesodermal zone, next to the dorsal-ventral patterning, also induction processes occur at the animal – vegetal axis. Complex signaling processes are used by the embryo to induce mesoderm, as nicely reviewed by Kimelman (Nature reviews 2006) [33]. Based on literature data it is possible to interpret the observed gene-expression profiles and analyze the knockdown effects in the context of known signaling pathways underlying these processes (Fig. 6, 7, 8, 9 and Fig. 11).

Stringent knockdown conditions, as applied in this array-based study, showed that in ERK1 morphants the ventrally expressed patterning gene *vent *was down-regulated, but also the BMP inhibitory gene *smurf1 *was up-regulated, possibly responsible for inhibition of BMP signaling on the ventral side (Fig. 11B,D). This may lead to a dorsalization of ERK1 knockdown embryos. Surviving ERK1 morphants showed a tailless phenotype at 24 and 48 hpf [see Additional file 2]. This supports a block of bmp-signaling, as tail formation is combinatory regulated by BMP and FGF signaling since mutant embryos for bmp2b fail to form tails [34] and embryos with impaired FGF-signaling show tailless phenotypes. In addition, it is important to note that also genes involved in regulating gastrulation cell migration were altered in expression (*oep *and *quattro*) [16,35].

### ERK2 signaling is essential for the maintenance of the mesendodermal cell fates

In ERK2 morphants no active MAPK was detected at the margin at 4,5hpf (data not show) suggesting that Ras-Raf-MEK-ERK dependent FGF signaling and subsequent downstream signaling was blocked. FGF signaling acts as a competence factor for cells to respond to Nodal mediated mesoderm induction. As our data show that ERK2 morphants are severely impaired in both FGF and Wnt signaling it is likely that mesoderm progenitor cells in the margin are affected in the maintenance of the mesodermal cell fates (Fig. 11 panel E). However, it has been reported that Nodal and FGF pathways interact through a positive regulatory loop and synergize to maintain mesodermal cell populations [36], in addition FGF signaling negatively regulates Nodal-dependent endoderm induction in zebrafish [37]. This would suggest that Nodal-mediated initiation of mesoderm differentiation is still present, but the maintenance of the mesoderm, mediated by FGF and Wnt, is affected.

Drosophila, FGF-dependent ERK activation was shown to be required for proper mesoderm dispersal [38-40]. In *Xenopus*, ERK2 was shown to be required for mesoderm differentiation [41]. Mouse *erk2-/- *embryos also fail to form mesoderm at E6.5 and E7.5 based on histological criteria, but *erk2-/- *embryonic stem cells were still capable of forming mesoderm. However, treatment of these ES cells with the MAPK inhibitor PD184352 decreased total ERK activity in these cells and expression of the mesoderm marker *brachyury*/*ntl *(essential for posterior mesoderm and axis formation) [26]. Our gene expression profiling shows that ERK2 plays a role in mesoderm development based on additional mesoderm markers (e.g. *spt*/*tbx16*, *tbx6*), but importantly also by addressing the upstream signaling mechanisms involved in mesoderm initiation and maintenance. It should be noted that ERK-activation is not only mediated by FGF signaling, but also influenced by other growth factors (PDGF, VEGF), G-protein coupled receptor signaling and hormone- and Ca^2+ ^signaling pathways. A nice example that demonstrates the complexity of interconnections, redundancy and crosstalk between the different pathways is the work of Poulain et al, (2006) showing that combinatorial Nodal, FGF and BMP signaling regulates endoderm formation in zebrafish. These authors also reported that activation of FGF-signaling or injection of constitutive active (rat) ERK2 lead to phosphorylation of SOX32 and repression of the endoderm marker *sox17*. However, in our study, ERK2 morphants showed a reduced expression of the upstream Nodal responsive genes *gata5*, *sox32 and sox17*. These genes are normally expressed in presumptive endoderm progenitor cells in the margin at 4,5 hpf [42]. This suggests that depletion of ERK2 also affects endoderm differentiation (Fig. 11). Follow-up experiments, using different times of development in combination with chromatin immuno-precipitation (chIP-chip) methodology will be needed to further understand the crucial function of ERK2 in mesendoderm development and determine specific target genes.

## Conclusion

Our analysis of the gene expression microarray data revealed that ERK1 and ERK2 knockdown affected a set of common, as well as specific downstream genes. Interestingly, we also discovered a set of genes with anti-correlated expression. The gene ontology analyses show that ERK1 and ERK2 have specific roles in embryogenesis and target distinct gene sets involved in vertebrate development, confirming the embryonic knockdown phenotypes. These gene sets are large and considering the early embryonic time points of analyses, most likely include many direct transcriptional targets at least at the oblong stage. At later stages we expect to have identified also several secondary effects that are due to phenotypic changes. The signaling pathway analysis on the ERK1 and ERK2 transcriptome signatures using the GenMAPP software program for analysis of BMP, FGF, Nodal and Wnt signaling pathways indicated distinct roles for these MAP kinases. For ERK1 knockdown we identified a connection with genes involved in dorsal-ventral patterning and subsequent embryonic cell migration. For ERK2 knockdown we identified a connection with genes involved in mesoderm and endoderm initiation, differentiation and patterning. The outcome of the predictions for ERK2 knockdown on developmental signaling were confirmed by the observed effects on mesoderm and endoderm patterning and subsequent whole mount in situ hybridization experiments. Our results demonstrate the strength of gene expression profiling of morpholino knockdown embryos in combination with versatile bioinformatics tools in order to show common functions as well as distinct functions for highly related signaling proteins such as ERK1 and ERK2.

## Methods

### Zebrafish Morpholino knockdown experiments

Zebrafish embryos were injected at the one-cell stage with 1 nl of the solubilized compounds in 1× Danieau's buffer [58 mM NaCl, 0.7 mM KCl, 0.4 mM MgSO_4_, 0.6 mM Ca(NO_3_)_2_, 5.0 mM HEPES; pH 7.6] containing 1% Phenol red solution (Sigma). Definition of stages was according to Kimmel et al. At 1K-stage (3hpf), embryos with a red animal pole were selected as positive-injected embryos.

To block translation of the ERK1 or ERK2 protein, 0.4 mM (3.4 ng) morpholinos (MOs) were injected per embryo. MOs were targeted against the 5'-UTR of the respective mRNAs (GeneTools Philomath, OR, USA): ERK1-MO, 5'-TCTGTCCGCAAATCGTCGCCTTCGC; ERK2-MO, 5'-CACCCAAAAGCACCAGG AAAAGCTC. As a control, the standard control morpholino standard control MO 5'-CCTCTTACCTCAGTTACAATTTATA was used at the same concentration. Injected embryos were kept at 28°C until desired stages, until sacrifice.

### RNA isolation from zebrafish embryos

The zebrafish embryos were homogenized in liquid nitrogen and total RNA was extracted using Trizol reagent (Invitrogen) according to the manufacturer's instructions. To remove genomic DNA, RNA samples were incubated at 37°C for 15 min with 10 units of DNaseI (Roche). The RNA samples were purified using the RNeasy kit (Qiagen) according to the RNA Cleanup protocol. Total RNA concentrations were determined spectrophotometrically using a Nanodrop ND-1000 (Isogen Life science). Optical density A260/A280 ratios of all samples ranged from 1.8–1.9, indicating high purity.

### Experimental design, Labeling and Hybridization of Agilent 22K-microarrays

A total of 19 Agilent 22K-microarray hybridizations were performed for this gene expression profiling study of ERK1 versus ERK2 knockdown during development. A minimum of 2 independent biological replicates were analyzed for each biological sample In the case of ERK1 at 80% epiboly and ERK2 at 30%- and 80% epiboly, additional technical replicate were hybridized for each biological replicate, including dye swaps. For each biological sample, a number of 70–100 morpholino injected embryos were collected. The RNA from standard control MO injected embryos was labeled with Cy3 and those of ERK1MO and ERK2MO injected embryos were labeled with Cy5, using the Agilent Low RNA input linear amplification kit. Hybridization and scanning were performed according to standard Agilent protocol by Service XS (Leiden, the Netherlands).

### Data analysis of Agilent 22K-microarrays

Feature Extraction also performed by Service XS using Agilent FE 8.5 software. Our data has successfully completed the curration protocol by MIAMExpress in the EBI public Array-express database [43]. Subsequent analysis was performed using the default settings implemented in Rosetta Resolver v 7.0 for an error modeling-based normalization. For the analysis and detailed annotation shown in the Venn diagrams and bar-graphs, the combine p-value per gene had to be 10e-5. For the annotated tables we focused on the genes that were most significantly affected. For that selection we used the following criteria: the absolute fold change should be at least 1.5 in each independent replicate; and the p-value provided by the error-model taking into account all hybridizations combined must be smaller than 10^-5 ^to compensate for multiple testing false positives.

For Gene Ontology analysis, the Unigene ID-linked gene expression signature sets of the ERK1 and ERK2 morphants were uploaded into the GeneTools eGOn V2.0 web-based gene ontology analysis software (explore Gene Ontology, database build #97) [44]. These signature sets comprised 575 Unigene IDs in the case of ERK1 morphants and 2987 Unigene IDs in the case of ERK2 morphants were compared to the complete set of 21485 Unigene IDs linked probes from the Agilent 22K zebrafish microarray chip (Biological Process; 6036 Unigene IDs, Molecular Function; 6322 Unigene IDs and Cellular Component; 5606 Unigene IDs). We determined the significantly over- or under represented Gene Ontology clusters in the ERK1MO and ERK2MO Unigene ID linked signature sets (p-value < 0.05 or 0.02). The number of GO-terms was reduced by excluding GO clusters with high similarity in representative genes. To ensure statistical relevance, also the GO-clusters that contained less than 10 Unigene IDs were also removed. The relative fold of gene-enrichment within the ERK1- and ERK2-morphant signature sets was calculated for the selected GO-terms.

For the tables used for GeneMAPP analysis we took a less stringent approach not limiting the number of genes by fold change, therefore using all genes that had a combined p-value smaller than 10^-5^. In this approach we focus on transcriptional effects that can be linked to the phenotypic changes as a result of pathway blocking by ERK knockdown.

### cDNA synthesis and Quantitative PCR

cDNA synthesis was performed using a TGradient Thermocycler 96 (Whatman Biometra) according to the manufacturer's instructions. RNA samples were identical to those used for microarray hybridization. Reactions were performed in a 20 μl mixture of 150 ng RNA, 4 μl of 5× iScript Reaction mix (Bio-Rad) and 1 μl of iScript Reverse Transcriptase (Bio-Rad). The reaction mixtures were incubated at 25°C for 5 min, 42°C for 30 min, and 85°C for 5 min.

Quantitative real-time PCR was performed using the Chromo4 Four-color Real-time PCR detection system (Bio-Rad laboratories, Hercules, CA) according to the manufacturers' instructions. Gene-specific primers for quantitative real-time PCR were designed, using Beacon Designer software, to generate single gene-specific amplicons of 75–150 nucleotides. Reactions were performed in a 25 μl volume comprised of 1 μl cDNA, 12.5 μl of 2× iQ SYBR Green Supermix (Bio-Rad) and 10 pmol of each primer. Cycling parameters were 94°C for 3 min to activate the polymerase, followed by 40 cycles of 94°C for 15 sec and 59°C for 45 sec. Fluorescence measurements were taken at the end of each cycle. Melting curve analysis was performed to verify that no primer dimers were amplified. All reactions were done in duplicate or triplicate and the threshold cycle CT values were plotted against the base 10 log of the amount of cDNA by using Opticon Monitor 3.1 (Bio-Rad) according to the manufacturer's instructions. For evaluation of PCR efficiencies of all primers sets standard curves were generated using serial diluted cDNA samples (dilution factors of 1, 5, 25, 125 and 625) and strong linear correlations between the CT values and the log of input cDNA amount were obtained, indicating correlation coefficiencies ranging from 98% to 101%. Data were normalized using the Genex macro provided by Bio-Rad.

The expression level were tested for *cdh2 *(NM_131081), *mycn *(NM_212614), *erm *(NM_131205), *cfos *(NM_205569), *mos *(NM_205580), *snai1a *(NM_131066) and *vegf *(NM_131408) on the same RNA samples used for the array analysis: 0.4 mM (= 3.4 ng/embryo) ERK1MO, ERK2MO and standard control MO injected embryos, collected at 30% epiboly. α-actin was taken as reference and it showed unchanged expression level between standard control MO injected and ERK1MO or ERK2MO injected embryos. Sequences of forward and reverse primers were 5'-CGAGCAGGAGATGGGAACC-3' and 5'-CAACGGAAACGCTCATTGC-3' for β-actin (accession no. AF057040).

Cdh2; qP1fw 5'-ACAAGAAGCAGAAGTGTGTGAGC-3' and qP2rv AGCGTAGGGTCCAGCGTTG-3',

*Mycn*; qP1fw 5'-GAGGATGATGAGGAAGATGATGAAG-3', qP2rv 5'-CCTGCCTGAGAGTTGGAGAC-3',

*erm*; qP3fw, 5'-TCCACCAACTCTCAATCAAACAGG-3' and qP4rv 5'-AGATGGGCTTCTCCGTCATACC-3',

*cfos*; (NM_205569) qP1Fw 5'-TGACCTGGAGCCGCTTTGC-3' and qP2rv 5'-GGTAGGTGAACATGAAGGAAGACG-3',

*mos*; (NM_205580) qP1fw 5'-CCCTCACCAATCCCCGTCAC-3' and qP2rv 5'-GAGCCTGTGTGCGACTTTACC-3',

*snai1a*; qP3fw 5'-TCCTGCCCACACTGTAACCG-3' and qP4rv 5'-GCGACTAAAGGTGCGAGAGC-3',

*vegf*; qP1fw 5'-GCGGCTCTCCTCCATCTG-3' and qP2rv 5'-ACATCCATGAAGGGAATCACATC-3'.

### Whole mount in situ hybridization

Embryos were fixed overnight in 4% paraformaldehyde in PBS at 4°C and in situ hybridization was performed as described previously [45] using described probes for *gsc*, *lft1/antivin, vox, vent, ntl and tbx6*.

## Authors' contributions

GK Was involved in all experiments, experimental design and bioinformatics analyses. He co-drafted the manuscript, made revisions critically for important intellectual content and submitted the data to the Miamexpress database. MC uploaded data in the Rosetta Resolver database and assisted in bioinformatics analyses. SH carried out the labeling reactions for micro-array analyses and performed Q-PCR experiments. ES-J performed the western blot analyses, co-drafted the manuscript, made revisions critically for important intellectual content and participated in design and coordination. HS co-drafted the manuscript, made revisions critically for important intellectual content and gave final approval for the final version to be published and participated in design and coordination.

## Supplementary Material

Additional file 1 Additional data is submitted as tables S1 to S6 consists of the ERK1 and ERK2 knockdown commonly and anti-correlated regulated probes (table S1 to S4), containing the assigned gene designations (Unigene, accession number and sequence name), the fold of the changed expression and p-value (smaller than 10^-5 ^to compensate for multiple testing false positives) for these genes. The tables S5 and S6 contain genes selected by a stringent selected that were only found in either ERK1MO or ERK2MO gene-pools were manually annotated and assigned gene designations as listed in. **Table S1 – ***Anti-correlated regulated genes1: ERK1MO up-regulated, ERK2MO down-regulated***. Table S2 – ***Anti-correlated regulated genes2*: *ERK1MO down-regulated, ERK2MO up-regulated***. Table S3 – ***Commonly down-regulated genes by ERK1or ERK2 knockdown at 30% epiboly***. Table S4 – ***Commonly up-regulated genes by ERK1or ERK2 knockdown at 30% epiboly***. Table S5 – ***ERK1 knockdown specific genes at 30% epiboly, filtered by a 1.5 fold up- or down- regulation per experiment and a common P-value of 10*^-5 ^**. Table S6 – ***ERK2 knockdown specific genes at 30% epiboly, filtered by a 1.5 fold up- or down- regulation per experiment and a common P-value of 10*^-5^ Click here for file

Additional file 2 **ERK1 knockdown phenotype at 24 and 48hpf**. Images show representative examples of surviving ERK1 morpholino injected embryos with a tailless phenotype at 24 and 48hpf. Click here for file
