# Supplementary material for: ERK1 and ERK2 MAPK are key regulators of distinct gene sets in zebrafish embryogenesis
Source: BMC Genomics. 2008 Apr 28;9:196. doi: 10.1186/1471-2164-9-196 (PMC2390552; doi:10.1186/1471-2164-9-196)
Supplement: Additional file 1 — Additional data is submitted as tables S1 to S6 consists of the ERK1 and ERK2 knockdown commonly and anti-correlated regulated probes (table S1 to S4), containing the assigned gene designations (Unigene, accession number and sequence name), the fold of the changed expression and p-value (smaller than 10-5 to compensate for multiple testing false positives) for these genes. The tables S5 and S6 contain genes selected by a stringent selected that were only found in either ERK1MO or ERK2MO gene-pools were manually annotated and assigned gene designations as listed in. Table S1 – Anti-correlated regulated genes1: ERK1MO up-regulated, ERK2MO down-regulated. Table S2 – Anti-correlated regulated genes2: ERK1MO down-regulated, ERK2MO up-regulated. Table S3 – Commonly down-regulated genes by ERK1or ERK2 knockdown at 30% epiboly. Table S4 – Commonly up-regulated genes by ERK1or ERK2 knockdown at 30% epiboly. Table S5 – ERK1 knockdown specific genes at 30% epiboly, filtered by a 1.5 fold up- or down- regulation per experiment and a common P-value of 10-5 . Table S6 – ERK2 knockdown specific genes at 30% epiboly, filtered by a 1.5 fold up- or down- regulation per experiment and a common P-value of 10-5 [file 1471-2164-9-196-S1.doc]

**SUPPLEMENTAL DATA**

**Table S1;** *Anti-correlated regulated genes1: ERK1MO up-regulated, ERK2MO down-regulated*

| **Unigene**  Nov 2006 | **Unigene**  old | **Accession** | **Sequence Name(s)** | **Fold Change**  **ERK1 MO** | **P-value**  **ERK1 MO** | **Fold Change**  **ERK2 MO** | **P-value**  **ERK2 MO** |
| --- | --- | --- | --- | --- | --- | --- | --- |
| [Dr.32651](http://www.ncbi.nlm.nih.gov/UniGene/clust.cgi?ORG=Dr&CID=32651) | Dr.42219 | TC279036 | cyt1 | **2.95** | 1.75E-08 | **-4.53** | 6.02E-06 |
| Dr.24982 | Dr.24982 | BC066622 | zgc:56585 | **11.69** | 4.94E-18 | **-27.58** | 2.19E-12 |
| [Dr.31402](http://www.ncbi.nlm.nih.gov/UniGene/clust.cgi?ORG=Dr&CID=31402) | Dr.31402 | TC293163 | zgc:110552 | **5.97** | 4.08E-17 | **-4.36** | 1.15E-18 |
| [Dr.364](http://www.ncbi.nlm.nih.gov/UniGene/clust.cgi?ORG=Dr&CID=364) | - | TC271110 | lhx1a | 1.31 | 9.59E-06 | **-2.35** | 6.01E-08 |
| Dr.75603 | Dr.10279 | NM_212996 | h3f3a | 1.44 | 6.53E-06 | -1.52 | 9.38E-14 |
| Dr.79332 | Dr.9710 | AI883431 | zgc:136864 | **10.91** | 1.47E-23 | **-4.01** | 2.33E-06 |
| [Dr.12618](http://www.ncbi.nlm.nih.gov/UniGene/clust.cgi?ORG=Dr&CID=12618) | - | TC273224 | efnb2b | **2.63** | 8.84E-09 | **-8.44** | 3.06E-18 |
| Dr.673 | Dr.673 | BC052753 | rtn1l | **2.55** | 2.98E-06 | **-5.58** | 0.00 |
| Dr.36611 | Dr.36611 | BC091560 | Capon | **5.05** | 4.59E-08 | **-8.82** | 1.14E-07 |
| Dr.77450 | Dr.34965 | BG985584 | id:ibd5033 | **2.04** | 5.91E-21 | **-3.60** | 7.33E-12 |
| Dr.77489 | Dr.31968 | NM_001003874 | flj11749l | 1.48 | 1.74E-06 | **-3.18** | 1.62E-08 |
| Dr.77202 | Dr.2961 | NM_199609 | rdh1l | **2.31** | 7.83E-06 | **-2.95** | 1.11E-28 |
| Dr.76303 | Dr.35771 | AI877794 | psmb1 | 1.28 | 5.39E-06 | -1.51 | 1.16E-08 |
| Dr.46548 | Dr.32774 | CN016739 | im:6909944 | 1.20 | 6.40E-06 | -1.76 | 1.19E-19 |
| [Dr.87232](http://www.ncbi.nlm.nih.gov/UniGene/clust.cgi?ORG=Dr&CID=87232) | - | NM_001003527 | zgc:100865 | **3.56** | 4.46E-07 | **-4.84** | 9.03E-12 |
| Dr.22265 | - | TC301103 | LOC563151: Similar to Prmd14 | 1.91 | 3.36E-06 | **-3.33** | 6.83E-11 |
| Dr.35688 | Dr.31066 | NM_131310 | hsp90b | 1.38 | 4.82E-07 | -1.87 | 1.44E-21 |
| Dr.82165 | Dr.10111 | NM_001004625 | zgc:101772 | **6.82** | 6.37E-13 | **-5.99** | 8.41E-06 |
| Dr.13604 | Dr.13604 | NM_213510 | gyg | 1.21 | 5.57E-06 | -1.28 | 7.37E-07 |
| Dr.83180 | Dr.16740 | CK360551 | Transcribed locus | **3.69** | 5.41E-06 | **-3.11** | 1.68E-06 |
| Dr.77202 | Dr.2961 | NM_199609 | rdh1l | **2.41** | 4.48E-07 | **-4.44** | 6.79E-07 |
| Dr.24982 | Dr.24982 | NM_200327 | zgc:56585 | **6.32** | 1.22E-13 | **-10.56** | 0.00 |
| Dr.85225 | Dr.17450 | NM_200614 | zgc:63663 | **11.75** | 1.21E-20 | **-25.43** | 2.07E-07 |

**Bold** = >2-fold change

**Table S2;** *Anti-correlated regulated genes2*: *ERK1MO down-regulated, ERK2MO up-regulated*

| **Unigene**  Nov 2006 | **Unigene**  old | **Accession** | **Sequence Name(s)** | **ERK1 MO**  **Fold Change** | **ERK1 MO**  **P-value** | **ERK2 MO**  **Fold Change** | **ERK2 MO**  **P-value** |
| --- | --- | --- | --- | --- | --- | --- | --- |
| Dr.81753 | Dr.29741 | NM_205636 | zgc:77312 | **-2.04** | 1.62E-41 | 1.62 | 7.53E-15 |
| Dr.86275 | Dr.17478 | CK869940 | LOC555786: Similar to Npm-A protein | -1.48 | 5.98E-07 | 2.00 | 1.91E-20 |
| Dr.97360 | - | TC281071 | zgc:111826 | -1.75 | 9.09E-18 | 1.99 | 1.32E-20 |
| Dr.120447 | - | TC281322 | LOC798538: Hypothetical protein LOC798538 | -1.86 | 1.20E-09 | **2.68** | 0.00 |
| Dr.81753 | Dr.29741 | NM_205636 | zgc:77312 | **-2.17** | 0.00 | 1.60 | 2.07E-21 |
| Dr.117855 | - | TC296341 | LOC556422: Hypothetical LOC556422 | -1.27 | 6.02E-06 | 1.72 | 2.26E-07 |
| Dr.7638 | Dr.7638 | NM_182967 | calmodulin 3a (phosphorylase kinase, delta) | -1.39 | 1.73E-14 | **2.43** | 1.53E-26 |
| Dr.79220 | Dr.17081 | NM_201065 | surfeit 6-like | -1.43 | 4.72E-17 | **2.09** | 4.80E-17 |
| Dr.86275 | - | TC294580 | LOC555786: Similar to Npm-A protein | -1.52 | 5.86E-10 | 1.78 | 8.46E-12 |
| Dr.9442 | Dr.9442 | NM_205599 | transmembrane protein 32 | -1.37 | 8.57E-06 | 1.22 | 3.61E-13 |
| Dr.85647 | Dr.24770 | NM_001002124 | zgc:86785 | -1.36 | 2.39E-07 | 1.19 | 7.83E-18 |
| [Dr.80074](http://www.ncbi.nlm.nih.gov/UniGene/clust.cgi?ORG=Dr&CID=80074) | - | NM_001020749.1 | pop4 | -1.36 | 1.38E-11 | 1.84 | 8.11E-23 |
| [Dr.90098](http://www.ncbi.nlm.nih.gov/entrez/query.fcgi?db=unigene&term=Dr.37074) | - | TC283522 | si:ch211-154e15.3 | -1.23 | 2.22E-06 | 1.85 | 0.00 |
| Dr.76909 | Dr.38065 | AI444417 | wu:fb38b11 | **-4.83** | 5.16E-08 | **2.88** | 1.35E-36 |
| [Dr.577](http://www.ncbi.nlm.nih.gov/UniGene/clust.cgi?ORG=Dr&CID=577) | - | TC282234 | erythrocyte protein band 4.1-like 4 | **-4.63** | 3.16E-17 | 1.81 | 1.89E-07 |

**Bold** = >2-fold change

**Table S3;** *Commonly down-regulated genes by ERK1or ERK2 knockdown at 30% epiboly*

| **Unigene**  Nov 2006 | **Unigene**  old | **Accession** | **Sequence Name(s)** | **Fold Change**  **ERK1 MO** | **P-value**  **ERK1 MO** | **Fold Change**  **ERK2 MO** | **P-value**  **ERK2 MO** |
| --- | --- | --- | --- | --- | --- | --- | --- |
| Dr.107291 | - | TC269368 | LOC100003783: Hypothetical protein LOC100003783 | -1.28 | 1.79E-08 | **-1.41** | 1.35E-30 |
| [Dr.10261](http://www.ncbi.nlm.nih.gov/UniGene/clust.cgi?ORG=Dr&CID=10261) | - | [NM_213223](http://www.ncbi.nlm.nih.gov/entrez/viewer.fcgi?val=NM_213223) | zgc:85717 | -1.30 | 9.09E-06 | **-3.89** | 6.03E-06 |
| [Dr.14471](http://www.ncbi.nlm.nih.gov/UniGene/clust.cgi?ORG=Dr&CID=14471) | - | TC271332 | zgc:100838 | -2.70 | 2.07E-06 | **-2.70** | 1.08E-08 |
| Dr.14471 | Dr.14471 | NM_001002753 | zgc:100838 | **-2.52** | 8.14E-07 | -2.20 | 2.96E-08 |
| Dr.16023 | Dr.16023 | NM_001003414 | c6orf194l | **-1.38** | 1.97E-07 | -1.21 | 1.53E-10 |
| Dr.16839 | Dr.5512 | NM_213486 | ube2n | -1.40 | 1.73E-14 | **-1.85** | 2.93E-11 |
| Dr.1778 | Dr.1778 | NM_212691 | sdcbp | -1.27 | 9.91E-06 | **-1.92** | 1.54E-26 |
| Dr.193 | Dr.193 | NM_131406 | rara2a | -1.39 | 6.54E-09 | **-3.34** | 2.83E-20 |
| Dr.20345 | Dr.20345 | NM_001002041 | flj12949l | -1.71 | 1.75E-07 | **-2.25** | 3.49E-09 |
| Dr.24323 | Dr.24323 | NM_201094 | zgc:56608 | -1.22 | 9.39E-07 | **-1.43** | 1.56E-06 |
| Dr.25046 | Dr.25046 | NM_001003542 | zgc:100832 | -1.70 | 1.25E-08 | **-5.82** | 7.18E-21 |
| Dr.2532 | Dr.2532 | NM_131855 | prkci | -1.27 | 4.03E-06 | **-1.68** | 7.01E-45 |
| Dr.2648 | Dr.2648 | NM_200630 | arpp19 | -1.88 | 5.75E-23 | **-4.51** | 0.00 |
| Dr.28410 | Dr.1434 | NM_131156 | krt5 | -1.96 | 3.99E-12 | **-6.96** | 0.00 |
| Dr.28410 | Dr.1434 | NM_131156 | krt5 | -1.52 | 9.14E-06 | **-8.88** | 0.00 |
| Dr.28410 | Dr.1434 | BC063955 | krt5 | -1.73 | 3.11E-27 | **-8.26** | 0.00 |
| Dr.28410 | Dr.35045 | AI618852 | hm:zehn2354 | -1.93 | 5.65E-06 | **-5.96** | 6.66E-08 |
| Dr.28410 | - | NM_131156 | krt5 | -1.83 | 2.93E-16 | **-12.63** | 1.79E-22 |
| [Dr.2860](http://www.ncbi.nlm.nih.gov/UniGene/clust.cgi?ORG=Dr&CID=2860) | - | [NM_131401](http://www.ncbi.nlm.nih.gov/entrez/viewer.fcgi?val=NM_131401) | hspa8 | -1.31 | 9.91E-08 | **-2.55** | 4.92E-26 |
| Dr.30945 | Dr.30945 | NM_131187 | ck2b | -1.24 | 3.89E-07 | **-2.70** | 0.00 |
| Dr.31276 | Dr.31276 | CO936527 | LOC793723: Hypothetical protein LOC793723 | -1.66 | 3.69E-18 | **-1.94** | 2.09E-28 |
| Dr.31375 | Dr.31375 | NM_001002479 | zgc:92873 | **-2.57** | 1.37E-06 | -1.72 | 3.20E-07 |
| Dr.32146 | Dr.32146 | NM_001003873 | rbbp6l | -1.74 | 5.26E-16 | **-5.63** | 4.95E-21 |
| Dr.32370 | Dr.47535 | AW594843 | wu:fk24g11 | -1.34 | 1.56E-11 | **-8.72** | 1.56E-18 |
| Dr.33171 | Dr.5477 | NM_194376 | ptma | **-1.55** | 1.66E-06 | -1.53 | 1.11E-06 |
| [Dr.33010](http://www.ncbi.nlm.nih.gov/entrez/query.fcgi?db=unigene&term=Dr.33677) | [Dr.38914](http://www.ncbi.nlm.nih.gov/UniGene/clust.cgi?ORG=Dr&CID=38914) | NM_001082849.1 | [MGC158377: Hypothetical LOC566409](http://www.ncbi.nlm.nih.gov/entrez/query.fcgi?db=gene&cmd=Retrieve&dopt=Graphics&list_uids=566409) | -2.21 | 5.17E-10 | **-3.92** | 2.91E-12 |
| Dr.34080 | Dr.12362 | NM_001003514 | cutl1 | -1.32 | 6.16E-11 | **-1.59** | 1.50E-26 |
| Dr.34080 | Dr.12362 | NM_001003514 | cutl1 | -1.29 | 9.53E-09 | **-1.48** | 2.97E-07 |
| [Dr.34325](http://www.ncbi.nlm.nih.gov/UniGene/clust.cgi?ORG=Dr&CID=34325) | - | TC274674 | zgc:101705 | -1.37 | 1.77E-07 | **-2.27** | 1.13E-16 |
| Dr.34325 | Dr.34325 | NM_001006070 | zgc:101705 | -1.35 | 4.31E-09 | **-2.30** | 1.40E-15 |
| Dr.34325 | Dr.34325 | NM_001006070 | zgc:101705 | -1.49 | 1.54E-14 | **-2.27** | 4.06E-17 |
| Dr.35845 | Dr.20272 | NM_001003875 | snrp70 | -1.48 | 2.00E-08 | **-2.30** | 4.75E-21 |
| Dr.36960 | Dr.28340 | NM_001005583 | zgc:91861 | -1.35 | 4.86E-07 | **-2.45** | 0.00E+00 |
| Dr.38353 | Dr.1040 | NM_001012304 | rbm39a | -1.33 | 1.10E-10 | **-2.18** | 0.00 |
| Dr.40045 | Dr.30007 | NM_001006045 | zgc:103418 | -1.24 | 1.15E-06 | **-1.47** | 1.60E-38 |
| Dr.43242 | Dr.29962 | CK688091 | im:7151115 | -1.46 | 6.29E-06 | **-4.61** | 1.36E-08 |
| [Dr.45492](http://www.ncbi.nlm.nih.gov/UniGene/clust.cgi?ORG=Dr&CID=45492) | - | TC269062 | zgc:110689 | -1.39 | 3.74E-15 | **-33.15** | 0.00 |
| Dr.455 | Dr.455 | NM_207048 | ccnf | -1.41 | 6.92E-10 | **-4.26** | 2.23E-28 |
| Dr.4763 | Dr.4763 | NM_131336 | sox11a | -1.33 | 2.89E-09 | **-4.22** | 7.25E-10 |
| Dr.48550 | Dr.30592 | NM_001001592 | hnrpu | -1.33 | 5.25E-06 | **-2.46** | 0.00 |
| Dr.6046 | Dr.6046 | NM_205654 | zgc:77262 | -1.55 | 6.11E-06 | **-1.71** | 3.17E-07 |
| [Dr.75873](http://www.ncbi.nlm.nih.gov/entrez/query.fcgi?db=unigene&term=Dr.696) | - | NM_001045488.1 | Type IV antifreeze protein precursor (LOC568707) | -1.77 | 2.11E-06 | **-6.21** | 0.00 |
| Dr.74545 | Dr.18270 | CK352920 | Wu:fi20e01 | -2.04 | 8.10E-09 | **-2.97** | 2.61E-17 |
| Dr.75119 | Dr.1360 | NM_200852 | rps9 | -1.32 | 5.99E-06 | **-1.54** | 1.25E-13 |
| Dr.75131 | Dr.3347 | NM_213002 | fbl | -1.22 | 7.17E-06 | **-1.35** | 7.27E-11 |
| Dr.75235 | Dr.18971 | BC080208 | zgc:66241 | **-1.43** | 8.51E-14 | -1.32 | 7.61E-07 |
| Dr.75271 | Dr.15502 | NM_199754 | zgc:77455 | -1.34 | 3.58E-10 | **-3.99** | 6.61E-39 |
| Dr.115097 | Dr.6204 | CN022011 | LOC794591: Hypothetical protein LOC794591 | -1.75 | 5.20E-08 | **-2.53** | 9.85E-28 |
| Dr.75499 | Dr.4845 | NM_212614 | mycn | -1.71 | 4.53E-09 | **-5.37** | 2.50E-14 |
| Dr.75559 | Dr.5122 | NM_212645 | zgc:77366 | -2.41 | 8.10E-11 | **-5.40** | 2.42E-11 |
| Dr.75583 | Dr.32512 | AW077362 | wu:fj34c04 | -1.29 | 9.27E-09 | **-2.25** | 5.13E-33 |
| Dr.75583 | - | [NM_001001590](http://www.ncbi.nlm.nih.gov/entrez/viewer.fcgi?val=NM_001001590) | rpl3 | -1.28 | 1.21E-06 | **-1.77** | 8.57E-16 |
| [Dr.75615](http://www.ncbi.nlm.nih.gov/UniGene/clust.cgi?ORG=Dr&CID=75615) | - | TC290861 | eef1d: Elongation factor-1, delta | -1.26 | 8.84E-07 | **-2.83** | 0.00 |
| Dr.75654 | Dr.664 | NM_200185 | tuba8l4 | -1.23 | 4.68E-06 | **-2.25** | 0.00 |
| Dr.75798 | Dr.5735 | NM_131123 | hoxc6a | -1.53 | 6.03E-07 | **-3.63** | 0.00 |
| Dr.76013 | Dr.39192 | NM_131763 | cldnb | -1.52 | 6.51E-18 | **-2.23** | 7.24E-16 |
| Dr.76043 | Dr.33885 | NM_001003432 | zgc:92872 | -1.50 | 9.12E-10 | **-2.12** | 3.07E-07 |
| Dr.76046 | Dr.49998 | NM_131668 | atp1b1a | -1.54 | 5.58E-08 | **-7.49** | 0.00 |
| Dr.76062 | Dr.31052 | AI331035 | zgc:92371 | -1.26 | 2.84E-07 | **-1.92** | 1.36E-11 |
| Dr.76099 | Dr.3502 | NM_001002040 | cdkn1c | -1.25 | 1.40E-06 | **-2.62** | 7.28E-06 |
| Dr.76170 | Dr.5630 | NM_201099 | zgc:55429 | -1.54 | 2.68E-06 | **-2.04** | 2.07E-07 |
| Dr.76245 | Dr.3873 | NM_213020 | zgc:73347 | -1.78 | 2.12E-12 | **-1.89** | 1.02E-34 |
| [Dr.76650](http://www.ncbi.nlm.nih.gov/UniGene/clust.cgi?ORG=Dr&CID=76650) | - | TC269127 | zgc:110619 | -1.79 | 5.08E-16 | **-2.94** | 0.00 |
| Dr.76826 | Dr.4105 | NM_213219 | akap8l | -1.36 | 2.96E-07 | **-2.55** | 0.00 |
| Dr.76956 | Dr.4816 | BC081665 | PNN | -1.50 | 1.25E-08 | **-1.84** | 1.06E-08 |
| Dr.76956 | Dr.4816 | BC056140 | PNN | -1.52 | 1.02E-09 | **-1.86** | 4.32E-08 |
| Dr.77086 | Dr.3545 | NM_001002422 | zgc:92664 | -1.28 | 1.16E-06 | **-1.65** | 1.65E-06 |
| Dr.77098 | Dr.25854 | NM_212780 | zgc:77093 | -1.27 | 3.20E-08 | **-1.78** | 6.41E-21 |
| [Dr.77277](http://www.ncbi.nlm.nih.gov/UniGene/clust.cgi?ORG=Dr&CID=77277) | - | AI544617 | lmo1 | -3.00 | 3.91E-09 | **-3.59** | 4.62E-08 |
| [Dr.77771](http://www.ncbi.nlm.nih.gov/UniGene/clust.cgi?ORG=Dr&CID=77771) | - | [NM_001075103](http://www.ncbi.nlm.nih.gov/entrez/viewer.fcgi?val=NM_001075103) | si:dkey-252h13.6 | -1.36 | 4.16E-06 | **-3.83** | 1.75E-08 |
| [Dr.77771](http://www.ncbi.nlm.nih.gov/UniGene/clust.cgi?ORG=Dr&CID=77771) | - | [NM_001075103](http://www.ncbi.nlm.nih.gov/entrez/viewer.fcgi?val=NM_001075103) | si:dkey-252h13.6 | -1.53 | 2.95E-06 | **-5.36** | 2.99E-16 |
| Dr.78467 | Dr.9366 | NM_001004550 | zgc:92505 | -1.38 | 1.35E-07 | **-2.58** | 1.60E-22 |
| Dr.79556 | Dr.14044 | NM_001002691 | zgc:92386 | -1.55 | 4.11E-12 | **-2.94** | 0.00 |
| Dr.79556 | Dr.14044 | NM_001002691 | gpr137bb | -1.50 | 3.96E-20 | **-2.59** | 2.65E-12 |
| [Dr.79763](http://www.ncbi.nlm.nih.gov/UniGene/clust.cgi?ORG=Dr&CID=79763) | - | NM_200879 | slc30a1 | -1.42 | 2.70E-07 | **-3.76** | 0.00 |
| Dr.79763 | Dr.12303 | NM_200879 | slc30a1 | -1.41 | 8.29E-07 | **-3.73** | 0.00 |
| Dr.80222 | Dr.6582 | NM_205716 | casc3 | -1.27 | 2.04E-07 | **-1.40** | 5.18E-06 |
| Dr.80715 | Dr.20972 | NM_199887 | msto1 | -1.80 | 1.13E-06 | **-2.89** | 0.00 |
| Dr.81466 | Dr.13338 | NM_174861 | gbx1 | -3.17 | 1.03E-15 | **-4.13** | 2.00E-08 |
| Dr.81793 | Dr.9681 | BC090683 | zgc:113197 | -1.55 | 1.04E-22 | **-1.66** | 1.82E-22 |
| Dr.8283 | Dr.8283 | BC050155 | mibp | -1.76 | 7.91E-06 | **-4.31** | 1.30E-08 |
| Dr.83843 | Dr.16714 | NM_001004591 | zgc:92155 | -1.76 | 8.31E-25 | **-1.30** | 3.24E-11 |
| Dr.84135 | Dr.18694 | AY427670 | rgs12 | -1.59 | 7.00E-06 | **-5.12** | 2.57E-07 |
| Dr.84258 | Dr.15055 | NM_131882 | cxcr4a | -1.45 | 2.75E-12 | **-2.98** | 7.74E-06 |
| Dr.8506 | Dr.8506 | NM_176859 | tfap2a | -1.53 | 8.09E-10 | **-2.95** | 2.39E-18 |
| Dr.80032 | - | TC269162 | LOC562319: Hypothetical LOC562319 | -1.40 | 7.39E-06 | **-1.41** | 1.12E-13 |
| Dr.76424 | - | TC271329 | ezh2: Enhancer of zeste homolog 2 (Drosophila) | -1.31 | 9.48E-09 | **-1.63** | 2.26E-15 |
| Dr.106900 | - | TC281884 | zmpste24: Zinc metallopeptidase, STE24 homolog | -1.23 | 1.49E-06 | **-2.23** | 2.79E-07 |
| Dr.75615 | - | TC290859 | eef1d: Elongation factor-1, delta | -1.45 | 2.60E-13 | **-1.98** | 1.67E-40 |
| Dr.115936 | - | TC291483 | hp: Haptoglobin | -1.61 | 1.26E-12 | **-6.93** | 2.26E-15 |
| Dr.105771 | - | TC291958 | LOC100007704: Similar to Slc7a8-prov protein | -1.39 | 6.76E-12 | **-9.57** | 3.84E-18 |
| - | - | TC296381 | weakly similar to DKK3_HUMAN (Q9UBP4) | -1.70 | 3.25E-20 | **-2.70** | 0.00 |

**Bold** = >2-fold change

***Table S4;*** *Commonly up-regulated genes* by ERK1or ERK2 knockdown at 30% epiboly

| **Unigene**  Nov 2006 | **Unigene**  old | **Accession** | **Sequence Name(s)** | **Fold Change**  **ERK1 MO** | **P-value**  **ERK1 MO** | **Fold Change**  **ERK2 MO** | **P-value**  **ERK2 MO** |
| --- | --- | --- | --- | --- | --- | --- | --- |
| Dr.5450 | Dr.5450 | AB055682 | snx1 | 1.33 | 1.74E-10 | **3.32** | 1.40E-45 |
| Dr.15775 | Dr.15775 | NM_201001 | arl2bp | 1.78 | 1.15E-06 | **2.55** | 2.80E-45 |
| Dr.10390 | Dr.10390 | NM_201509 | arpc5b | 1.59 | 3.26E-18 | **3.17** | 0.00E+00 |
| Dr.75686 | Dr.4174 | NM_200041 | atp5c1 | 1.60 | 3.33E-24 | **3.08** | 1.10E-25 |
| Dr.76078 | Dr.4272 | NM_199968 | atp5d | 1.29 | 1.43E-06 | **1.90** | 8.76E-07 |
| Dr.5571 | Dr.7822 | NM_213023 | atp6ap2 | 1.63 | 8.00E-06 | **2.00** | 1.40E-24 |
| Dr.5571 | Dr.7822 | NM_213023 | atp6ap2 | 1.64 | 9.32E-07 | **2.03** | 1.66E-29 |
| Dr.77332 | Dr.2032 | NM_199620 | atp6v0d1 | 2.43 | 4.22E-39 | **5.45** | 6.02E-29 |
| Dr.14783 | Dr.14783 | NM_201322 | atp6v1c1 | 1.51 | 1.55E-12 | **2.14** | 2.37E-08 |
| Dr.7421 | Dr.7421 | NM_173270 | atp6v1h | 1.47 | 3.80E-06 | **2.89** | 8.38E-15 |
| Dr.4575 | Dr.4575 | BC045974 | LOC559713: Hypothetical LOC559713 | 1.41 | 1.96E-07 | **3.11** | 0.00E+00 |
| Dr.88787 | Dr.15607 | BC049494 | LOC559713: Hypothetical LOC559713 | **1.86** | 5.25E-07 | 1.86 | 5.40E-12 |
| Dr.76330 | Dr.25238 | BC078346 | tom1 | 2.72 | 7.50E-23 | **3.57** | 0.00 |
| Dr.82699 | Dr.12860 | BC090274 | zgc:112943 | 1.34 | 3.03E-08 | **4.73** | 0.00 |
| Dr.83494 | Dr.14518 | BC090293 | zgc:110788 | 2.44 | 4.69E-27 | **4.19** | 0.00 |
| Dr.86302 | Dr.17236 | BC090312 | zgc:113297 | 2.00 | 1.72E-07 | **3.76** | 9.81E-16 |
| Dr.82441 | Dr.11508 | NM_131807 | bcl2l | 1.41 | 1.11E-12 | **1.97** | 6.13E-17 |
| Dr.79316 | Dr.10370 | BE605779 | cul5 | 1.46 | 6.57E-08 | **3.64** | 0.00 |
| Dr.14671 | Dr.14671 | NM_194397 | birc5a | 1.32 | 6.98E-06 | **2.26** | 3.27E-06 |
| Dr.80585 | Dr.42837 | BQ260954 | LOC553480 | 1.71 | 1.30E-07 | **3.14** | 0.00 |
| Dr.78272 | Dr.7102 | NM_200008 | c20orf149l | 1.35 | 1.49E-12 | **3.98** | 9.65E-13 |
| Dr.75663 | Dr.26975 | NM_180964 | cldnd | 2.10 | 0.00 | **4.86** | 2.65E-41 |
| [Dr.86063](http://www.ncbi.nlm.nih.gov/UniGene/clust.cgi?ORG=Dr&CID=86063) | Dr.30042 | [NM_001017874](http://www.ncbi.nlm.nih.gov/entrez/viewer.fcgi?val=NM_001017874) | zgc:110674 | 1.34 | 8.67E-07 | **1.69** | 8.71E-06 |
| Dr.14821 | Dr.14821 | CN017675 | LOC792919 | **1.87** | 3.24E-09 | 1.42 | 7.20E-15 |
| Dr.43242 | Dr.14123 | CN836761 | zgc:112028 | 1.52 | 1.01E-07 | **2.25** | 6.56E-06 |
| Dr.84525 | Dr.13849 | CO350434 | zgc:153251 | 1.35 | 5.50E-10 | **3.68** | 0.00E+00 |
| Dr.87484 | Dr.31273 | [NM_001017735](http://www.ncbi.nlm.nih.gov/entrez/viewer.fcgi?val=NM_001017735) | zgc:112142 | 1.30 | 4.13E-07 | **2.07** | 4.50E-31 |
| Dr.84881 | Dr.15000 | CO815744 | lypla1: Lysophospholipase I | 1.88 | 7.49E-06 | **5.04** | 2.12E-20 |
| Dr.8425 | Dr.8425 | NM_131512 | coil | 1.69 | 6.60E-26 | **4.34** | 6.45E-20 |
| Dr.75878 | Dr.11635 | BC066452 | cox15 | 1.43 | 1.18E-07 | **2.40** | 4.09E-19 |
| Dr.75878 | Dr.11635 | NM_201206 | cox15 | 1.39 | 4.71E-13 | **2.50** | 0.00 |
| Dr.79316 | Dr.6630 | NM_212706 | cul5 | 1.64 | 8.03E-07 | **2.46** | 2.43E-07 |
| Dr.21365 | Dr.8207 | NM_131810 | cx44.2 | 1.34 | 1.72E-07 | **2.85** | 0.00 |
| Dr.79878 | Dr.9443 | NM_205757 | dab2 | 1.47 | 1.51E-06 | **2.80** | 0.00E+00 |
| Dr.26555 | Dr.28249 | NM_201045 | ddx41 | 1.83 | 2.01E-11 | **2.56** | 4.18E-27 |
| Dr.80276 | Dr.15729 | NM_201313 | dnaja3a | 1.30 | 1.25E-08 | **2.27** | 3.13E-24 |
| Dr.9667 | Dr.9667 | NM_198821 | dnajb11 | 1.40 | 5.15E-13 | **2.05** | 0.00 |
| Dr.76129 | Dr.25678 | NM_214723 | eno3 | 1.27 | 5.28E-07 | **1.69** | 2.41E-20 |
| [Dr.14203](http://www.ncbi.nlm.nih.gov/UniGene/clust.cgi?ORG=Dr&CID=14203) | - | [NM_001017863](http://www.ncbi.nlm.nih.gov/entrez/viewer.fcgi?val=NM_001017863) | zgc:110700 | 1.41 | 3.61E-12 | **3.57** | 6.96E-20 |
| [Dr.78119](http://www.ncbi.nlm.nih.gov/entrez/query.fcgi?db=unigene&term=Dr.30111) | - | NM_001076738.1 | zgc:153556 | **2.23** | 4.52E-06 | 2.08 | 5.77E-38 |
| Dr.79837 | - | [NM_001080187](http://www.ncbi.nlm.nih.gov/entrez/viewer.fcgi?db=nucleotide&val=68363813) | ptpmt1 | 1.92 | 1.85E-13 | **3.51** | 5.38E-13 |
| [Dr.40150](http://www.ncbi.nlm.nih.gov/UniGene/clust.cgi?ORG=Dr&CID=40150) | - | [BC091955](http://www.ncbi.nlm.nih.gov/entrez/query.fcgi?cmd=Search&db=Nucleotide&doptcmdl=GenBank&term=BC091955) | im:6901326 | 1.27 | 3.12E-06 | **3.11** | 0.00 |
| [Dr.78581](http://www.ncbi.nlm.nih.gov/UniGene/clust.cgi?ORG=Dr&CID=78581) | - | [NM_001076561](http://www.ncbi.nlm.nih.gov/entrez/viewer.fcgi?val=NM_001076561) | zgc:153688 | 1.43 | 7.08E-07 | **2.02** | 6.26E-11 |
| Dr.98360 | - | [XM_001334149](http://www.ncbi.nlm.nih.gov/entrez/query.fcgi?cmd=Search&db=Nucleotide&doptcmdl=GenBank&term=BX539345) | LOC797867: Hypothetical protein LOC797867 | 1.35 | 4.04E-06 | **1.73** | 4.74E-07 |
| - | - | NW_001510520 | chromosome 10 genomic contig | 1.92 | 1.49E-08 | **5.54** | 0.00 |
| - | - | NW_001510520 | chromosome 10 genomic contig | 1.75 | 3.74E-13 | **5.79** | 0.00E+00 |
| Dr.81293 | Dr.19643 | NM_001005292 | fabgl | 1.76 | 3.87E-19 | **3.68** | 0.00 |
| Dr.79341 | Dr.15074 | [CK682305](http://www.ncbi.nlm.nih.gov/entrez/query.fcgi?cmd=Search&db=Nucleotide&doptcmdl=GenBank&term=CK682305) | im:7144322 | 1.48 | 1.55E-09 | **2.03** | 2.47E-08 |
| Dr.76244 | Dr.5598 | NM_200944 | itgb4bp4 | 1.25 | 5.99E-06 | **1.65** | 4.09E-34 |
| Dr.6104 | Dr.6104 | NM_199480 | lnp | 1.62 | 7.44E-13 | **2.47** | 1.61E-32 |
| Dr.6104 | Dr.6104 | NM_199480 | lnp | 1.58 | 7.08E-12 | **2.73** | 3.18E-11 |
| Dr.86533 | Dr.28396 | BC057252 | LOC402869 | 1.65 | 5.04E-06 | **2.63** | 3.23E-21 |
| Dr.87071 | Dr.19969 | BC067706 | LOC407678 | **2.32** | 2.51E-17 | 2.20 | 9.87E-14 |
| Dr.6972 | Dr.6972 | NM_201306 | lrpap1 | 1.32 | 1.79E-06 | **3.30** | 5.96E-39 |
| Dr.12572 | Dr.12572 | NM_205580 | mos | 3.45 | 6.58E-27 | **10.35** | 1.04E-36 |
| Dr.12572 | Dr.12572 | NM_205580 | mos | 3.22 | 8.21E-27 | **7.57** | 0.00 |
| Dr.7209 | Dr.7209 | BC075738 | ndpkz6 | **1.60** | 1.63E-10 | 1.42 | 1.49E-11 |
| [Dr.15832](http://www.ncbi.nlm.nih.gov/UniGene/clust.cgi?ORG=Dr&CID=15832) | - | [NM_201345](http://www.ncbi.nlm.nih.gov/entrez/viewer.fcgi?val=NM_201345) | pafah1b1a | 1.32 | 2.44E-09 | **2.37** | 4.49E-28 |
| Dr.78299 | Dr.12015 | NM_199805 | phf17 | 1.69 | 1.45E-07 | **2.36** | 3.21E-06 |
| Dr.15883 | Dr.15883 | NM_194412 | ppp2r5e1 | 1.30 | 4.99E-09 | **1.97** | 9.19E-27 |
| Dr.84921 | Dr.15087 | NM_200978 | prkcb1 | 1.75 | 7.61E-19 | **1.65** | 6.45E-22 |
| Dr.79936 | Dr.10290 | NM_205754 | psmd10 | 1.57 | 1.02E-08 | **3.37** | 0.00 |
| Dr.86325 | Dr.30366 | NM_212950 | pth1 | 2.20 | 2.06E-07 | **6.40** | 0.00 |
| Dr.84946 | Dr.15361 | NM_205719 | rab22a | 1.30 | 1.40E-08 | **1.29** | 5.66E-15 |
| Dr.84946 | Dr.15361 | NM_205719 | rab22a | 1.32 | 1.96E-06 | **1.41** | 2.10E-11 |
| Dr.76462 | Dr.2741 | NM_212595 | rbb4 | 1.25 | 3.12E-07 | **1.41** | 5.45E-13 |
| Dr.76324 | Dr.36340 | CN500419 | sb:cb1095 | 1.42 | 8.53E-06 | **1.59** | 7.15E-06 |
| Dr.3854 | Dr.3854 | NM_199777 | sec23b | 1.54 | 1.31E-24 | **3.05** | 1.01E-27 |
| Dr.83056 | Dr.12218 | NM_200181 | sh3gl3 | 1.38 | 2.09E-08 | **1.87** | 2.06E-11 |
| Dr.1823 | Dr.1823 | NM_001006019 | si:ch211-150c22.2 | 1.46 | 3.52E-14 | **3.70** | 2.30E-14 |
| Dr.32625 | Dr.8332 | NM_212566 | stka | 1.44 | 7.14E-16 | **2.05** | 5.20E-06 |
| - | - | [NW_001511282](http://www.ncbi.nlm.nih.gov/entrez/viewer.fcgi?db=nucleotide&val=68438792) | chromosome 15 genomic contig | 1.87 | 3.00E-15 | **3.61** | 0.00 |
| [Dr.77586](http://www.ncbi.nlm.nih.gov/UniGene/clust.cgi?ORG=Dr&CID=77586) | - | [CF924885](http://www.ncbi.nlm.nih.gov/entrez/query.fcgi?cmd=Search&db=Nucleotide&doptcmdl=GenBank&term=CF924885) | chac1 | 1.45 | 2.18E-08 | **4.26** | 0.00E+00 |
| [Dr.48748](http://www.ncbi.nlm.nih.gov/UniGene/clust.cgi?ORG=Dr&CID=48748) | - | [NM_001025515](http://www.ncbi.nlm.nih.gov/entrez/viewer.fcgi?val=NM_001025515) | si:ch211-150c22.3 | 1.37 | 5.69E-06 | **3.40** | 0.00 |
| [Dr.26907](http://www.ncbi.nlm.nih.gov/UniGene/clust.cgi?ORG=Dr&CID=26907) | - | [NM_212795](http://www.ncbi.nlm.nih.gov/entrez/viewer.fcgi?val=NM_212795) | dnd | 1.37 | 1.34E-06 | **3.31** | 3.96E-14 |
| [Dr.11214](http://www.ncbi.nlm.nih.gov/UniGene/clust.cgi?ORG=Dr&CID=11214) | - | [NM_200787](http://www.ncbi.nlm.nih.gov/entrez/viewer.fcgi?val=NM_200787) | gnao1 | 1.32 | 6.11E-10 | **2.91** | 0.00E+00 |
| [Dr.78131](http://www.ncbi.nlm.nih.gov/UniGene/clust.cgi?ORG=Dr&CID=78131) | - | TC270839 | lin7b | 1.88 | 4.03E-07 | **2.43** | 5.46E-07 |
| Dr.79837 | - | [NM_001080187](http://www.ncbi.nlm.nih.gov/entrez/viewer.fcgi?db=protein&val=68363814) | ptpmt1 | 2.04 | 5.92E-15 | **2.94** | 8.75E-08 |
| [Dr.39121](http://www.ncbi.nlm.nih.gov/UniGene/clust.cgi?ORG=Dr&CID=39121) | [Dr.47510](http://www.ncbi.nlm.nih.gov/entrez/query.fcgi?db=unigene&term=Dr.47510) | [NM_001045312](http://www.ncbi.nlm.nih.gov/entrez/viewer.fcgi?val=NM_001045312) | zgc:136689 | 1.46 | 1.08E-07 | **2.12** | 0.00E+00 |
| [Dr.84881](http://www.ncbi.nlm.nih.gov/UniGene/clust.cgi?ORG=Dr&CID=84881) | - | [NM_001017616](http://www.ncbi.nlm.nih.gov/entrez/viewer.fcgi?val=NM_001017616) | lypla1 | 2.00 | 2.97E-11 | **4.91** | 3.12E-35 |
| Dr.30700 | - | TC273038 | trdmt1: TRNA aspartic acid methyltransferase 1 | 1.40 | 3.33E-07 | **1.75** | 7.37E-27 |
| [Dr.83342](http://www.ncbi.nlm.nih.gov/UniGene/clust.cgi?ORG=Dr&CID=83342) | - | [NM_001020611](http://www.ncbi.nlm.nih.gov/entrez/viewer.fcgi?val=NM_001020611) | zgc:110614 | 1.31 | 8.87E-06 | **1.39** | 1.40E-09 |
| Dr.121252 | - | XP_689621 | LOC561124 | 1.59 | 1.41E-20 | **3.28** | 1.86E-12 |
| [Dr.77997](http://www.ncbi.nlm.nih.gov/UniGene/clust.cgi?ORG=Dr&CID=77997) | - | [WZ14634.1](http://www.ensembl.org/Danio_rerio/r?d=WZ;ID=14634.1) | senp3a | 1.37 | 6.16E-10 | **2.18** | 5.71E-19 |
| Dr.32734 | - | [NM_212605](http://www.ncbi.nlm.nih.gov/entrez/viewer.fcgi?val=NM_212605) | ywhae1 | 1.40 | 1.83E-06 | **2.45** | 2.54E-21 |
| Dr.116491 | - | [XM_704085](http://www.ncbi.nlm.nih.gov/entrez/viewer.fcgi?db=nucleotide&val=68366605) | LOC566620: hypothetical LOC566620 | 1.65 | 3.08E-06 | **2.22** | 2.94E-17 |
| [Dr.57547](http://www.ncbi.nlm.nih.gov/UniGene/clust.cgi?ORG=Dr&CID=57547) | - | [NM_001024398](http://www.ncbi.nlm.nih.gov/entrez/viewer.fcgi?val=NM_001024398) | zgc:112234 | 1.41 | 1.37E-10 | **4.22** | 5.78E-18 |
| [Dr.5314](http://www.ncbi.nlm.nih.gov/UniGene/clust.cgi?ORG=Dr&CID=5314) | - | [NM_001003762](http://www.ncbi.nlm.nih.gov/entrez/viewer.fcgi?val=NM_001003762) | arpc4l | 1.48 | 8.95E-06 | **1.64** | 7.12E-15 |
| Dr.107342 | - | TC280484 | si:dkey-63j1.8 | 1.43 | 8.20E-06 | **1.89** | 3.24E-07 |
| Dr.79005 | - | NM_198372.1 | Family with sequence similarity 107- B (fam107b) | 1.38 | 2.70E-07 | **7.92** | 0.00 |
| [Dr.15144](http://www.ncbi.nlm.nih.gov/UniGene/clust.cgi?ORG=Dr&CID=15144) | - | [NM_200676](http://www.ncbi.nlm.nih.gov/entrez/viewer.fcgi?val=NM_200676) | zgc:66030 | 1.74 | 5.37E-07 | **4.44** | 1.86E-25 |
| [Dr.80595](http://www.ncbi.nlm.nih.gov/UniGene/clust.cgi?ORG=Dr&CID=80595) | - | [NM_200343](http://www.ncbi.nlm.nih.gov/entrez/viewer.fcgi?db=nucleotide&val=41054402) | pias4l | 1.68 | 1.02E-11 | **2.43** | 9.05E-08 |
| [Dr.75734](http://www.ncbi.nlm.nih.gov/UniGene/clust.cgi?ORG=Dr&CID=75734) | - | [NM_131696](http://www.ncbi.nlm.nih.gov/entrez/viewer.fcgi?val=NM_131696) | zp3b | 1.58 | 6.09E-19 | **1.91** | 9.87E-13 |
| Dr.113413 | - | [CT670887](http://www.ensembl.org/Danio_rerio/r?d=EMBL;ID=CT670887) | LOC100006913: Similar to RNA binding motif protein 15B | 1.33 | 4.30E-06 | **1.76** | 5.08E-25 |
| [Dr.123292](http://www.ncbi.nlm.nih.gov/UniGene/clust.cgi?ORG=Dr&CID=15033) | - | BM037355 | Transcribed locus | 1.77 | 4.86E-31 | **3.51** | 0.00E+00 |
| [Dr.15734](http://www.ncbi.nlm.nih.gov/UniGene/clust.cgi?ORG=Dr&CID=15734) | - | [NM_001077292](http://www.ncbi.nlm.nih.gov/entrez/viewer.fcgi?db=nucleotide&val=116267976) | zgc:153652 | 1.68 | 5.38E-13 | **2.77** | 1.26E-09 |
| Dr.101975 | - | TC282984 | zgc:158395 | 1.60 | 7.86E-18 | **2.75** | 8.03E-13 |
| Dr.84533 | - | [BI867891](http://www.ensembl.org/Danio_rerio/r?d=EMBL;ID=BI867891) | LOC557562: Similar to zinc finger protein | 1.39 | 1.85E-08 | **2.75** | 0.00 |
| [Dr.48553](http://www.ncbi.nlm.nih.gov/UniGene/clust.cgi?ORG=Dr&CID=48553) | - | [NM_001033101](http://www.ncbi.nlm.nih.gov/entrez/viewer.fcgi?val=NM_001033101) | zgc:111974 | 2.22 | 7.27E-10 | **2.03** | 1.17E-14 |
| [Dr.76050](http://www.ncbi.nlm.nih.gov/UniGene/clust.cgi?ORG=Dr&CID=76050) | - | TC290216 | atp5a1 | **1.47** | 1.53E-10 | 1.29 | 1.54E-09 |
| - | - | [XP_687906](http://www.ncbi.nlm.nih.gov/entrez/viewer.fcgi?db=protein&val=68356026) | PREDICTED: similar to H01CJC protein isoform 1 | 1.50 | 5.21E-08 | **1.62** | 5.63E-13 |
| Dr.76765 | - | TC291935 | zgc:153980 | 1.28 | 2.44E-07 | **2.63** | 0.00 |
| [Dr.78284](http://www.ncbi.nlm.nih.gov/UniGene/clust.cgi?ORG=Dr&CID=78284) | - | TC292195 | zgc:109987 | 2.24 | 1.94E-08 | **4.85** | 1.03E-33 |
| [Dr.82922](http://www.ncbi.nlm.nih.gov/UniGene/clust.cgi?ORG=Dr&CID=82922) | - | [NM_205713](http://www.ncbi.nlm.nih.gov/entrez/viewer.fcgi?val=NM_205713) | unc119.2 | 1.81 | 1.76E-19 | **3.00** | 0.00 |
| [Dr.77560](http://www.ncbi.nlm.nih.gov/UniGene/clust.cgi?ORG=Dr&CID=77560) | - | [NM_001030203](http://www.ncbi.nlm.nih.gov/entrez/viewer.fcgi?val=NM_001030203) | zgc:114109 | 1.31 | 3.26E-06 | **1.73** | 7.75E-12 |
| [Dr.80160](http://www.ncbi.nlm.nih.gov/UniGene/clust.cgi?ORG=Dr&CID=80160) | - | [NM_207071](http://www.ncbi.nlm.nih.gov/entrez/viewer.fcgi?val=NM_207071) | zgc:77296 | 1.31 | 1.04E-08 | **2.02** | 9.93E-30 |
| [Dr.78299](http://www.ncbi.nlm.nih.gov/UniGene/clust.cgi?ORG=Dr&CID=78299) | - | [NM_199805](http://www.ncbi.nlm.nih.gov/entrez/viewer.fcgi?val=NM_199805) | phf17 | 1.58 | 2.68E-07 | **2.66** | 1.55E-17 |
| [Dr.79030](http://www.ncbi.nlm.nih.gov/UniGene/clust.cgi?ORG=Dr&CID=79030) | - | [AI793871](http://www.ncbi.nlm.nih.gov/entrez/query.fcgi?cmd=Search&db=Nucleotide&doptcmdl=GenBank&term=AI793871) | wu:fc55d08 | 1.77 | 4.80E-07 | **2.34** | 1.16E-22 |
| [Dr.80498](http://www.ncbi.nlm.nih.gov/UniGene/clust.cgi?ORG=Dr&CID=80498) | - | [NM_001002670](http://www.ncbi.nlm.nih.gov/entrez/viewer.fcgi?val=NM_001002670) | cryabb | 1.54 | 3.06E-06 | **2.66** | 2.47E-25 |
| Dr.108112 | - | TC295486 | LOC559239: Hypothetical LOC559239 | 1.96 | 3.85E-12 | **4.86** | 8.55E-13 |
| [Dr.83813](http://www.ncbi.nlm.nih.gov/UniGene/clust.cgi?ORG=Dr&CID=83813) | - | CK029479 | zgc:100937 | 1.66 | 2.75E-06 | **4.15** | 6.84E-21 |
| [Dr.87808](http://www.ncbi.nlm.nih.gov/UniGene/clust.cgi?ORG=Dr&CID=87808) | - | [DT862630](http://www.ensembl.org/Danio_rerio/r?d=EMBL;ID=DT862630) | [XM_678065.](http://www.ncbi.nlm.nih.gov/entrez/viewer.fcgi?db=nucleotide&val=68438796) | 1.34 | 5.50E-08 | **1.44** | 5.49E-06 |
| Dr.107342 | - | TC299873 | si:dkey-63j1.8 | 1.60 | 2.33E-11 | **2.81** | 2.90E-09 |
| Dr.80644 | Dr.7713 | NM_173256 | timm23 | 1.62 | 5.67E-06 | **1.63** | 1.61E-19 |
| Dr.80644 | Dr.7713 | NM_173256 | timm23 | **1.45** | 9.94E-12 | 1.24 | 8.02E-15 |
| Dr.82679 | Dr.11206 | BC049500 | tpi1a | 1.41 | 5.97E-06 | **1.54** | 1.04E-21 |
| Dr.77564 | Dr.25282 | NM_183072 | txnrd1 | 1.75 | 1.82E-15 | **2.51** | 3.95E-15 |
| Dr.82922 | Dr.12612 | NM_205713 | unc119.2 | 2.01 | 5.89E-27 | **2.68** | 1.17E-37 |
| Dr.39063 | Dr.1162 | NM_205613 | zgc:73237 | 1.46 | 8.42E-17 | **2.27** | 2.96E-33 |
| Dr.39063 | Dr.1162 | NM_205613 | zgc:73237 | 1.41 | 3.44E-07 | **2.04** | 3.70E-37 |
| Dr.39063 | Dr.1162 | NM_205613 | zgc:73237 | 1.36 | 3.05E-06 | **1.88** | 5.30E-20 |
| Dr.88635 | Dr.6632 | BC048135 | si:ch211-139a5.6 | 1.49 | 5.28E-11 | **4.25** | 1.21E-21 |
| Dr.36715 | Dr.36405 | [BE201126](http://www.ncbi.nlm.nih.gov/entrez/query.fcgi?cmd=Search&db=Nucleotide&doptcmdl=GenBank&term=BE201126) | wu:fk86c01 | 1.81 | 3.50E-06 | **5.20** | 2.55E-14 |
| Dr.13965 | Dr.34033 | NM_001003619 | zgc:100959 | 1.69 | 4.31E-21 | **3.14** | 2.38E-10 |
| Dr.4049 | Dr.4049 | NM_001003598 | nutf2 | 1.67 | 4.40E-25 | **2.28** | 1.14E-06 |
| Dr.85594 | Dr.15999 | NM_001004002 | zgc:101015 | 1.55 | 7.23E-06 | **5.25** | 9.18E-15 |
| Dr.87503 | Dr.36503 | NM_001004648 | zgc:101062 | **3.28** | 2.90E-06 | 2.16 | 3.75E-09 |
| Dr.76193 | Dr.26900 | NM_001003571 | dnajb1 | 1.42 | 1.42E-07 | **2.46** | 2.99E-13 |
| Dr.76743 | Dr.14834 | NM_001003551 | zgc:101136 | 1.29 | 3.69E-07 | **4.64** | 0.00E+00 |
| Dr.85433 | Dr.37048 | NM_001007777 | zgc:101581 | 1.64 | 1.21E-07 | **1.44** | 7.17E-17 |
| Dr.36953 | Dr.21263 | NM_001006088 | asah1 | 1.75 | 6.22E-31 | **2.32** | 6.28E-11 |
| Dr.36953 | Dr.21263 | NM_001006088 | asah1 | 1.54 | 9.93E-16 | **2.20** | 0.00 |
| Dr.27169 | Dr.27169 | NM_001007440 | dullardl | 1.60 | 2.60E-06 | **3.46** | 2.66E-23 |
| Dr.3508 | Dr.3508 | NM_001005769 | zgc:101819 | 1.36 | 5.26E-06 | **1.82** | 5.94E-07 |
| Dr.76140 | Dr.30331 | NM_001005960 | atp5f1 | 1.32 | 5.55E-12 | **1.38** | 3.30E-14 |
| Dr.84834 | Dr.14909 | NM_001007349 | zgc:103473 | 2.11 | 2.19E-06 | **5.89** | 4.78E-41 |
| Dr.84834 | Dr.14909 | NM_001007349 | zgc:103473 | 1.86 | 8.31E-07 | **5.81** | 1.27E-16 |
| Dr.84509 | Dr.16614 | NM_001007347 | zgc:103481 | **3.39** | 2.54E-08 | 3.07 | 1.30E-36 |
| Dr.6467 | Dr.36477 | NM_001004613 | zgc:103490 | 1.30 | 4.67E-06 | **1.82** | 0.00 |
| Dr.79955 | Dr.15371 | NM_001006016 | zgc:103512 | 1.53 | 1.58E-10 | **2.32** | 0.00 |
| Dr.20705 | Dr.20705 | NM_205714 | zgc:103537 | 1.35 | 4.44E-06 | **2.02** | 1.94E-29 |
| Dr.84460 | Dr.13656 | NM_001005986 | zgc:103544 | 1.28 | 4.19E-08 | **1.84** | 4.58E-11 |
| Dr.14969 | Dr.14969 | NM_001005982 | cln6 | 2.09 | 4.35E-07 | **2.37** | 2.29E-09 |
| Dr.14969 | Dr.14969 | NM_001005982 | cln6 | 1.98 | 1.09E-09 | **3.59** | 4.72E-10 |
| Dr.78577 | Dr.8653 | NM_001007391 | zgc:103579 | 1.41 | 6.17E-07 | **1.66** | 1.15E-08 |
| Dr.81242 | Dr.13477 | NM_001008586 | zgc:103660 | 1.28 | 6.29E-06 | **2.47** | 1.10E-36 |
| Dr.76565 | Dr.4897 | NM_001005939 | mcfd2 | 1.77 | 9.29E-06 | **2.43** | 6.15E-07 |
| Dr.85181 | Dr.15530 | NM_001005938 | park7 | 1.54 | 1.16E-14 | **2.83** | 2.49E-18 |
| Dr.85181 | Dr.15530 | NM_001005938 | park7 | 1.44 | 3.76E-10 | **2.64** | 3.27E-21 |
| Dr.81249 | Dr.11797 | NM_001006004 | pop7 | 1.27 | 2.61E-06 | **1.86** | 1.95E-21 |
| Dr.20777 | Dr.20777 | NM_199832 | tk1 | 1.35 | 3.04E-09 | **2.13** | 3.69E-08 |
| Dr.78078 | Dr.11667 | NM_213516 | zgc:55389 | 1.39 | 1.33E-07 | **1.78** | 8.58E-14 |
| Dr.81683 | Dr.20582 | NM_200259 | rbpms2 | 1.37 | 1.61E-07 | **2.66** | 1.92E-37 |
| Dr.81683 | Dr.20582 | NM_200259 | rbpms2 | 1.66 | 1.85E-24 | **3.23** | 7.15E-34 |
| Dr.81683 | Dr.20582 | NM_200259 | rbpms2 | 1.33 | 2.20E-07 | **2.74** | 1.10E-14 |
| Dr.80565 | Dr.11564 | NM_201120 | chic2 | 1.77 | 4.50E-07 | **2.35** | 7.66E-31 |
| Dr.76644 | Dr.1915 | NM_214770 | zgc:55760 | 1.42 | 3.57E-06 | **2.15** | 0.00 |
| Dr.75493 | Dr.207 | NM_214694 | zgc:55781 | 1.65 | 1.79E-07 | **2.22** | 0.00 |
| Dr.80333 | Dr.6930 | NM_200142 | zgc:55803 | 1.40 | 1.23E-08 | **1.62** | 3.88E-26 |
| Dr.9165 | Dr.9165 | NM_214707 | zgc:55843 | 1.48 | 2.71E-13 | **3.72** | 0.00 |
| Dr.81185 | Dr.11491 | NM_200151 | josd2 | 2.07 | 3.52E-23 | **4.90** | 0.00E+00 |
| [Dr.81185](http://www.ncbi.nlm.nih.gov/UniGene/clust.cgi?ORG=Dr&CID=81185) | - | [NM_200151](http://www.ncbi.nlm.nih.gov/entrez/viewer.fcgi?val=NM_200151) | josd2 | 1.84 | 3.08E-44 | **5.44** | 0.00 |
| Dr.20716 | Dr.20716 | NM_200934 | tpm1 | 1.58 | 2.98E-07 | **4.51** | 0.00 |
| Dr.83316 | Dr.15882 | NM_200193 | zgc:56093 | 1.55 | 1.91E-10 | **2.60** | 6.31E-23 |
| Dr.77147 | Dr.7343 | NM_213010 | acadm | 1.68 | 3.70E-12 | **2.43** | 1.59E-08 |
| Dr.85004 | Dr.16021 | NM_200247 | unc50 | 1.39 | 5.96E-07 | **1.54** | 6.74E-06 |
| Dr.83115 | Dr.16546 | NM_198980 | zgc:56121 | 1.62 | 8.06E-13 | **5.90** | 4.97E-28 |
| Dr.83266 | Dr.17188 | NM_201069 | zgc:56164 | 1.51 | 6.30E-12 | **2.17** | 0.00 |
| Dr.83266 | Dr.17188 | NM_201069 | zgc:56164 | 1.59 | 5.01E-26 | **2.26** | 1.15E-14 |
| Dr.75740 | Dr.9961 | NM_199937 | zgc:56239 | 1.38 | 1.97E-08 | **3.37** | 0.00 |
| Dr.79394 | Dr.7938 | NM_201215 | zgc:56248 | 1.72 | 2.76E-06 | **10.28** | 1.40E-45 |
| Dr.7757 | Dr.7757 | NM_199981 | zgc:56251 | 1.35 | 9.66E-13 | **2.20** | 2.54E-07 |
| Dr.7974 | Dr.7974 | NM_213386 | cdc45l | 2.05 | 5.60E-07 | **4.49** | 1.46E-11 |
| Dr.77494 | Dr.2062 | NM_213385 | vrk1 | 1.66 | 2.74E-28 | **2.39** | 9.77E-22 |
| Dr.1753 | Dr.1753 | NM_213369 | mospd1 | 1.30 | 5.00E-10 | **1.20** | 2.84E-06 |
| Dr.84195 | Dr.17448 | NM_200291 | psmd6 | 1.50 | 1.54E-08 | **2.03** | 0.00 |
| Dr.81346 | Dr.10970 | NM_200265 | zgc:56497 | 1.30 | 1.11E-06 | **2.26** | 1.22E-16 |
| Dr.85461 | Dr.18777 | NM_200274 | zgc:56706 | 1.47 | 1.82E-14 | **3.33** | 0.00 |
| Dr.80528 | Dr.28446 | NM_213528 | zgc:63476 | 1.34 | 6.59E-08 | **2.07** | 5.65E-14 |
| Dr.80291 | Dr.12730 | NM_200438 | zgc:63574 | 1.52 | 4.00E-14 | **1.93** | 6.85E-06 |
| Dr.83242 | Dr.20655 | NM_200484 | zgc:63691 | 1.39 | 4.60E-09 | **3.07** | 3.21E-29 |
| Dr.26674 | Dr.26674 | NM_201180 | zgc:63701 | 1.69 | 5.79E-15 | **2.25** | 5.58E-07 |
| Dr.77007 | Dr.4831 | NM_199584 | hiat1a | 1.35 | 1.84E-08 | **1.60** | 5.64E-27 |
| [Dr.77778](http://www.ncbi.nlm.nih.gov/UniGene/clust.cgi?ORG=Dr&CID=77778) | - | NM_214763 | zgc:63990 | 1.23 | 9.07E-07 | **1.54** | 7.08E-24 |
| Dr.18807 | Dr.18807 | NM_201185 | bokb | 1.71 | 1.93E-32 | **3.14** | 8.11E-08 |
| Dr.79579 | Dr.5406 | NM_200582 | trip13 | 1.52 | 3.51E-06 | **3.81** | 5.27E-15 |
| Dr.76732 | Dr.28416 | NM_199554 | zgc:66198 | 1.44 | 1.50E-13 | **3.06** | 3.49E-39 |
| Dr.80345 | Dr.7273 | NM_199827 | zgc:66484 | 1.79 | 3.99E-11 | **1.95** | 1.44E-17 |
| Dr.85366 | Dr.27143 | NM_200790 | zgc:73327 | 1.38 | 4.57E-07 | **2.66** | 0.00 |
| Dr.77826 | Dr.3099 | NM_199643 | coro2a | 1.53 | 2.39E-06 | **1.66** | 0.00 |
| Dr.75409 | Dr.6237 | [BC066528](http://www.ncbi.nlm.nih.gov/entrez/query.fcgi?cmd=Search&db=Nucleotide&doptcmdl=GenBank&term=BC066528) | gapdhs | 1.46 | 9.06E-07 | **2.27** | 1.08E-12 |
| Dr.21599 | Dr.21599 | NM_212672 | atp6v0a1 | 2.31 | 5.12E-17 | **2.37** | 4.20E-10 |
| Dr.4206 | Dr.4206 | NM_214757 | zgc:76977 | 1.78 | 5.20E-14 | **7.37** | 0.00 |
| Dr.17401 | Dr.17401 | NM_205555 | pars2 | 1.85 | 2.67E-09 | **2.57** | 0.00 |
| Dr.78116 | Dr.7615 | NM_205566 | arl8b | 2.31 | 1.73E-38 | **3.88** | 5.35E-10 |
| Dr.29812 | Dr.29812 | NM_205699 | zgc:77318 | 1.50 | 2.30E-08 | **1.60** | 3.32E-23 |
| Dr.30522 | Dr.30522 | NM_205759 | zgc:77395 | 1.63 | 1.08E-06 | **2.03** | 1.38E-08 |
| Dr.30522 | Dr.30522 | NM_205759 | zgc:77395 | 1.57 | 7.23E-11 | **2.44** | 7.03E-11 |
| Dr.4850 | Dr.4850 | NM_212615 | rnf128 | 1.23 | 1.45E-06 | **2.33** | 1.94E-11 |
| Dr.84043 | Dr.13273 | NM_001002318 | zgc:86635 | 1.33 | 6.18E-06 | **2.09** | 7.90E-08 |
| Dr.84043 | Dr.13273 | NM_001002318 | zgc:86635 | 1.38 | 7.29E-10 | **2.09** | 6.15E-07 |
| Dr.80631 | Dr.28391 | NM_001002324 | zdhhc24 | 1.54 | 1.35E-11 | **5.00** | 0.00 |
| Dr.31220 | Dr.31220 | NM_001002661 | zgc:91969 | 1.66 | 1.32E-06 | **3.87** | 9.08E-20 |
| Dr.2818 | Dr.2818 | NM_001004114 | zgc:91986 | 1.74 | 1.07E-07 | **2.17** | 1.04E-09 |
| Dr.76761 | Dr.26014 | NM_001003988 | zgc:91996 | 1.47 | 1.27E-09 | **1.93** | 7.13E-19 |
| Dr.78435 | Dr.31713 | NM_001004113 | zgc:91997 | 1.69 | 1.95E-07 | **2.63** | 2.91E-06 |
| Dr.32775 | Dr.7143 | NM_001002645 | zgc:92030 | **1.58** | 8.19E-10 | 1.69 | 9.63E-06 |
| Dr.81507 | Dr.11481 | NM_001002352 | zgc:92153 | 1.75 | 6.86E-06 | **5.57** | 9.19E-27 |
| Dr.81554 | Dr.23018 | NM_001004560 | zgc:92250 | 1.46 | 2.01E-08 | **2.42** | 2.40E-33 |
| Dr.75681 | Dr.31386 | NM_001002620 | coq3 | 1.40 | 8.10E-08 | **1.87** | 3.10E-09 |
| Dr.84500 | Dr.13633 | NM_001002606 | zgc:92287 | 1.56 | 1.71E-07 | **2.78** | 3.34E-40 |
| Dr.28994 | Dr.28994 | NM_001002211 | ppapdc1b | 1.78 | 1.66E-23 | **3.36** | 2.33E-18 |
| Dr.84847 | Dr.34247 | NM_001003996 | zgc:92404 | 1.66 | 1.46E-12 | **2.25** | 8.02E-17 |
| Dr.80269 | Dr.36652 | NM_001005935 | zgc:92579 | 2.25 | 7.69E-09 | **2.78** | 3.29E-29 |
| Dr.80269 | Dr.36652 | NM_001005935 | zgc:92579 | 3.60 | 2.41E-12 | **4.39** | 0.00 |
| Dr.86107 | Dr.31681 | NM_001002431 | zgc:92650 | **2.02** | 4.74E-08 | 1.96 | 1.46E-08 |
| Dr.2219 | Dr.2219 | NM_001002547 | zgc:92785 | 1.38 | 4.25E-14 | **2.09** | 4.22E-22 |
| Dr.75282 | Dr.12983 | NM_001002497 | zgc:92842 | 1.46 | 4.72E-06 | **2.07** | 5.32E-07 |
| Dr.82332 | Dr.11449 | NM_001002467 | adipor1a | 1.32 | 2.23E-07 | **3.20** | 1.21E-14 |
| Dr.28485 | Dr.15217 | NM_200979 | znf143 | 1.50 | 8.98E-15 | **2.55** | 8.61E-10 |
| Dr.75734 | Dr.26977 | NM_131696 | zp3b | 1.67 | 1.47E-09 | **2.31** | 0.00 |
| Dr.75734 | Dr.26977 | NM_131696 | zp3b | 1.76 | 1.05E-06 | **2.02** | 1.18E-07 |

**Bold** = >2-fold change

**Table S5;** *ERK1 knockdown specific genes at 30% epiboly, filtered by an 1.5 fold up- or down- regulation per experiment and a common P-value of 10^-5^*

| **Unigene**  **nov 2006** | **Unigene**  **old** | **Accession** | **Sequence Name(s)** | **Fold Change**  **ERK1MO** | **P-value**  **ERK1MO** |
| --- | --- | --- | --- | --- | --- |
| Dr.76464 | Dr.1894 | NM_001005942 | glrx | **-4.87** | 2.80E-06 |
| Dr.132849 | Dr.5985 | CK399999 | wu:fj54e12 | **-3.17** | 3.58E-06 |
| Dr.77277 | - | [AI544617](http://www.ensembl.org/Danio_rerio/r?d=EMBL;ID=AI544617) | lmo1 | **-3.00** | 3.91E-09 |
| [Dr.32450](http://www.ncbi.nlm.nih.gov/UniGene/clust.cgi?ORG=Dr&CID=32450) | - | [NM_212784](http://www.ncbi.nlm.nih.gov/entrez/viewer.fcgi?val=NM_212784) | rpl13a | **-2.84** | 1.86E-06 |
| Dr.77880 | Dr.5038 | NM_131762 | cldna | **-2.70** | 2.86E-07 |
| [Dr.14471](http://www.ncbi.nlm.nih.gov/UniGene/clust.cgi?ORG=Dr&CID=14471) | - | [NM_001002753](http://www.ncbi.nlm.nih.gov/entrez/viewer.fcgi?val=NM_001002753) | zgc:100838 | **-2.70** | 2.07E-06 |
| Dr.31375 | Dr.31375 | NM_001002479 | zgc:92873 | **-2.57** | 1.37E-06 |
| Dr.14471 | Dr.14471 | NM_001002753 | zgc:100838 | **-2.52** | 8.14E-07 |
| Dr.88996 | Dr.26255 | CK394877 | Transcribed locus | **-2.49** | 4.60E-09 |
| Dr.21056 | Dr.21056 | NM_199608 | zgc:77734 | **-2.08** | 6.87E-07 |
| Dr.28905 | Dr.28905 | CF999386 | Transcribed locus | **-2.02** | 8.47E-06 |
| Dr.90441 | Dr.29213 | CK360168 | Transcribed locus | -1.98 | 3.86E-08 |
| Dr.28410 | Dr.35045 | AI618852 | krt5: Keratin 5 | -1.93 | 5.65E-06 |
| Dr.79924 | Dr.36434 | NM_001005604 | LOC449616: Bcl9 | -1.81 | 1.89E-06 |
| Dr.80715 | Dr.20972 | NM_199887 | msto1 | -1.80 | 1.13E-06 |
| Dr.8283 | Dr.8283 | BC050155 | mibp | -1.76 | 7.91E-06 |
| Dr.20345 | Dr.20345 | NM_001002041 | flj12949l | -1.71 | 1.75E-07 |
| Dr.78435 | Dr.31713 | NM_001004113 | zgc:91997 | 1.69 | 1.95E-07 |
| Dr.79394 | Dr.7938 | NM_201215 | zgc:56248 | 1.72 | 2.76E-06 |
| [Dr.15144](http://www.ncbi.nlm.nih.gov/UniGene/clust.cgi?ORG=Dr&CID=15144) | - | [NM_200676](http://www.ncbi.nlm.nih.gov/entrez/viewer.fcgi?val=NM_200676) | zgc:66030 | 1.74 | 5.37E-07 |
| Dr.75734 | Dr.26977 | NM_131696 | zp3b | 1.76 | 1.05E-06 |
| [Dr.79030](http://www.ncbi.nlm.nih.gov/UniGene/clust.cgi?ORG=Dr&CID=79030) | - | [AI793871](http://www.ncbi.nlm.nih.gov/entrez/query.fcgi?cmd=Search&db=Nucleotide&doptcmdl=GenBank&term=AI793871) | wu:fc55d08 | 1.77 | 4.80E-07 |
| Dr.80565 | Dr.11564 | NM_201120 | chic2 | 1.77 | 4.50E-07 |
| Dr.76565 | Dr.4897 | NM_001005939 | mcfd2 | 1.77 | 9.29E-06 |
| Dr.75898 | Dr.15173 | NM_212997 | acbd3 | 1.78 | 2.69E-07 |
| Dr.36715 | Dr.36405 | CN835779 | wu:fk86c01 | 1.81 | 3.50E-06 |
| Dr.78550 | Dr.15607 | BC049494 | LOC796309: Similar to Cee | 1.86 | 5.25E-07 |
| Dr.86302 | Dr.17236 | BC090312 | zgc:113297 | **2.00** | 1.72E-07 |
| Dr.7974 | Dr.7974 | NM_213386 | cdc45l | **2.05** | 5.60E-07 |
| Dr.78793 | Dr.2675 | NM_213149 | fkbp5 | **2.11** | 8.07E-06 |
| Dr.27161 | Dr.27161 | NM_213395 | spg21 | **2.14** | 4.61E-06 |
| [Dr.78119](http://www.ncbi.nlm.nih.gov/entrez/query.fcgi?db=unigene&term=Dr.30111) | - | NM_001076738.1 | Zgc:153556 | **2.23** | 4.52E-06 |
| [Dr.78284](http://www.ncbi.nlm.nih.gov/UniGene/clust.cgi?ORG=Dr&CID=78284) | - | TC292195 | zgc:109987 | **2.24** | 1.94E-08 |
| Dr.85467 | Dr.30214 | NM_207078 | zgc:76981 | **2.43** | 1.82E-07 |
| Dr.673 | Dr.673 | BC052753 | rtn1l | **2.55** | 2.98E-06 |
| Dr.12618 | - | TC273224 | efnb2b: Ephrin B2b | **2.63** | 8.84E-09 |
| Dr.13791 | Dr.13791 | NM_200664 | igsf4d | **2.81** | 5.89E-07 |
| Dr.3454 | Dr.3454 | NM_131765 | cldne | **2.87** | 2.93E-09 |
| Dr.31771 | - | [NM_001002378](http://www.ncbi.nlm.nih.gov/UniGene/seq.cgi?ORG=Dr&SID=21312712) | Zgc:92066 | **3.21** | 6.47E-06 |
| Dr.87503 | Dr.36503 | NM_001004648 | zgc:101062 | **3.28** | 2.90E-06 |
| Dr.90122 | Dr.31187 | NM_001002636 | khk | **3.41** | 1.36E-07 |
| Dr.23445 | Dr.23445 | NM_131021 | ba2 | **3.47** | 2.82E-09 |
| Dr.11380 | - | [BI889345.1](http://www.ncbi.nlm.nih.gov/UniGene/seq.cgi?ORG=Dr&SID=12185002) | LOC558396: Hypothetical LOC558396 | **3.58** | 1.59E-07 |
| Dr.81961 | - | NM_001002472 | zgc:92885 | **3.72** | 2.54E-08 |
| - | Dr.10068 | BE201398 | fk92a05.x1 | **3.75** | 1.67E-07 |
| Dr.76170 | - | TC291917 | zgc:55429 | **3.93** | 9.65E-06 |
| Dr.79526 | Dr.25774 | BC066559 | LOC560400: Similar to non-muscle alpha-actinin 1 | **4.82** | 9.41E-06 |
| Dr.115550 | Dr.36611 | BC091560 | Capon: Capon protein | **5.05** | 4.59E-08 |
| Dr.83334 | Dr.12403 | NM_131633 | robo2 | **6.21** | 1.92E-08 |
| Dr.82165 | Dr.10111 | NM_001004625 | zgc:101772 | **6.82** | 6.37E-13 |
| Dr.82210 | Dr.23332 | CK707021 | Transcribed locus | **9.32** | 6.33E-18 |
| Dr.24982 | Dr.24982 | BC066622 | zgc:56585 | **11.69** | 4.94E-18 |

**Bold** = >2-fold change

**Table S6**; *ERK2 knockdown specific genes at 30% epiboly, filtered by an 1.5 fold up- or down- regulation per experiment and a common P-value of 10^-5^*

| **Unigene**  nov 2006 | **Unigene**  old | **Accession** | **Sequence Name(s)** | **Fold Change**  **ERK2MO** | **P-value**  **ERK2MO** |
| --- | --- | --- | --- | --- | --- |
| Dr.45492 |  | NM_001020477 | zgc:110689 | **-33.15** | 0.00E+00 |
| Dr.74463 | Dr.31497 | AW202835 | wu:fb15g10 | **-32.72** | 3.83E-32 |
| Dr.75775 |  | TC268493 | apoa1: Apolipoprotein A-I | **-23.93** | 9.88E-21 |
| Dr.3265 | Dr.28454 | NM_131107 | cyt1 | **-20.18** | 0.00E+00 |
| Dr.29718 | Dr.29718 | NM_152980 | mst1 | **-18.77** | 6.92E-25 |
| Dr.133287 | Dr.18268 | CN329188 | LOC100002042: Hypothetical protein LOC100002042 | **-18.12** | 0.00E+00 |
| Dr.134727 | Dr.29266 | CK361216 | Transcribed locus | **-17.97** | 3.09E-24 |
| Dr.21720 | Dr.3 | BC059426 | atp1b3a | **-15.43** | 7.12E-10 |
| Dr.77285 |  | AI546031 | wu:fb77c11 | **-14.56** | 0.00E+00 |
| Dr.18834 | Dr.18834 | NM_001002332 | zgc:92414 | **-13.44** | 0.00E+00 |
| Dr.77124 | Dr.36577 | NM_212634 | nlcam | **-13.32** | 0.00E+00 |
| Dr.17225 | Dr.17225 | BM259622 | sb:cb124 | **-12.84** | 0.00E+00 |
| Dr.27007 | Dr.27007 | BC084686 | ldb4 | **-12.66** | 0.00E+00 |
| Dr.47576 | Dr.31750 | AL730922 | zgc:110069 | **-12.23** | 0.00E+00 |
| Dr.80323 | Dr.24216 | NM_131108 | cki | **-11.99** | 0.00E+00 |
| Dr.25823 | Dr.8109 | NM_131320 | lcp1 | **-11.82** | 2.40E-29 |
| Dr.74550 | Dr.29132 | NM_199624 | her8a | **-11.64** | 0.00E+00 |
| Dr.77402 | Dr.12273 | NM_001006094 | rab34 | **-10.89** | 0.00E+00 |
| Dr.47576 |  | NM_001025301 | zgc:110069 | **-10.70** | 4.55E-32 |
| Dr.24982 | Dr.24982 | NM_200327 | zgc:56585 | **-10.56** | 0.00E+00 |
| Dr.75704 |  | NM_213634 | ptgds | **-9.95** | 5.00E-09 |
| Dr.32396 | Dr.29413 | NM_001002383 | zgc:92061 | **-9.69** | 9.70E-28 |
| Dr.133319 | Dr.28488 | CF999453 | Transcribed locus | **-9.53** | 0.00E+00 |
| Dr.4035 | Dr.4011 | CF416997 | sb:cb793 | **-9.28** | 0.00E+00 |
| Dr.77145 | Dr.4194 | NM_213271 | hnrpul1 | **-9.16** | 0.00E+00 |
| Dr.31918 | Dr.34784 | CF673261 | sb:cb916 | **-8.99** | 6.22E-13 |
| Dr.32396 | Dr.29413 | NM_001002383 | zgc:92061 | **-8.97** | 0.00E+00 |
| Dr.105560 |  | TC281497 | Transcribed locus | **-8.87** | 0.00E+00 |
| Dr.78256 | Dr.25629 | NM_199279 | ivns1abpa | **-8.78** | 4.45E-18 |
| Dr.567 | Dr.567 | NM_131342 | bmp4 | **-8.73** | 4.20E-45 |
| Dr.75074 | Dr.1401 | NM_131114 | eve1 | **-8.51** | 6.82E-14 |
| Dr.12618 |  | TC273224 | efnb2b: Ephrin B2b | **-8.44** | 3.06E-18 |
| Dr.5206 | Dr.5206 | NM_131850 | aldh1a2 | **-8.33** | 7.59E-15 |
| Dr.77287 | Dr.4236 | NM_001003501 | zgc:92451 | **-8.20** | 1.65E-27 |
| Dr.77429 | Dr.34060 | NM_001003521 | zgc:100900 | **-8.13** | 0.00E+00 |
| Dr.76526 | Dr.4129 | NM_173275 | lamb1 | **-8.13** | 4.42E-13 |
| Dr.81280 | Dr.36260 | BG985631 | id:ibd5075 | **-8.12** | 7.09E-15 |
| Dr.76839 | Dr.23662 | NM_205721 | pdcl3 | **-8.06** | 9.69E-14 |
| Dr.35945 | Dr.1251 | NM_212700 | degs | **-8.01** | 0.00E+00 |
| Dr.34675 | Dr.12425 | NM_001003445 | zgc:92533 | **-7.50** | 7.27E-21 |
| Dr.42225 |  | BI889098.1 | LOC555722: Hypothetical LOC555722 | **-7.38** | 1.53E-36 |
| Dr.79347 | Dr.51674 | AW777758 | wu:fk50c04 | **-7.32** | 5.06E-31 |
| Dr.83294 | Dr.12373 | NM_001002347 | zgc:92178 | **-7.22** | 5.96E-25 |
| Dr.18556 | Dr.18556 | BC064328 | twistnb | **-7.21** | 1.09E-26 |
| Dr.11310 | Dr.11310 | BC067554 | tuba1 | **-7.14** | 1.44E-17 |
| Dr.75111 | Dr.4387 | NM_131509 | krt4 | **-7.13** | 5.87E-43 |
| Dr.2442 | Dr.2442 | BI889131 | wu:fb48g11 | **-7.02** | 5.72E-32 |
| Dr.79656 | Dr.13155 | AI959094 | AI959094 | **-7.01** | 0.00E+00 |
| Dr.54134 | Dr.14294 | NM_199941 | serpinb1l4 | **-6.95** | 2.66E-19 |
| Dr.90502 | Dr.36824 | NM_001005948 | zgc:103657 | **-6.89** | 1.40E-12 |
| Dr.673 |  | NM_001029948 | rtn1b | **-6.35** | 1.26E-27 |
| Dr.75111 | Dr.4387 | NM_131509 | krt4 | **-6.31** | 1.35E-14 |
| Dr.16181 |  | NM_213239 | zgc:77260 | **-6.23** | 2.82E-13 |
| Dr.105756 |  | NM_001045191 | LOC569877: Similar to DNA topoisomeraseII_beta | **-6.22** | 2.48E-36 |
| Dr.35945 | Dr.1251 | NM_212700 | degs | **-5.99** | 1.36E-16 |
| Dr.75326 | Dr.8346 | NM_001007311 | zgc:92355 | **-5.90** | 0.00E+00 |
| Dr.25620 | Dr.25620 | AL717158.1 | Transcribed locus | **-5.73** | 1.86E-13 |
| Dr.32146 | Dr.32146 | NM_001003873 | rbbp6l | **-5.63** | 4.95E-21 |
| Dr.673 | Dr.673 | BC052753 | rtn1b | **-5.58** | 0.00E+00 |
| Dr.77459 | Dr.3292 | NM_200297 | mgll | **-5.57** | 2.85E-14 |
| Dr.118880 | Dr.29278 | BC078312 | LOC553243: Hypothetical protein LOC553243 | **-5.57** | 0.00E+00 |
| Dr.79053 | Dr.3269 | NM_130934 | hhex | **-5.56** | 0.00E+00 |
| Dr.80627 | Dr.27797 | CK684195 | b3gat3 | **-5.55** | 1.83E-22 |
| Dr.59 | Dr.59 | NM_181758 | anxa1a | **-5.45** | 2.46E-10 |
| Dr.75499 | Dr.4845 | NM_212614 | mycn | **-5.37** | 2.50E-14 |
| Dr.29744 | Dr.29744 | NM_205550 | sp8l | **-5.30** | 1.02E-14 |
| Dr.87413 | Dr.30254 | NM_212794 | sertad2 | **-5.26** | 8.17E-14 |
| Dr.88822 | Dr.34065 | NM_001003461 | zgc:92423 | **-5.19** | 1.52E-07 |
| Dr.83299 |  | NM_001017629 | zgc:110243 | **-5.18** | 6.12E-17 |
| Dr.78256 | Dr.25629 | NM_199279 | ivns1abpa | **-5.18** | 7.32E-06 |
| Dr.18783 | Dr.18783 | BC091681 | zgc:113006 | **-5.09** | 1.43E-35 |
| Dr.76425 | Dr.32225 | CO351282 | si:busm1-228j01.1 | **-5.05** | 0.00E+00 |
| Dr.28660 | Dr.28660 | NM_199524 | clica | **-5.04** | 8.41E-18 |
| Dr.486 | Dr.486 | NM_001002692 | ppp1r14b | **-5.02** | 4.27E-25 |
| Dr.76313 | Dr.38304 | CK027604 | adka | **-4.96** | 2.88E-12 |
| Dr.80737 |  | NM_001017591 | zgc:110307 | **-4.94** | 0.00E+00 |
| Dr.26340 | Dr.50035 | AI658138 | wu:fc22g07 | **-4.86** | 0.00E+00 |
| Dr.41525 |  | NM_001040337 | zgc:136360 | **-4.85** | 0.00E+00 |
| Dr.17518 | Dr.17518 | CN024142 | LOC558435: Hypothetical LOC558435 | **-4.80** | 9.22E-06 |
| Dr.40014 |  | NM_001017594 | zgc:110300 | **-4.65** | 0.00E+00 |
| Dr.43204 | Dr.43204 | AI497193 | epyc | **-4.56** | 7.43E-10 |
| Dr.32651 |  | TC279036 | cyt1: Type I cytokeratin, enveloping layer | **-4.53** | 6.02E-06 |
| Dr.36534 | Dr.467 | NM_001004598 | zgc:92093 | **-4.47** | 0.00E+00 |
| Dr.77202 | Dr.2961 | NM_199609 | rdh1l | **-4.44** | 6.79E-07 |
| Dr.78563 | Dr.17743 | NM_198374 | zgc:66482 | **-4.43** | 0.00E+00 |
| Dr.31402 |  | NM_001017565 | zgc:110552 | **-4.36** | 1.15E-18 |
| Dr.91146 | Dr.29934 | NM_001007065 | trpa1a | **-4.20** | 3.46E-11 |
| Dr.77934 | Dr.25534 | NM_205585 | sfrp1 | **-4.18** | 0.00E+00 |
| Dr.76631 | Dr.15424 | NM_194378 | arpc5a | **-4.15** | 1.40E-45 |
| Dr.76519 | Dr.7311 | NM_001002297 | gnl3 | **-4.14** | 1.26E-12 |
| Dr.75804 | Dr.13384 | NM_130973 | chd | **-4.07** | 9.01E-18 |
| Dr.78563 | Dr.17743 | NM_198374 | zgc:66482 | **-4.05** | 0.00E+00 |
| Dr.76010 |  | NM_173262 | rpl36a | **-4.04** | 1.42E-15 |
| Dr.79332 | Dr.9710 | AI883431 | zgc:136864 | **-4.01** | 2.33E-06 |
| Dr.75541 |  | NM_001020822 | zgc:110692 | **-3.96** | 4.55E-08 |
| Dr.77625 | Dr.28650 | NM_205743 | ARAF | **-3.95** | 2.63E-11 |
| Dr.77093 |  | NM_194395 | birc2 | **-3.89** | 0.00E+00 |
| Dr.77943 | Dr.5070 | NM_199651 | zgc:55876 | **-3.89** | 8.02E-18 |
| Dr.9060 | Dr.1335 | NM_212952 | rpl36 | **-3.84** | 2.15E-18 |
| Dr.77049 | Dr.1835 | NM_194368 | hnf4a | **-3.84** | 1.64E-16 |
| Dr.77771 |  | NM_001075103 | si:dkey-252h13.6 | **-3.83** | 1.75E-08 |
| Dr.78782 | Dr.21590 | NM_201583 | c2gnt3 | **-3.82** | 2.26E-19 |
| Dr.23693 | Dr.23693 | NM_001007457 | zgc:101606 | **-3.82** | 7.42E-11 |
| Dr.77706 | Dr.19364 | BM141616 | zgc:86602 | **-3.79** | 9.24E-09 |
| Dr.134752 |  | TC278257 | Transcribed locus | **-3.75** | 0.00E+00 |
| Dr.80099 | Dr.12057 | NM_200883 | gne | **-3.73** | 0.00E+00 |
| Dr.37875 | Dr.12978 | CN318354 | LOC562862: Similar to nucleoporin 214kDa | **-3.68** | 1.41E-33 |
| Dr.39283 | Dr.4434 | NM_213328 | zgc:77495 | **-3.65** | 1.20E-31 |
| Dr.3664 | Dr.3664 | NM_001005954 | zgc:103627 | **-3.63** | 3.05E-23 |
| Dr.77964 | Dr.5074 | NM_201124 | arrb2 | **-3.61** | 1.30E-13 |
| Dr.77450 | Dr.34965 | BG985584 | id:ibd5033 | **-3.60** | 7.33E-12 |
| Dr.77277 |  | NM_173219 | lmo1 | **-3.59** | 4.62E-08 |
| Dr.32607 | Dr.369 | NM_213278 | sfpq | **-3.58** | 0.00E+00 |
| Dr.26808 | Dr.26808 | NM_199636 | rpl10a | **-3.54** | 1.30E-20 |
| Dr.30688 | Dr.30688 | CN321890 | Transcribed locus | **-3.51** | 9.60E-09 |
| Dr.26808 | Dr.26808 | NM_199636 | rpl10a | **-3.49** | 1.81E-18 |
| Dr.76007 | Dr.881 | NM_213265 | zgc:85975 | **-3.48** | 5.55E-08 |
| Dr.86119 | Dr.37201 | NM_001007427 | zgc:101706 | **-3.48** | 3.28E-16 |
| Dr.75804 |  | NM_130973 | chd | **-3.46** | 6.84E-09 |
| Dr.32845 | Dr.8830 | NM_130918 | fzd8a | **-3.46** | 2.81E-36 |
| Dr.78163 |  | AI617258 | LOC100000660 | **-3.42** | 0.00E+00 |
| Dr.80506 |  | NM_001017603 | zgc:110285 | **-3.40** | 4.98E-13 |
| Dr.78473 | Dr.8120 | NM_130983 | nog1 | **-3.38** | 0.00E+00 |
| Dr.52550 | Dr.52301 | AI584988 | wu:fb93g11 | **-3.37** | 1.39E-13 |
| Dr.38212 | Dr.17863 | BC044499 | flot2a | **-3.36** | 0.00E+00 |
| Dr.8006 | Dr.8006 | NM_213451 | zgc:63783 | **-3.35** | 2.49E-10 |
| Dr.193 | Dr.193 | NM_131406 | rara2a | **-3.34** | 2.83E-20 |
| Dr.81870 | Dr.34320 | NM_001005959 | zgc:101891 | **-3.32** | 0.00E+00 |
| Dr.80110 | Dr.6884 | NM_201514 | zgc:55813 | **-3.32** | 1.83E-17 |
| Dr.105756 |  | BX927238 | si:dkey-98n4.1 | **-3.31** | 9.85E-10 |
| Dr.79112 | Dr.22406 | NM_213343 | srp19 | **-3.31** | 5.90E-37 |
| Dr.32635 |  | NM_001003412 | clint1 | **-3.29** | 2.20E-11 |
| Dr.84315 | Dr.29943 | NM_001006080 | zgc:101673 | **-3.27** | 6.74E-27 |
| Dr.75570 | Dr.541 | NM_131245 | id1 | **-3.26** | 1.11E-09 |
| Dr.76383 | Dr.27010 | NM_001002190 | zgc:91870 | **-3.25** | 1.52E-07 |
| Dr.32854 |  | NM_213279 | rps2 | **-3.23** | 0.00E+00 |
| Dr.32617 | Dr.345 | NM_153673 | unc45r | **-3.22** | 2.44E-18 |
| Dr.135287 | Dr.31324 | CV483059 | Transcribed locus | **-3.21** | 0.00E+00 |
| Dr.34675 | Dr.12425 | NM_001003445 | zgc:92533 | **-3.21** | 1.40E-11 |
| Dr.75111 | Dr.4387 | NM_131509 | krt4 | **-3.20** | 1.98E-08 |
| Dr.75382 | Dr.24766 | NM_131027 | smoh | **-3.19** | 8.72E-07 |
| Dr.75609 |  | NM_001076558 | zgc:153435 | **-3.18** | 4.99E-18 |
| Dr.77489 | Dr.31968 | NM_001003874 | flj11749l | **-3.18** | 1.62E-08 |
| Dr.18200 | Dr.18200 | NM_001002480 | zgc:92871 | **-3.15** | 1.16E-18 |
| Dr.33193 | Dr.20181 | NM_213277 | anp32e | **-3.13** | 6.96E-24 |
| Dr.75572 |  | NM_131444 | gnb2l1 | **-3.13** | 2.38E-37 |
| Dr.24212 |  | NM_001040321 | zgc:136559 | **-3.13** | 1.11E-08 |
| Dr.76383 | Dr.27010 | NM_001002190 | zgc:91870 | **-3.11** | 0.00E+00 |
| Dr.80110 | Dr.6884 | NM_201514 | zgc:55813 | **-3.01** | 1.82E-16 |
| Dr.26893 |  | NM_194394 | mcl1b | **-3.00** | 2.02E-21 |
| Dr.76719 |  | NM_213333 | sltm | **-3.00** | 0.00E+00 |
| Dr.22101 | Dr.36685 | NM_001012492 | zgc:113436 | **-2.99** | 2.76E-06 |
| Dr.79131 | Dr.30314 | NM_001001844 | ctcf | **-2.97** | 0.00E+00 |
| Dr.77202 | Dr.2961 | NM_199609 | rdh1l | **-2.95** | 1.11E-28 |
| Dr.75506 | Dr.5470 | NM_214760 | rqcd1 | **-2.94** | 1.51E-32 |
| Dr.79556 | Dr.14044 | NM_001002691 | gpr137bb | **-2.94** | 0.00E+00 |
| Dr.75058 | Dr.409 | NM_131430 | fgfr4 | **-2.92** | 3.87E-14 |
| Dr.75946 | Dr.28457 | NM_213133 | zgc:65840 | **-2.87** | 0.00E+00 |
| Dr.75615 |  | NM_200017 | eef1d: Elongation factor-1, delta | **-2.83** | 0.00E+00 |
| Dr.75507 | Dr.19753 | NM_212970 | mtp | **-2.76** | 6.71E-07 |
| Dr.34631 |  | NM_200028 | rpl30 | **-2.76** | 0.00E+00 |
| Dr.11978 | Dr.11978 | NM_200405 | hif1al | **-2.75** | 0.00E+00 |
| Dr.11978 | Dr.11978 | NM_200405 | hif1al | **-2.73** | 0.00E+00 |
| Dr.75559 |  | NM_212645 | zgc:77366 | **-2.72** | 4.03E-10 |
| Dr.82673 | Dr.16118 | BI878304 | LOC567833: Similar to microtubule-associated protein tau | **-2.71** | 1.31E-08 |
| Dr.77059 |  | NM_213494 | aebp2 | **-2.71** | 2.63E-06 |
| Dr.87419 | Dr.31449 | NM_001002570 | zgc:92749 | **-2.70** | 4.91E-07 |
| Dr.83940 | Dr.13104 | NM_200422 | zgc:64214 | **-2.70** | 0.00E+00 |
| Dr.78312 | Dr.24975 | NM_200314 | zgc:56104 | **-2.69** | 9.49E-09 |
| Dr.75231 | Dr.3199 | AW078179 | cherp | **-2.63** | 5.84E-11 |
| Dr.83940 | Dr.13104 | NM_200422 | zgc:64214 | **-2.62** | 1.73E-30 |
| Dr.85361 | Dr.30892 | BC080249 | zgc:110549 | **-2.61** | 1.84E-14 |
| Dr.76622 | Dr.4766 | NM_001004604 | zgc:92481 | **-2.59** | 7.23E-08 |
| Dr.18287 | Dr.18287 | NM_131069 | cx43.4 | **-2.58** | 6.68E-41 |
| Dr.78633 | Dr.23472 | NM_180973 | sp5 | **-2.58** | 0.00E+00 |
| Dr.30332 | Dr.30332 | AI106348 | rpl7 | **-2.57** | 0.00E+00 |
| Dr.20274 | Dr.20274 | NM_213356 | ubin | **-2.53** | 4.76E-26 |
| Dr.5793 | Dr.5793 | NM_200946 | zgc:55327 | **-2.53** | 0.00E+00 |
| Dr.37918 | Dr.4762 | CN511960 | nfya | **-2.52** | 2.47E-16 |
| Dr.77235 | Dr.12624 | NM_131823 | irx1b | **-2.50** | 6.96E-15 |
| Dr.81130 | Dr.46157 | AW282020 | map2k1 | **-2.50** | 9.76E-13 |
| Dr.39128 |  | NM_178437 | krt18 | **-2.48** | 1.36E-06 |
| Dr.48550 | Dr.30592 | NM_001001592 | hnrpu | **-2.46** | 0.00E+00 |
| Dr.36960 | Dr.28340 | NM_001005583 | zgc:91861 | **-2.45** | 0.00E+00 |
| Dr.75673 |  | BC115111 | LOC553506: Hypothetical protein LOC553506 | **-2.44** | 1.71E-31 |
| Dr.11310 |  | NM_194388 | tuba1 | **-2.44** | 1.08E-26 |
| Dr.78323 | Dr.32137 | NM_001002872 | parva | **-2.42** | 8.94E-06 |
| Dr.81921 | Dr.26961 | NM_200536 | zgc:66022 | **-2.42** | 4.85E-24 |
| Dr.48550 | Dr.30592 | NM_001001592 | hnrpu | **-2.41** | 0.00E+00 |
| Dr.8070 | Dr.8070 | NM_131158 | dbx1a | **-2.40** | 1.16E-06 |
| Dr.79753 |  | AW154460 | wu:fi29b07 | **-2.39** | 1.91E-07 |
| Dr.32854 | Dr.1126 | NM_213279 | rps2 | **-2.37** | 0.00E+00 |
| Dr.7036 |  | NM_199956 | g3bp1 | **-2.37** | 9.13E-30 |
| Dr.76899 |  | NM_201217 | nol5a | **-2.36** | 7.45E-11 |
| Dr.77202 | Dr.2961 | NM_199609 | rdh1l | **-2.35** | 4.58E-14 |
| Dr.32300 |  | NM_213262 | zgc:77429 | **-2.34** | 0.00E+00 |
| Dr.23502 | Dr.1246 | NM_131098 | apoeb | **-2.33** | 3.62E-07 |
| Dr.5793 | Dr.5793 | NM_200946 | zgc:55327 | **-2.33** | 0.00E+00 |
| Dr.10261 | Dr.10261 | NM_213223 | zgc:85717 | **-2.32** | 2.77E-22 |
| Dr.79544 |  | NM_001007168 | ing5b | **-2.32** | 4.78E-42 |
| Dr.132204 | Dr.32854 | AI957555 | LOC100004769: Similar to Ribosomal protein S2 | **-2.31** | 3.91E-07 |
| Dr.3664 | Dr.3664 | NM_001005954 | zgc:103627 | **-2.31** | 9.45E-12 |
| Dr.104949 |  | TC293853 | LOC570293: Similar to Cytochrome b5 type A (microsomal) | **-2.31** | 1.77E-11 |
| Dr.30332 | Dr.30332 | NM_213644 | rpl7 | **-2.27** | 0.00E+00 |
| Dr.1152 | Dr.31534 | CN020033 | slc35e1 | **-2.26** | 6.33E-06 |
| Dr.16073 |  | NM_001020571 | zgc:110194 | **-2.26** | 3.85E-11 |
| Dr.483 | Dr.483 | NM_130949 | foxa2 | **-2.25** | 2.32E-08 |
| Dr.32854 | Dr.1126 | NM_213279 | rps2 | **-2.21** | 0.00E+00 |
| Dr.4758 | Dr.35218 | AI396697 | wu:fb09a01 | **-2.19** | 0.00E+00 |
| Dr.15984 |  | NM_001017793 | zgc:112426 | **-2.17** | 6.14E-33 |
| Dr.84440 | Dr.16762 | BC091658 | zgc:113055 | **-2.16** | 7.47E-17 |
| Dr.75580 | Dr.1455 | NM_200737 | fau | **-2.16** | 2.10E-22 |
| Dr.78416 |  | NM_001076560 | zgc:153632 | **-2.16** | 1.23E-14 |
| Dr.77675 | Dr.10202 | NM_131587 | e4tf | **-2.14** | 1.99E-40 |
| Dr.78227 | Dr.17252 | NM_200321 | zgc:56258 | **-2.11** | 5.57E-17 |
| Dr.59019 | Dr.1161 | NM_131620 | ybx1 | **-2.11** | 5.40E-06 |
| Dr.36406 | Dr.5515 | NM_214812 | zgc:85963 | **-2.09** | 0.00E+00 |
| Dr.75945 | Dr.24686 | NM_201584 | rpl12 | **-2.08** | 0.00E+00 |
| Dr.26403 | Dr.32395 | AI330514 | rps4x | **-2.08** | 1.00E-19 |
| Dr.30332 | Dr.30332 | NM_213644 | rpl7 | **-2.08** | 0.00E+00 |
| Dr.76433 | Dr.23552 | BC076307 | zgc:109898 | **-2.06** | 2.15E-20 |
| Dr.51292 | Dr.24851 | NM_200905 | zgc:76878 | **-2.06** | 5.56E-26 |
| Dr.80088 |  | NM_001044832 | si:ch211-266o5.1 | **-2.04** | 0.00E+00 |
| Dr.81708 | Dr.10341 | NM_131511 | fzd9 | **-2.04** | 9.94E-13 |
| Dr.75118 | Dr.34684 | CO935218 | zgc:112098 | **-2.04** | 1.53E-22 |
| Dr.10477 | Dr.10477 | NM_199568 | zgc:56334 | **-2.03** | 0.00E+00 |
| Dr.75674 | Dr.17536 | NM_198140 | tctp | **-2.02** | 1.46E-42 |
| Dr.31075 | Dr.29117 | NM_001002079 | zgc:91809 | **-2.01** | 0.00E+00 |
| Dr.79574 | Dr.5645 | NM_001007380 | si:dkey-12h9.10 | -1.99 | 7.78E-09 |
| Dr.75615 |  | TC290859 | eef1d: Elongation factor-1, delta | -1.98 | 1.67E-40 |
| Dr.18403 | Dr.18403 | NM_213456 | zgc:55572 | -1.98 | 1.12E-13 |
| Dr.78934 | Dr.37676 | CO812371 | zgc:92226 | -1.97 | 5.62E-28 |
| Dr.45282 |  | BC092946 | capns1 | -1.97 | 5.09E-33 |
| Dr.11174 | Dr.42739 | CN509271 | wu:fb76b12 | -1.95 | 8.25E-29 |
| Dr.75910 | Dr.749 | NM_131230 | tcp1 | -1.93 | 0.00E+00 |
| Dr.25810 | Dr.25810 | NM_212907 | foxq1 | -1.92 | 3.28E-07 |
| Dr.75142 | Dr.323 | AI959162 | si:ch211-197g15.1 | -1.92 | 0.00E+00 |
| Dr.1778 | Dr.1778 | NM_212691 | sdcbp | -1.92 | 1.54E-26 |
| Dr.80327 | Dr.23198 | NM_199922 | zgc:63796 | -1.91 | 6.63E-31 |
| Dr.80620 | Dr.17507 | NM_213505 | pob | -1.91 | 1.62E-18 |
| Dr.33503 | Dr.30841 | NM_001001811 | sox3 | -1.91 | 3.88E-30 |
| Dr.76746 | Dr.33561 | NM_001004601 | zgc:86878 | -1.89 | 1.68E-11 |
| Dr.47556 | Dr.38330 | CN015613 | zgc:109888 | -1.87 | 1.98E-20 |
| Dr.75774 | Dr.554 | NM_131224 | qk | -1.87 | 6.10E-22 |
| Dr.4035 | Dr.4035 | AI545063 | sb:cb793 | -1.87 | 1.34E-10 |
| Dr.75124 |  | NM_212617 | yy1 | -1.87 | 1.89E-06 |
| Dr.75910 | Dr.749 | NM_131230 | tcp1 | -1.86 | 4.42E-42 |
| Dr.85766 | Dr.18052 | AL718997 | znf593 | -1.86 | 1.29E-13 |
| Dr.75774 | Dr.554 | NM_131224 | qk | -1.85 | 1.04E-12 |
| Dr.28420 | Dr.28420 | NM_212612 | ddx5 | -1.85 | 8.81E-09 |
| Dr.26403 | Dr.47529 | AI617843 | hm:zehn2185 | -1.85 | 1.10E-19 |
| Dr.7743 | Dr.7743 | NM_131502 | foxh1 | -1.85 | 4.61E-06 |
| Dr.75452 | Dr.20194 | NM_213531 | zgc:55311 | -1.84 | 1.10E-09 |
| Dr.84984 |  | NM_001017600 | zgc:110289 | -1.82 | 5.22E-21 |
| Dr.35688 | Dr.31066 | BC065359 | hsp90b | -1.82 | 2.81E-29 |
| Dr.48738 | Dr.31614 | NM_001003447 | zgc:92114 | -1.79 | 0.00E+00 |
| - |  | AL596022 | itpka | -1.77 | 8.33E-07 |
| Dr.77021 | Dr.4159 | NM_001002116 | luc7l | -1.77 | 0.00E+00 |
| Dr.46820 |  | NM_131734 | gstp1 | -1.77 | 5.06E-42 |
| Dr.31853 | Dr.31853 | NM_001003840 | ebna1bp2l | -1.76 | 2.59E-42 |
| Dr.19274 |  | NM_173258 | ddx19 | -1.73 | 1.57E-16 |
| Dr.31752 | Dr.29423 | NM_173236 | hdac1 | -1.72 | 1.18E-23 |
| Dr.76638 |  | TC280178 | zgc:77282 | -1.72 | 1.26E-09 |
| Dr.105738 | Dr.1019 | BM776286 | wu:fi38h09 | -1.70 | 1.43E-13 |
| Dr.105320 |  | AI793637 | wu:fb95e12 | -1.69 | 3.19E-17 |
| Dr.36406 |  | NM_214812 | zgc:85963 | -1.69 | 1.63E-10 |
| Dr.24562 | Dr.24562 | NM_201325 | mvp | -1.69 | 0.00E+00 |
| Dr.31536 | Dr.31536 | NM_001003865 | rcl1 | -1.67 | 5.42E-24 |
| Dr.79159 | Dr.11443 | NM_213440 | plrg1 | -1.67 | 6.13E-38 |
| Dr.83713 |  | NM_001030276 | rrp12 | -1.66 | 1.15E-20 |
| Dr.77776 |  | CK685469 | si:ch211-59d15.5 | -1.65 | 0.00E+00 |
| Dr.907 | Dr.907 | NM_200059 | rps3a | -1.63 | 2.04E-16 |
| Dr.20919 | Dr.20919 | NM_199701 | sephs1 | -1.63 | 0.00E+00 |
| Dr.78308 | Dr.36312 | AI958380 | narg1a | -1.63 | 4.36E-25 |
| Dr.83914 | Dr.37941 | NM_001012496 | zgc:110738 | -1.60 | 2.33E-24 |
| Dr.1152 |  | NM_213074 | slc35e1 | -1.60 | 3.78E-11 |
| Dr.32149 |  | AY648823 | sf3b1 | -1.59 | 1.44E-18 |
| Dr.32607 |  | NM_213278 | sfpq | -1.59 | 2.42E-43 |
| Dr.82894 | Dr.11192 | NM_200735 | vamp4 | 1.59 | 2.66E-34 |
| Dr.75286 |  | NM_001037667 | ckap5 | 1.59 | 1.20E-42 |
| Dr.2951 | Dr.2951 | NM_001001589 | uqcrc2 | 1.59 | 0.00E+00 |
| Dr.81815 | Dr.12316 | NM_001002096 | yipf6 | 1.61 | 6.41E-17 |
| Dr.32677 | Dr.10563 | NM_212796 | msi2 | 1.61 | 0.00E+00 |
| Dr.83960 | Dr.13143 | BI883910 | zgc:154039 | 1.62 | 5.35E-14 |
| Dr.4565 | Dr.4565 | CO248858 | wu:fc62b10 | 1.62 | 1.29E-09 |
| Dr.78759 |  | NM_001007409 | zgc:101602 | 1.62 | 7.33E-36 |
| Dr.78532 |  | CD777567 | polr3b | 1.62 | 0.00E+00 |
| Dr.87043 | Dr.19087 | NM_001003651 | zgc:100786 | 1.63 | 7.93E-16 |
| Dr.13632 |  | AI385031 | wu:fb14c10 | 1.65 | 2.77E-36 |
| Dr.83731 | Dr.19531 | NM_200147 | wdr39 | 1.65 | 1.03E-17 |
| Dr.77826 | Dr.3099 | NM_199643 | coro2a | 1.66 | 0.00E+00 |
| Dr.86387 |  | TC273362 | zgc:158388 | 1.67 | 6.02E-37 |
| Dr.77928 |  | NM_199657 | scyl3 | 1.68 | 0.00E+00 |
| Dr.76999 | Dr.19560 | NM_199869 | insig1 | 1.68 | 1.30E-12 |
| Dr.14855 |  | NM_001003548 | zgc:100812 | 1.68 | 5.77E-22 |
| Dr.15396 |  | NM_001017765 | zgc:112080 | 1.68 | 4.24E-06 |
| Dr.89698 | Dr.30199 | NM_212814 | myd88 | 1.68 | 2.02E-22 |
| Dr.76129 | Dr.25678 | NM_214723 | eno3 | 1.69 | 2.41E-20 |
| Dr.6496 | Dr.6496 | NM_131585 | fth1 | 1.69 | 1.12E-23 |
| Dr.84157 |  | BC116518 | zgc:136362 | 1.70 | 1.80E-19 |
| Dr.20777 |  | NM_199832 | tk1 | 1.70 | 1.12E-27 |
| Dr.83627 | Dr.36811 | NM_001006002 | galk1 | 1.72 | 1.33E-24 |
| Dr.83033 | Dr.12645 | NM_001009908 | birc4 | 1.73 | 7.96E-21 |
| Dr.23596 | Dr.23596 | NM_001007435 | zgc:101668 | 1.74 | 1.91E-06 |
| Dr.76987 | Dr.20290 | NM_001007297 | ace2 | 1.74 | 1.01E-11 |
| Dr.78556 |  | NM_001030066 | wu:fc43a05 | 1.74 | 1.26E-14 |
| Dr.1791 | Dr.1791 | NM_200210 | pdap1 | 1.75 | 4.55E-07 |
| Dr.508 | Dr.29074 | NM_131833 | ube2i2 | 1.75 | 2.02E-36 |
| Dr.86785 | Dr.26635 | NM_200444 | zgc:63672 | 1.76 | 0.00E+00 |
| Dr.84112 | Dr.25490 | NM_200385 | zgc:64162 | 1.76 | 0.00E+00 |
| Dr.78759 | Dr.20362 | NM_001007409 | zgc:101602 | 1.77 | 2.76E-08 |
| Dr.83778 | Dr.12711 | NM_200437 | zgc:63572 | 1.77 | 3.16E-11 |
| Dr.17244 | Dr.17244 | NM_201070 | zgc:56533 | 1.78 | 2.01E-23 |
| Dr.80262 | Dr.17303 | BC055526 | chic1 | 1.78 | 0.00E+00 |
| Dr.81060 | Dr.7829 | NM_200695 | zgc:73056 | 1.79 | 4.58E-20 |
| Dr.79166 |  | NM_001044909 | si:ch211-222e23.1 | 1.79 | 2.32E-07 |
| Dr.78552 | Dr.42659 | CF550650 | wu:fd16a12 | 1.79 | 3.97E-06 |
| Dr.77335 |  | CF924907 | wu:fb70a09 | 1.80 | 4.25E-25 |
| Dr.17858 | Dr.17858 | NM_213469 | zgc:55521 | 1.80 | 4.14E-24 |
| Dr.132985 | Dr.10294 | BE016060 | LOC560147: Similar to KIAA0542 protein | 1.80 | 1.48E-13 |
| Dr.80398 |  | NM_001077338 | zgc:153079 | 1.80 | 3.52E-20 |
| Dr.81600 |  | NM_001077141 | zgc:154057 | 1.81 | 0.00E+00 |
| Dr.75408 | Dr.90 | NM_212878 | zgc:76925 | 1.81 | 9.26E-09 |
| Dr.84744 | Dr.16550 | NM_001002640 | zgc:92201 | 1.82 | 1.55E-30 |
| Dr.16573 | Dr.16573 | BC067648 | zgc:112524 | 1.82 | 5.29E-06 |
| Dr.18294 | Dr.18294 | NM_153656 | ptgs1 | 1.82 | 8.75E-08 |
| Dr.7685 | Dr.7685 | NM_199432 | zgc:55819 | 1.82 | 3.41E-17 |
| Dr.43246 | Dr.10424 | CO248295 | wu:fl22g01 | 1.82 | 2.08E-29 |
| Dr.22387 |  | NM_200403 | tmed5 | 1.83 | 1.15E-25 |
| Dr.76154 | Dr.13623 | NM_207074 | zgc:77241 | 1.83 | 0.00E+00 |
| Dr.76778 | Dr.3466 | NM_205559 | zgc:77292 | 1.84 | 1.37E-20 |
| Dr.84449 | Dr.16511 | NM_199217 | fhl | 1.84 | 2.21E-30 |
| Dr.82387 | Dr.27053 | NM_194399 | cflar | 1.84 | 3.85E-13 |
| Dr.45832 |  | NM_001024426 | zgc:110022 | 1.85 | 0.00E+00 |
| Dr.20961 | Dr.20961 | NM_131295 | cpla2 | 1.85 | 1.77E-09 |
| Dr.84280 | Dr.24974 | NM_200313 | chd1l | 1.85 | 8.33E-21 |
| Dr.256 | Dr.256 | NM_201577 | sec61b | 1.85 | 1.16E-36 |
| Dr.78550 | Dr.15607 | CK697115 | wu:fd15h01 | 1.86 | 5.40E-12 |
| Dr.67202 | Dr.11928 | NM_200874 | arhgap12 | 1.86 | 8.53E-21 |
| Dr.88238 | Dr.27178 | NM_205611 | NIT2 | 1.86 | 0.00E+00 |
| Dr.23391 | Dr.7337 | NM_212708 | slc16a3 | 1.87 | 1.34E-38 |
| Dr.119699 |  | TC268843 | LOC796968: Hypothetical protein LOC796968 | 1.87 | 6.47E-33 |
| Dr.80562 | Dr.38332 | BC090902 | zgc:103571 | 1.88 | 0.00E+00 |
| Dr.18993 | Dr.18993 | NM_205595 | zgc:103496 | 1.88 | 1.58E-32 |
| Dr.84969 |  | NM_001017809 | zgc:110323 | 1.89 | 0.00E+00 |
| Dr.76565 | Dr.4897 | NM_001005939 | mcfd2 | 1.89 | 6.97E-14 |
| Dr.8101 | Dr.8101 | NM_131249 | evx1 | 1.89 | 0.00E+00 |
| Dr.82475 | Dr.16793 | NM_001002522 | zgc:92803 | 1.89 | 0.00E+00 |
| Dr.2761 | Dr.2761 | NM_200096 | tbp | 1.90 | 0.00E+00 |
| Dr.87440 | Dr.30417 | NM_212800 | disp1 | 1.90 | 5.98E-36 |
| Dr.37005 | Dr.37005 | BC091559 | zgc:114132 | 1.90 | 7.82E-43 |
| Dr.75324 | Dr.32136 | AI477125 | pola1 | 1.90 | 0.00E+00 |
| Dr.87058 | Dr.18809 | NM_001008621 | zgc:101800 | 1.90 | 1.02E-07 |
| Dr.2251 | Dr.2251 | CK029119 | golph4 | 1.91 | 1.34E-26 |
| Dr.77414 | Dr.3637 | NM_200122 | tubgcp2 | 1.91 | 7.93E-18 |
| Dr.82958 |  | NM_001020766 | zgc:112343 | 1.91 | 2.01E-21 |
| Dr.84895 | Dr.16631 | NM_001003557 | zgc:101119 | 1.91 | 0.00E+00 |
| Dr.38137 | Dr.7345 | BC090303 | zgc:113424 | 1.91 | 2.99E-40 |
| Dr.83238 |  | NM_001017763 | zgc:112084 | 1.92 | 8.54E-40 |
| Dr.82671 | Dr.12741 | CK027466 | LOC567716: Hypothetical LOC567716 | 1.92 | 2.24E-11 |
| Dr.81740 | Dr.12488 | NM_199539 | zgc:56200 | 1.92 | 3.94E-23 |
| Dr.51770 | Dr.1193 | NM_001006069 | nudt22 | 1.92 | 3.70E-13 |
| Dr.86868 |  | NM_212884 | zgc:77065 | 1.93 | 0.00E+00 |
| Dr.16249 | Dr.16249 | NM_200164 | slc25a14 | 1.93 | 3.99E-24 |
| Dr.80291 | Dr.12730 | NM_200438 | zgc:63574 | 1.93 | 6.85E-06 |
| Dr.77320 | Dr.29013 | NM_206829 | zgc:77126 | 1.94 | 6.43E-37 |
| Dr.43565 |  | NM_001045358 | zgc:153664 | 1.94 | 5.48E-40 |
| Dr.86401 | Dr.27099 | NM_200637 | adam8 | 1.94 | 0.00E+00 |
| Dr.77330 | Dr.4249 | NM_213404 | lipf | 1.94 | 1.54E-44 |
| Dr.76004 | Dr.8212 | NM_131391 | psmb9a | 1.94 | 2.51E-06 |
| Dr.117725 |  | XP_688896 | LOC560402: Hypothetical LOC560402 | 1.94 | 2.94E-19 |
| Dr.80473 | Dr.15169 | NM_201304 | rgs14 | 1.95 | 3.15E-10 |
| Dr.31168 | Dr.28835 | NM_213045 | zgc:73358 | 1.96 | 1.85E-34 |
| Dr.82742 | Dr.24315 | NM_200813 | ca7 | 1.96 | 2.47E-09 |
| Dr.75962 | Dr.13089 | NM_001007200 | ptpn4 | 1.96 | 5.69E-15 |
| Dr.123566 |  | BI326808 | rock2b | 1.97 | 3.24E-11 |
| Dr.72337 | Dr.11561 | CA496316 | zgc:152651 | 1.97 | 0.00E+00 |
| Dr.76167 |  | NM_200540 | snx27 | 1.97 | 2.10E-11 |
| Dr.86558 |  | NM_001004670 | lypd6 | 1.97 | 1.93E-09 |
| Dr.8136 | Dr.8136 | NM_131377 | pthr2 | 1.98 | 2.55E-09 |
| Dr.41494 |  | XP_697409 | LOC568957: Hypothetical LOC568957 | 1.98 | 3.93E-14 |
| Dr.8283 | Dr.8283 | NM_131693 | mibp | 1.98 | 1.74E-34 |
| Dr.134371 |  | XP_693285 | Transcribed locus, weakly similar to scramblase 1 | 1.99 | 1.37E-26 |
| Dr.8290 | Dr.8290 | NM_130924 | ptp1b | 1.99 | 2.00E-26 |
| Dr.35143 |  | BC095563 | zgc:111826 | 1.99 | 1.32E-20 |
| Dr.82471 | Dr.11543 | NM_001006022 | zgc:103482 | 1.99 | 0.00E+00 |
| Dr.79317 | Dr.31508 | NM_001002360 | zgc:92520 | **2.00** | 1.33E-10 |
| Dr.3675 | Dr.3675 | CN503149 | wu:fb94a02 | **2.01** | 2.40E-16 |
| Dr.16163 | Dr.16163 | NM_205626 | bcor | **2.02** | 1.19E-16 |
| Dr.75078 | Dr.14 | NM_131113 | pouc | **2.02** | 4.04E-13 |
| Dr.20705 | Dr.20705 | NM_205714 | zgc:103537 | **2.02** | 1.94E-29 |
| Dr.76493 | Dr.3615 | NM_213250 | csnk1d | **2.02** | 1.82E-33 |
| Dr.75660 |  | NM_001013541 | zgc:101608 | **2.02** | 0.00E+00 |
| Dr.37814 | Dr.37814 | NM_001008655 | zgc:101530 | **2.03** | 3.79E-18 |
| Dr.30522 | Dr.30522 | NM_205759 | zgc:77395 | **2.03** | 1.38E-08 |
| Dr.85007 |  | NM_001077149 | zgc:152949 | **2.03** | 1.98E-11 |
| Dr.78839 |  | NM_001020605 | zgc:110584 | **2.03** | 5.61E-44 |
| Dr.4883 | Dr.4883 | NM_200136 | hsd17b4 | **2.04** | 0.00E+00 |
| Dr.28706 | Dr.8682 | NM_199705 | rbm4 | **2.04** | 1.95E-37 |
| Dr.79749 | Dr.4685 | NM_001007317 | zgc:92177 | **2.04** | 0.00E+00 |
| Dr.80452 | Dr.11332 | NM_200670 | phactr4 | **2.04** | 0.00E+00 |
| Dr.78260 | Dr.16048 | NM_200172 | c-mycb | **2.04** | 3.89E-39 |
| Dr.80588 |  | BC129167 | zgc:158232 | **2.04** | 9.25E-11 |
| Dr.85592 | Dr.15993 | NM_001002415 | zgc:92676 | **2.05** | 1.39E-20 |
| Dr.78835 | Dr.14750 | NM_001003866 | gtf3a | **2.05** | 0.00E+00 |
| Dr.60628 |  | BC096971 | wu:fi34e04 | **2.05** | 9.89E-08 |
| Dr.48619 |  | NM_001034982 | zgc:110032 | **2.05** | 4.09E-08 |
| Dr.67791 | Dr.26116 | NM_213058 | hspa5 | **2.05** | 0.00E+00 |
| Dr.76728 | Dr.4767 | BC056540 | tmed3 | **2.06** | 2.10E-11 |
| Dr.9621 | Dr.9621 | NM_200758 | zgc:73238 | **2.06** | 1.20E-08 |
| Dr.75218 | Dr.30431 | NM_001001822 | ptenb | **2.06** | 2.73E-18 |
| Dr.79966 | Dr.7531 | NM_213193 | zgc:77862 | **2.07** | 1.91E-13 |
| Dr.18291 | Dr.18291 | AF411390 | bivm | **2.07** | 1.90E-33 |
| Dr.79235 | Dr.15700 | NM_199707 | ell | **2.08** | 0.00E+00 |
| Dr.77199 | Dr.12605 | NM_212792 | cdkn1b / p27, kip1 | **2.09** | 0.00E+00 |
| Dr.90256 | Dr.36432 | NM_001004611 | zgc:103507 | **2.10** | 5.87E-07 |
| Dr.10580 | Dr.10580 | NM_201050 | nek2 | **2.10** | 0.00E+00 |
| Dr.78921 |  | NM_001077330 | zgc:153420 | **2.10** | 0.00E+00 |
| Dr.120502 |  | AI878055.1 | LOC557002: Similar to X-MyT1 | **2.11** | 1.84E-20 |
| Dr.75708 | Dr.4453 | NM_212709 | ppp1r3b | **2.12** | 0.00E+00 |
| Dr.29104 | Dr.29104 | CO247502 | fig1a | **2.12** | 7.18E-27 |
| Dr.692 | Dr.692 | NM_131796 | ptk2.1 | **2.13** | 0.00E+00 |
| Dr.133813 |  | BM860048 | LOC566907: Similar to interferon-inducible protein Gig2 | **2.14** | 4.85E-12 |
| Dr.77864 | Dr.4649 | NM_152958 | phc2 | **2.14** | 2.44E-17 |
| Dr.91802 | Dr.30565 | NM_213547 | zC4ST-2 | **2.14** | 4.41E-10 |
| Dr.18625 | Dr.18625 | NM_001002313 | ift81 | **2.14** | 1.36E-20 |
| Dr.32515 | Dr.5859 | NM_213227 | usp33 | **2.14** | 0.00E+00 |
| Dr.9520 | Dr.9520 | CN508052 | nfatc2ip | **2.14** | 3.28E-14 |
| Dr.87503 | Dr.36503 | NM_001004648 | zgc:101062 | **2.16** | 3.75E-09 |
| Dr.32551 | Dr.27494 | NM_200565 | ccdc124 | **2.16** | 0.00E+00 |
| Dr.15663 |  | NM_131886 | cebpg | **2.17** | 2.37E-10 |
| Dr.83266 | Dr.17188 | NM_201069 | zgc:56164 | **2.17** | 0.00E+00 |
| Dr.31168 | Dr.28835 | NM_213045 | zgc:73358 | **2.17** | 1.21E-32 |
| Dr.77100 | Dr.3562 | BC091455 | zgc:110308 | **2.17** | 0.00E+00 |
| Dr.83956 | Dr.15179 | NM_212932 | zgc:85752 | **2.18** | 0.00E+00 |
| Dr.92224 |  | NM_001002044 | mafk | **2.18** | 2.65E-06 |
| Dr.84295 | Dr.15822 | NM_200389 | zgc:64174 | **2.18** | 9.12E-23 |
| Dr.75085 | Dr.37 | NM_131150 | pou12 | **2.18** | 5.92E-12 |
| Dr.85496 | Dr.30951 | NM_212982 | fancl | **2.18** | 0.00E+00 |
| Dr.82352 | Dr.15941 | NM_201003 | zgc:63557 | **2.19** | 7.27E-23 |
| Dr.14671 | Dr.14671 | BC056739 | birc5a | **2.19** | 4.93E-25 |
| Dr.82365 | Dr.16134 | NM_001003415 | pdcd7 | **2.19** | 8.39E-22 |
| Dr.36953 | Dr.21263 | NM_001006088 | asah1 | **2.20** | 0.00E+00 |
| Dr.16863 | Dr.16863 | NM_001007376 | zgc:101877 | **2.20** | 0.00E+00 |
| Dr.79739 |  | NM_001017882 | sar1a | **2.21** | 1.97E-18 |
| Dr.1496 | Dr.1496 | NM_200022 | apg3l | **2.21** | 0.00E+00 |
| Dr.75429 | Dr.26412 | NM_001007447 | cpt2 | **2.22** | 2.75E-16 |
| Dr.75493 | Dr.207 | NM_214694 | zgc:55781 | **2.22** | 0.00E+00 |
| Dr.16840 | Dr.16840 | NM_001006031 | zgc:103457 | **2.22** | 3.93E-18 |
| Dr.76644 | Dr.1915 | NM_214770 | zgc:55760 | **2.23** | 0.00E+00 |
| Dr.78179 | Dr.13050 | CN022313 | zgc:110459 | **2.23** | 6.77E-16 |
| Dr.74191 |  | BC074077 | twf1l | **2.24** | 1.99E-40 |
| Dr.37831 | Dr.37831 | NM_001008597 | zgc:103611 | **2.24** | 1.14E-08 |
| Dr.84518 | Dr.13672 | NM_001002140 | gtpbpl | **2.25** | 0.00E+00 |
| Dr.85981 | Dr.26701 | NM_207640 | ifn | **2.25** | 5.65E-13 |
| Dr.86046 | Dr.16669 | NM_200592 | nr2f1l | **2.25** | 0.00E+00 |
| Dr.83481 | Dr.18073 | NM_001002196 | zgc:91853 | **2.25** | 1.92E-43 |
| Dr.85603 |  | NM_001004683 | mrpl17 | **2.25** | 3.88E-14 |
| Dr.40212 |  | BM958233 | LOC796750: Similar to LOC495955 protein | **2.25** | 1.48E-34 |
| Dr.81636 | Dr.21046 | NM_200160 | zgc:55511 | **2.25** | 1.36E-19 |
| Dr.84847 | Dr.34247 | NM_001003996 | zgc:92404 | **2.25** | 8.02E-17 |
| Dr.75493 | Dr.207 | NM_214694 | zgc:55781 | **2.26** | 0.00E+00 |
| Dr.85139 | Dr.31819 | NM_001002433 | zgc:92648 | **2.26** | 6.36E-22 |
| Dr.78748 | Dr.7554 | NM_001006021 | amt | **2.27** | 1.35E-06 |
| Dr.78661 | Dr.5588 | NM_214716 | hspa4l | **2.28** | 3.97E-13 |
| Dr.37880 | Dr.33206 | NM_001008577 | fbxo44 | **2.28** | 0.00E+00 |
| Dr.80366 | Dr.9538 | NM_200997 | zgc:55283 | **2.29** | 0.00E+00 |
| Dr.811 | Dr.811 | NM_200073 | rps6kal | **2.29** | 1.31E-19 |
| Dr.36500 | Dr.36500 | NM_001005575 | bcl9l | **2.29** | 7.42E-08 |
| Dr.80846 | Dr.16014 | NM_001007372 | zgc:101897 | **2.29** | 4.00E-11 |
| Dr.133023 |  | BC124781 | zgc:153972 | **2.29** | 3.17E-10 |
| Dr.81341 | Dr.9471 | BC057503 | c1qdc1 | **2.29** | 0.00E+00 |
| Dr.85603 | Dr.16017 | NM_001004683 | mrpl17 | **2.30** | 8.66E-29 |
| Dr.37198 | Dr.37198 | NM_001007439 | zgc:101659 | **2.31** | 8.02E-11 |
| Dr.31877 | Dr.12453 | NM_001002684 | zgc:86764 | **2.31** | 0.00E+00 |
| Dr.85875 | Dr.19081 | NM_001003639 | zgc:100914 | **2.31** | 1.32E-32 |
| Dr.89808 | Dr.30427 | NM_212861 | fibpl | **2.31** | 3.12E-15 |
| Dr.85291 |  | TC298366 | LOC566605: Similar to serine/threonine kinase 23 | **2.31** | 2.02E-09 |
| Dr.16862 | Dr.16862 | NM_001003887 | LOC445411: RIKEN cDNA 5730590G19-like | **2.32** | 8.87E-08 |
| Dr.75698 | Dr.14267 | NM_201202 | zgc:56685 | **2.32** | 6.68E-37 |
| Dr.84024 | Dr.14835 | CO351115 | Phf3 | **2.33** | 9.45E-25 |
| - |  | TC283756 | unknown | **2.33** | 6.35E-09 |
| Dr.82493 | Dr.26629 | NM_200492 | tmem68 | **2.33** | 5.16E-21 |
| Dr.80525 | Dr.5969 | NM_200324 | zgc:56402 | **2.34** | 6.03E-44 |
| Dr.10637 |  | NM_001001944 | xrn2 | **2.34** | 3.18E-24 |
| Dr.79030 |  | AI793871 | wu:fc55d08 | **2.34** | 1.16E-22 |
| Dr.80580 | Dr.7310 | NM_199430 | ccnb2 | **2.35** | 0.00E+00 |
| Dr.9751 | Dr.9751 | NM_198910 | fmo5 | **2.35** | 4.54E-17 |
| Dr.21599 | Dr.21599 | NM_212672 | atp6v0a1 | **2.37** | 4.20E-10 |
| Dr.14969 | Dr.14969 | NM_001005982 | cln6 | **2.37** | 2.29E-09 |
| Dr.89721 | Dr.7073 | BC059636 | BC059636 | **2.37** | 2.80E-10 |
| Dr.85343 | Dr.27152 | NM_200799 | zgc:73349 | **2.37** | 7.44E-06 |
| Dr.80392 | Dr.23975 | NM_199798 | zgc:63471 | **2.37** | 8.81E-06 |
| Dr.109269 | Dr.37477 | CK679749 | LOC792787: Similar to LOC443692 protein | **2.38** | 2.52E-25 |
| Dr.79807 |  | NM_001039637 | foxp1 | **2.38** | 1.61E-07 |
| Dr.77494 | Dr.2062 | NM_213385 | vrk1 | **2.39** | 9.77E-22 |
| Dr.13664 |  | AI722610 | si:zfos-47c12.1 | **2.39** | 0.00E+00 |
| Dr.2933 | Dr.2933 | NM_214685 | laptm4a | **2.39** | 0.00E+00 |
| Dr.82468 | Dr.11538 | NM_200491 | tbc1d19 | **2.40** | 2.01E-23 |
| Dr.72349 |  | NM_001077367 | shd | **2.41** | 1.27E-35 |
| Dr.78163 |  | BI896325 | LOC100000660: Similar to PPI domain and WD rep. cont.1 | **2.41** | 3.64E-32 |
| Dr.81554 | Dr.23018 | NM_001004560 | zgc:92250 | **2.42** | 2.40E-33 |
| Dr.9240 | Dr.26619 | NM_201135 | atp6v1al | **2.42** | 5.55E-31 |
| Dr.77521 | Dr.26773 | NM_182855 | tgfbr2 | **2.42** | 0.00E+00 |
| Dr.46022 | Dr.46022 | AW128831 | tpte | **2.42** | 1.02E-06 |
| Dr.79115 | Dr.18740 | NM_199698 | sec22a | **2.43** | 0.00E+00 |
| Dr.78102 | Dr.6191 | NM_131539 | pim1 | **2.43** | 1.15E-09 |
| Dr.7638 | Dr.7638 | NM_182967 | calm3a | **2.43** | 1.53E-26 |
| Dr.78131 |  | AY584746 | lin7b | **2.43** | 5.46E-07 |
| Dr.4660 | Dr.4660 | NM_212632 | zgc:56344 | **2.43** | 0.00E+00 |
| Dr.82645 | Dr.13658 | NM_001002526 | atp6v1f | **2.44** | 1.53E-18 |
| Dr.76033 |  | NM_213387 | pgk1 | **2.44** | 0.00E+00 |
| - |  | XP_692617.1 | LOC564176 similar to LOC496068 protein | **2.44** | 1.26E-24 |
| Dr.77965 |  | NM_001017669 | zgc:112480 | **2.44** | 1.40E-12 |
| Dr.77003 |  | AI477804 | ppargc1b | **2.44** | 3.33E-17 |
| Dr.34870 | Dr.32049 | NM_001002498 | hccsa | **2.44** | 0.00E+00 |
| Dr.77415 | Dr.14973 | NM_199630 | stx5al | **2.45** | 4.97E-25 |
| Dr.32734 |  | NM_212605 | ywhae1 | **2.45** | 2.54E-21 |
| Dr.3370 | Dr.32182 | NM_001002179 | zgc:91908 | **2.45** | 2.80E-45 |
| Dr.3200 |  | AI721919 | dapk3 | **2.45** | 0.00E+00 |
| Dr.77301 |  | NM_199615 | serhl | **2.45** | 1.77E-17 |
| Dr.81055 | Dr.6460 | NM_001002707 | zgc:92606 | **2.45** | 2.75E-11 |
| Dr.86390 | Dr.17466 | NM_200442 | zgc:63666 | **2.47** | 0.00E+00 |
| Dr.84111 |  | NM_200263 | zgc:56450 | **2.47** | 8.98E-43 |
| Dr.73987 | Dr.31642 | BC090737 | arl6ip2 | **2.47** | 0.00E+00 |
| Dr.6104 | Dr.6104 | NM_199480 | lnp | **2.47** | 1.61E-32 |
| Dr.81242 | Dr.13477 | NM_001008586 | zgc:103660 | **2.47** | 1.10E-36 |
| Dr.7421 |  | NM_173270 | atp6v1h | **2.48** | 0.00E+00 |
| Dr.82600 | Dr.10682 | AF246175 | trac | **2.49** | 1.06E-12 |
| Dr.34241 | Dr.34241 | NM_001003998 | zgc:100789 | **2.49** | 5.23E-36 |
| Dr.78711 | Dr.16391 | NM_200649 | acsl4 | **2.49** | 1.34E-17 |
| Dr.9174 | Dr.9174 | NM_200171 | zgc:55673 | **2.49** | 0.00E+00 |
| Dr.75838 | Dr.614 | NM_131427 | zorba | **2.49** | 0.00E+00 |
| Dr.82408 | Dr.17267 | CN015300 | pkig | **2.49** | 0.00E+00 |
| Dr.85111 | Dr.15393 | NM_001020566 | zgc:110130 | **2.50** | 7.87E-27 |
| Dr.75878 | Dr.11635 | NM_201206 | cox15 | **2.50** | 0.00E+00 |
| Dr.78102 | Dr.6191 | NM_131539 | pim1 | **2.51** | 2.30E-14 |
| Dr.28324 | Dr.28324 | NM_001007335 | zgc:103553 | **2.51** | 0.00E+00 |
| Dr.6680 | Dr.6680 | NM_212723 | zgc:55587 | **2.52** | 3.36E-44 |
| Dr.84960 |  | NM_001017810 | zgc:112417 | **2.52** | 2.18E-11 |
| Dr.81210 | Dr.12851 | NM_001004612 | zgc:103495 | **2.52** | 3.35E-17 |
| Dr.82081 |  | NM_212734 | zgc:77556 | **2.52** | 0.00E+00 |
| Dr.79799 |  | NM_001024439 | zgc:112262 | **2.53** | 0.00E+00 |
| Dr.82247 |  | XM_684648 | LOC561242: Similar to KIAA1946 | **2.54** | 0.00E+00 |
| Dr.83954 |  | NM_200657 | thyn1 | **2.54** | 5.87E-10 |
| - | Dr.15581 | BM316955 | fw77d06.y1 Gong zebrafish testis Danio rerio cDNA clone | **2.55** | 3.08E-44 |
| Dr.7322 |  | NM_001025460 | wu:fi35c01 | **2.56** | 4.69E-12 |
| Dr.75130 |  | NM_152982 | myhz2 | **2.56** | 0.00E+00 |
| Dr.77134 | Dr.1543 | NM_199610 | dnajc3 | **2.56** | 0.00E+00 |
| Dr.12908 | Dr.12908 | NM_200712 | zgc:73100 | **2.57** | 0.00E+00 |
| Dr.19791 |  | BC124181 | zgc:152930 | **2.58** | 0.00E+00 |
| Dr.79854 | Dr.37392 | CK678062 | im:7139082 | **2.58** | 4.89E-09 |
| Dr.83214 | Dr.17504 | NM_200394 | zgc:56405 | **2.58** | 0.00E+00 |
| Dr.77341 | Dr.3817 | NM_199779 | pdia4 | **2.59** | 5.25E-13 |
| Dr.31925 | Dr.465 | NM_153662 | siat8 | **2.59** | 6.06E-14 |
| Dr.85880 | Dr.34597 | NM_001007158 | nspc1 | **2.60** | 3.78E-22 |
| Dr.88733 | Dr.30832 | NM_001006662 | st6galnac5 | **2.60** | 9.46E-09 |
| Dr.75209 |  | BQ285121 | tpx2 | **2.60** | 0.00E+00 |
| Dr.4066 | Dr.4066 | NM_214708 | zgc:56558 | **2.60** | 4.76E-36 |
| Dr.86849 | Dr.26614 | NM_200483 | zgc:63690 | **2.61** | 8.41E-08 |
| Dr.77417 | Dr.28260 | NM_001003555 | zgc:101125 | **2.61** | 0.00E+00 |
| Dr.81990 |  | BC095746 | zgc:112307 | **2.62** | 1.21E-23 |
| Dr.78703 | Dr.18135 | NM_001005937 | plekhk1 | **2.62** | 3.63E-19 |
| Dr.86016 | Dr.17425 | CK677368 | im:7138279 | **2.62** | 3.13E-17 |
| Dr.88904 | Dr.6650 | NM_212806 | cyp17a1 | **2.62** | 0.00E+00 |
| Dr.14847 | Dr.14847 | NM_001005772 | atp6v1c1l | **2.62** | 0.00E+00 |
| Dr.89116 | Dr.17665 | NM_205614 | flj11011l | **2.63** | 0.00E+00 |
| Dr.76765 |  | TC291935 | zgc:153980 | **2.63** | 0.00E+00 |
| Dr.79216 |  | BM096039 | LOC797142: Similar to replication factor C large subunit | **2.64** | 3.07E-14 |
| Dr.86865 |  | NM_205745 | wdfy2 | **2.64** | 1.02E-11 |
| Dr.85181 | Dr.15530 | NM_001005938 | park7 | **2.64** | 3.27E-21 |
| Dr.4909 | Dr.4909 | AI545701 | wu:fb75c03 | **2.65** | 0.00E+00 |
| Dr.77685 | Dr.29163 | BC076285 | slc1a4 | **2.65** | 4.51E-15 |
| Dr.26788 | Dr.26788 | CD597695 | LOC793880: Hypothetical protein LOC793880 | **2.65** | 1.13E-10 |
| Dr.5370 |  | NM_001020715 | zgc:113169 | **2.65** | 7.52E-19 |
| Dr.2713 | Dr.10301 | NM_212663 | cd82 | **2.66** | 0.00E+00 |
| Dr.78299 |  | NM_199805 | phf17 | **2.66** | 1.55E-17 |
| Dr.81683 | Dr.20582 | NM_200259 | rbpms2 | **2.66** | 1.92E-37 |
| Dr.14401 | Dr.14401 | CF998588 | LOC558609: Hypothetical LOC558609 | **2.67** | 1.70E-40 |
| Dr.93989 |  | BC095713 | si:ch211-10e8.6 | **2.67** | 5.34E-41 |
| Dr.83056 | Dr.12218 | NM_200181 | sh3gl3 | **2.67** | 1.84E-15 |
| Dr.76723 | Dr.4082 | NM_001003499 | zgc:91985 | **2.67** | 9.55E-24 |
| Dr.8287 | Dr.8287 | NM_131862 | jag2 | **2.67** | 0.00E+00 |
| Dr.85322 | Dr.32125 | NM_001002565 | zgc:92758 | **2.67** | 6.64E-21 |
| Dr.79083 |  | NM_200319 | tmem57 | **2.68** | 3.54E-18 |
| Dr.81259 | Dr.8159 | NM_131524 | dazl | **2.68** | 3.72E-31 |
| Dr.82922 | Dr.12612 | NM_205713 | unc119.2 | **2.68** | 1.17E-37 |
| Dr.80706 |  | BE016318 | wu:fk71g01 | **2.68** | 0.00E+00 |
| Dr.37023 | Dr.37023 | NM_001006061 | zgc:101745 | **2.69** | 8.39E-07 |
| - |  | CN510175 | unknown | **2.69** | 8.33E-06 |
| Dr.82151 | Dr.20599 | NM_212736 | mecp2 | **2.69** | 0.00E+00 |
| Dr.9174 | Dr.9174 | NM_200171 | zgc:55673 | **2.71** | 0.00E+00 |
| Dr.89308 | Dr.37791 | NM_001009985 | zgc:92261 | **2.71** | 9.73E-23 |
| Dr.81505 | Dr.18799 | NM_213147 | acox3 | **2.74** | 1.01E-11 |
| Dr.37012 | Dr.37012 | NM_001007339 | zgc:103521 | **2.74** | 3.57E-09 |
| Dr.116279 |  | TC272282 | LOC795928: Hypothetical protein LOC795928 | **2.75** | 1.04E-22 |
| Dr.82979 | Dr.27160 | NM_200731 | zgc:73148 | **2.75** | 7.96E-20 |
| Dr.37880 | Dr.33206 | NM_001008577 | fbxo44 | **2.75** | 4.28E-16 |
| - |  | BC085675 | zgc:92577 | **2.75** | 0.00E+00 |
| Dr.75377 | Dr.53 | NM_201450 | cse1l | **2.76** | 2.43E-12 |
| Dr.75286 |  | NM_001037667 | ckap5 | **2.76** | 7.51E-09 |
| Dr.84713 |  | NM_001039932 | zgc:136926 | **2.76** | 1.99E-07 |
| Dr.83495 | Dr.14491 | BC090288 | LOC565764: Similar to Family w. sequence similarity 33-A | **2.77** | 0.00E+00 |
| Dr.32699 |  | CK683591 | im:7145864 | **2.77** | 4.19E-13 |
| Dr.28324 | Dr.28324 | NM_001007335 | zgc:103553 | **2.78** | 0.00E+00 |
| Dr.75976 | Dr.831 | NM_200446 | zgc:63792 | **2.78** | 0.00E+00 |
| Dr.78263 | Dr.8795 | NM_001008575 | slc30a9 | **2.79** | 0.00E+00 |
| Dr.15775 | Dr.15775 | NM_201001 | arl2bp | **2.79** | 3.71E-37 |
| Dr.83458 | Dr.14445 | NM_200798 | tmem126a | **2.79** | 5.02E-11 |
| Dr.76257 |  | NM_131579 | bad | **2.80** | 0.00E+00 |
| Dr.85970 | Dr.28789 | NM_001005988 | zgc:103539 | **2.80** | 1.94E-19 |
| Dr.46820 | Dr.23788 | NM_131734 | gstp1 | **2.81** | 1.24E-07 |
| Dr.105703 |  | AF097477 | LOC407635: Protein tyrosine phosphatase H1 | **2.81** | 7.39E-18 |
| Dr.27137 | Dr.27137 | NM_200729 | zgc:73144 | **2.82** | 0.00E+00 |
| Dr.78604 | Dr.20468 | NM_200431 | zgc:66488 | **2.83** | 2.80E-45 |
| Dr.7322 | Dr.7322 | CV483795 | wu:fi35c01 | **2.83** | 0.00E+00 |
| Dr.85181 | Dr.15530 | NM_001005938 | park7 | **2.83** | 2.49E-18 |
| Dr.21365 | Dr.8207 | NM_131810 | cx44.2 | **2.85** | 0.00E+00 |
| Dr.29876 | Dr.29876 | CK866461 | LOC565374: Hypothetical LOC565374 | **2.86** | 0.00E+00 |
| Dr.10562 | Dr.10562 | NM_212717 | olfm2 | **2.86** | 4.09E-25 |
| Dr.19063 | Dr.19063 | NM_200308 | zgc:56665 | **2.87** | 8.85E-34 |
| Dr.38226 |  | BC093325 | fbxo16 | **2.87** | 5.60E-15 |
| Dr.76909 | Dr.38065 | AI444417 | wu:fb38b11 | **2.88** | 1.35E-36 |
| Dr.32886 | Dr.19381 | NM_200907 | vps26 | **2.89** | 3.81E-07 |
| Dr.84666 | Dr.16139 | NM_205724 | zgc:100907 | **2.89** | 5.28E-30 |
| Dr.85404 |  | BX255917 | zbtb1 | **2.89** | 2.22E-17 |
| Dr.80415 | Dr.20729 | NM_213496 | prpsap2 | **2.91** | 0.00E+00 |
| Dr.91270 | Dr.30770 | CN506484 | Transcribed locus | **2.91** | 0.00E+00 |
| Dr.79940 | Dr.7747 | NM_200098 | laptm4b | **2.92** | 1.88E-40 |
| - |  | NW_634139 | LOC556527 similar to F-box and leucine-rich repeat protein 2 | **2.92** | 1.48E-08 |
| Dr.76315 | Dr.8587 | NM_173283 | igfbp1 | **2.92** | 1.79E-06 |
| Dr.78310 | Dr.3133 | NM_213097 | pla2g6 | **2.94** | 4.41E-15 |
| Dr.78610 | Dr.15098 | NM_199767 | slc26a11 | **2.95** | 0.00E+00 |
| Dr.76596 | Dr.2382 | NM_001003995 | rnuxa | **2.96** | 0.00E+00 |
| Dr.79937 | Dr.15406 | NM_200228 | pex3 | **2.96** | 6.01E-13 |
| Dr.91652 |  | NM_001037681 | tcf21 | **2.97** | 1.44E-10 |
| Dr.26623 | Dr.26623 | BC054933 | camsap1 | **2.97** | 0.00E+00 |
| Dr.81654 | Dr.27096 | BC057524 | LOC402861: Hypothetical protein LOC402861 | **2.98** | 0.00E+00 |
| Dr.78475 | Dr.29753 | NM_205693 | zgc:77891 | **2.98** | 0.00E+00 |
| Dr.77782 | Dr.7973 | NM_212643 | slc35c2 | **2.99** | 1.58E-17 |
| Dr.32367 | Dr.5605 | NM_198809 | tubb2c | **2.99** | 0.00E+00 |
| Dr.82922 |  | NM_205713 | unc119.2 | **3.00** | 0.00E+00 |
| Dr.76735 | Dr.7676 | NM_205650 | zgc:77759 | **3.00** | 4.18E-07 |
| Dr.76694 | Dr.28244 | NM_001001818 | gnai2l | **3.01** | 0.00E+00 |
| Dr.25277 | Dr.25277 | NM_001012481 | zgc:112187 | **3.01** | 9.24E-24 |
| Dr.41413 |  | NM_001045403 | zgc:153694 | **3.02** | 6.31E-44 |
| Dr.78107 | Dr.3983 | NM_200900 | zgc:77831 | **3.02** | 2.56E-07 |
| Dr.79196 | Dr.34523 | NM_001008628 | zgc:101729 | **3.02** | 2.71E-14 |
| Dr.81582 |  | NM_001045250 | zgc:136983 | **3.03** | 1.97E-06 |
| Dr.45535 |  | NM_001020755 | zgc:110708 | **3.03** | 0.00E+00 |
| Dr.3854 | Dr.3854 | NM_199777 | sec23b | **3.05** | 1.01E-27 |
| Dr.24876 | Dr.24876 | CN177044 | LOC563686: Similar to rhamnose binding lectin STL2 | **3.06** | 0.00E+00 |
| Dr.18507 |  | NM_200750 | zgc:73211 | **3.07** | 0.00E+00 |
| Dr.84509 | Dr.16614 | NM_001007347 | zgc:103481 | **3.07** | 1.30E-36 |
| Dr.83242 | Dr.20655 | NM_200484 | zgc:63691 | **3.07** | 3.21E-29 |
| Dr.90252 |  | NM_001025184 | zgc:114162 | **3.08** | 3.75E-15 |
| Dr.77980 | Dr.11519 | NM_213205 | m6pr | **3.08** | 0.00E+00 |
| Dr.10520 | Dr.10520 | NM_153670 | trh1 | **3.09** | 1.38E-08 |
| Dr.76033 | Dr.898 | NM_213387 | pgk1 | **3.10** | 0.00E+00 |
| Dr.79318 | Dr.30177 | NM_212806 | cyp17a1 | **3.10** | 0.00E+00 |
| Dr.77581 | Dr.3684 | NM_212636 | zgc:77542 | **3.12** | 0.00E+00 |
| Dr.85164 | Dr.15495 | BC090689 | bmp6 | **3.12** | 6.65E-24 |
| Dr.80585 | Dr.42837 | BQ260954 | LOC553480 | **3.14** | 0.00E+00 |
| Dr.75077 | Dr.12 | Z32814 | pdgfra | **3.15** | 2.19E-10 |
| Dr.12383 | Dr.12383 | NM_131798 | bcmo1 | **3.16** | 0.00E+00 |
| Dr.81204 | Dr.12660 | NM_001004600 | zgc:92077 | **3.16** | 0.00E+00 |
| Dr.90953 |  | CK679267 | im:7140613 | **3.19** | 7.65E-24 |
| Dr.80397 |  | NM_001044994 | LOC562734 | **3.19** | 1.21E-28 |
| Dr.79928 | Dr.7392 | NM_199738 | scrn3 | **3.20** | 0.00E+00 |
| Dr.76052 |  | NM_001040349 | zgc:136953 | **3.21** | 1.47E-16 |
| Dr.86246 | Dr.18494 | NM_201100 | zgc:56080 | **3.22** | 0.00E+00 |
| Dr.81683 | Dr.20582 | NM_200259 | rbpms2 | **3.23** | 7.15E-34 |
| Dr.24158 | Dr.2999 | NM_199772 | zgc:66414 | **3.23** | 0.00E+00 |
| Dr.116279 |  | TC281309 | LOC795928: Hypothetical protein LOC795928 | **3.24** | 0.00E+00 |
| Dr.83418 |  | NM_001006101 | zgc:101596 | **3.24** | 5.65E-08 |
| Dr.84014 | Dr.16177 | AL717932 | LOC555286: Hypothetical LOC555286 | **3.26** | 3.01E-22 |
| - | Dr.37402 | CK677752 | im:7138714 | **3.26** | 2.18E-32 |
| Dr.77004 | Dr.12498 | NM_200898 | rad1 | **3.28** | 0.00E+00 |
| Dr.85416 | Dr.32084 | NM_001002558 | zgc:92767 | **3.29** | 0.00E+00 |
| Dr.77901 |  | NM_001083557.1 | MGC163094: Hypothetical LOC565897 | **3.29** | 3.04E-30 |
| Dr.76975 | Dr.18395 | BC045503 | wu:fb55g02 | **3.30** | 5.40E-06 |
| Dr.11184 | Dr.11184 | NM_200723 | zgc:73126 | **3.30** | 3.16E-40 |
| Dr.13965 | Dr.13965 | AI396796 | wu:fb14b11 | **3.30** | 3.55E-12 |
| Dr.14731 |  | NM_001037229 | zgc:122989 | **3.31** | 1.17E-07 |
| Dr.77838 | Dr.14802 | BC091465 | zgc:110553 | **3.31** | 7.45E-24 |
| Dr.84666 | Dr.16139 | NM_205724 | zgc:100907 | **3.31** | 8.29E-22 |
| Dr.4573 |  | AL954673 | lgals9l1 | **3.33** | 0.00E+00 |
| Dr.90580 | Dr.29325 | CK362559 | Transcribed locus | **3.35** | 0.00E+00 |
| Dr.80619 | Dr.12670 | NM_200511 | zgc:66242 | **3.35** | 7.52E-24 |
| Dr.17618 |  | NM_001005981 | kng1 | **3.36** | 2.47E-30 |
| Dr.81450 |  | NM_001030259 | zgc:110290 | **3.37** | 1.12E-44 |
| Dr.78310 | Dr.3133 | NM_213097 | pla2g6 | **3.38** | 3.75E-23 |
| Dr.27055 |  | AL922223 | map3k5 | **3.39** | 7.40E-40 |
| Dr.75918 |  | AA605937 | wu:fa25h05 | **3.39** | 1.41E-33 |
| unknown | Dr.37237 | CK688336 | im:7151384 | **3.40** | 2.36E-11 |
| Dr.80868 | Dr.8125 | NM_131340 | thrb | **3.42** | 1.75E-19 |
| Dr.83505 | Dr.20665 | NM_200299 | bbs5 | **3.43** | 5.84E-28 |
| Dr.82419 | Dr.26468 | NM_200354 | zgc:63972 | **3.45** | 6.15E-38 |
| Dr.83505 |  | TC293626 | bbs5: Bardet-Biedl syndrome 5 | **3.47** | 2.85E-18 |
| Dr.38226 |  | BC093325 | fbxo16 | **3.47** | 1.19E-10 |
| Dr.123292 |  | BM037355 | Transcribed locus | **3.51** | 0.00E+00 |
| Dr.29076 |  | NM_213247 | zgc:77651 | **3.52** | 3.34E-09 |
| Dr.78523 | Dr.10320 | NM_199212 | sgk | **3.52** | 0.00E+00 |
| Dr.88109 | Dr.37715 | CN013847 | im:7138535 | **3.52** | 4.04E-38 |
| Dr.24816 | Dr.24816 | NM_199623 | zgc:56703 | **3.54** | 0.00E+00 |
| Dr.39199 |  | CK684313 | wu:fa92c07 | **3.54** | 0.00E+00 |
| Dr.47027 | Dr.51160 | NM_199988 | zgc:73218 | **3.54** | 6.16E-42 |
| Dr.92843 | Dr.25529 | BC078428 | phex | **3.54** | 3.96E-31 |
| Dr.11428 | Dr.11428 | BC055667 | svil | **3.54** | 0.00E+00 |
| Dr.80157 | Dr.7149 | NM_198807 | zgc:56161 | **3.57** | 0.00E+00 |
| Dr.84511 |  | NM_001024416 | zgc:112399 | **3.58** | 1.49E-41 |
| Dr.88360 |  | CN022795 | LOC796385: Similar to interferon-inducible protein Gig2 | **3.58** | 3.43E-15 |
| Dr.83806 |  | NM_001002330 | zgc:92419 | **3.61** | 1.47E-40 |
| Dr.77980 |  | NM_213205 | m6pr | **3.62** | 7.73E-19 |
| Dr.89197 | Dr.37343 | CK679336 | im:7140695 | **3.63** | 4.92E-14 |
| Dr.597 | Dr.597 | NM_131408 | vegfa | **3.65** | 3.72E-06 |
| Dr.26907 | Dr.26907 | NM_212795 | dnd | **3.66** | 0.00E+00 |
| Dr.81293 | Dr.19643 | NM_001005292 | fabgl | **3.68** | 0.00E+00 |
| Dr.78272 | Dr.7102 | CN505775 | c20orf149l | **3.69** | 0.00E+00 |
| Dr.88218 | Dr.30411 | NM_214785 | siat6l | **3.69** | 1.13E-14 |
| Dr.11569 | Dr.11569 | NM_199872 | osbpl2 | **3.76** | 0.00E+00 |
| - |  | XM_690597 | A2CET2_BRARE | **3.83** | 1.09E-06 |
| Dr.79907 | Dr.6259 | NM_201479 | nucb2b | **3.85** | 0.00E+00 |
| Dr.77957 |  | AI626450 | wu:fc06d01 | **3.85** | 0.00E+00 |
| Dr.76743 | Dr.14834 | NM_001003551 | zgc:101136 | **3.86** | 0.00E+00 |
| Dr.81582 |  | NM_001045250 | zgc:136983 | **3.89** | 0.00E+00 |
| Dr.75676 |  | NM_001077173 | zgc:153017 | **3.91** | 0.00E+00 |
| Dr.90759 | Dr.29559 | CK398479 | Transcribed locus | **3.92** | 2.89E-34 |
| Dr.81219 | Dr.39454 | NM_001004631 | zgc:101731 | **3.95** | 1.58E-18 |
| Dr.80447 | Dr.19450 | NM_199833 | zgc:55983 | **3.96** | 0.00E+00 |
| Dr.84768 |  | NM_001040316 | zgc:136753 | **3.96** | 0.00E+00 |
| Dr.84963 | Dr.24649 | NM_200097 | brms1 | **3.98** | 0.00E+00 |
| Dr.23544 | Dr.194 | NM_131339 | rarg | **3.99** | 0.00E+00 |
| Dr.78338 | Dr.21412 | BC056715 | txndc1 | **3.99** | 1.68E-31 |
| Dr.82520 | Dr.27052 | NM_194391 | hdr | **4.01** | 2.32E-26 |
| Dr.35585 |  | AI667197 | si:ch211-117n7.2 | **4.01** | 0.00E+00 |
| Dr.80485 |  | AW175095 | wu:fi32b12 | **4.03** | 0.00E+00 |
| Dr.2860 |  | NM_131401 | hspa8 | **4.04** | 0.00E+00 |
| Dr.79234 | Dr.11523 | NM_212694 | mkln1 | **4.05** | 0.00E+00 |
| Dr.75780 | Dr.555 | NM_131041 | neurog1 | **4.12** | 1.87E-14 |
| Dr.80683 | Dr.37680 | AW174144 | gpr137ba | **4.13** | 0.00E+00 |
| Dr.82956 | Dr.36442 | NM_001004646 | zgc:101089 | **4.13** | 4.44E-16 |
| Dr.85868 | Dr.18358 | AL921964 | zgc:152977 | **4.16** | 0.00E+00 |
| Dr.88939 | Dr.25921 | NM_001006092 | zgc:101623 | **4.17** | 0.00E+00 |
| Dr.133248 |  | XP_694226 | LOC565872 hypothetical LOC565872 | **4.17** | 4.45E-15 |
| Dr.83494 | Dr.14518 | BC090293 | zgc:110788 | **4.19** | 0.00E+00 |
| Dr.79234 | Dr.11523 | NM_212694 | mkln1 | **4.25** | 0.00E+00 |
| Dr.77586 |  | CF924885 | chac1 | **4.26** | 0.00E+00 |
| Dr.75731 | Dr.12259 | BC053242 | btg4 | **4.27** | 1.25E-08 |
| Dr.21037 | Dr.21037 | NM_201148 | zgc:55310 | **4.27** | 0.00E+00 |
| Dr.25529 |  | BC078428 | phex | **4.29** | 8.30E-40 |
| Dr.84956 | Dr.20787 | NM_001007769 | zgc:103549 | **4.30** | 8.68E-30 |
| Dr.81706 |  | CK687693 | im:7150667 | **4.32** | 6.72E-27 |
| Dr.85820 | Dr.17145 | NM_194405 | kctd12.1 | **4.34** | 8.53E-23 |
| Dr.79285 |  | NM_199783 | klhl11 | **4.37** | 1.07E-17 |
| Dr.83410 | Dr.14374 | NM_001002216 | gadd45a | **4.38** | 0.00E+00 |
| Dr.84741 | Dr.20896 | CA496350 | zgc:113085 | **4.41** | 0.00E+00 |
| Dr.78678 | Dr.20120 | NM_001002596 | zgc:92313 | **4.42** | 1.36E-09 |
| Dr.84324 |  | BI864048 | LOC555852: Similar to MGC107856 protein | **4.52** | 0.00E+00 |
| Dr.108245 | Dr.37326 | CK679963 | zgc:113169 | **4.52** | 2.06E-32 |
| Dr.82131 | Dr.10074 | NM_194426 | pdgfa | **4.55** | 6.22E-14 |
| Dr.78732 | Dr.2661 | NM_001003643 | zgc:100787 | **4.61** | 0.00E+00 |
| Dr.15898 | Dr.15898 | CN015332 | LOC560708: Hypothetical LOC560708 | **4.64** | 0.00E+00 |
| Dr.36523 | Dr.36339 | NM_001004579 | zgc:92316 | **4.70** | 0.00E+00 |
| Dr.81219 | Dr.39454 | NM_001004631 | zgc:101731 | **4.73** | 9.22E-16 |
| Dr.39844 |  | AW344111 | LOC798470: hepatocell.carcinoma-ass. antigen 137 | **4.73** | 3.13E-29 |
| Dr.78284 |  | BC095108 | zgc:109987 | **4.85** | 1.03E-33 |
| Dr.108112 |  | TC295486 | LOC559239: Hypothetical LOC559239 | **4.86** | 8.55E-13 |
| Dr.75663 | Dr.26975 | NM_180964 | cldnd | **4.86** | 2.65E-41 |
| Dr.78678 | Dr.20120 | NM_001002596 | zgc:92313 | **4.87** | 0.00E+00 |
| Dr.7378 | Dr.7378 | CN178098 | wu:fj19h04 | **4.89** | 0.00E+00 |
| Dr.118319 | Dr.15825 | CO351613 | LOC563525: Hypothetical LOC563525 | **4.90** | 7.65E-30 |
| Dr.84881 | - | NM_001017616 | lypla1 | **4.91** | 3.12E-35 |
| Dr.1214 | Dr.1214 | NM_201112 | arl6ip | **5.05** | 1.24E-18 |
| Dr.77332 |  | TC291739 | atp6v0d1: ATPase, H+ transporting, V0 subunit D isoform 1 | **5.13** | 3.38E-26 |
| Dr.90156 |  | Q567C9 | zgc:112151 | **5.14** | 0.00E+00 |
| Dr.84495 | Dr.13710 | NM_001002637 | zgc:92204 | **5.14** | 0.00E+00 |
| Dr.76517 | Dr.2788 | BC067693 | si:dkeyp-86b9.2 | **5.17** | 3.31E-11 |
| Dr.81185 |  | NM_200151 | josd2 | **5.44** | 0.00E+00 |
| Dr.53289 |  | BI885245 | LOC561189: Similar to zygote arrest 1 | **5.54** | 0.00E+00 |
| Dr.81507 | Dr.11481 | NM_001002352 | zgc:92153 | **5.57** | 9.19E-27 |
| Dr.79522 | Dr.7805 | NM_199963 | zgc:55665 | **5.70** | 0.00E+00 |
| Dr.84834 | Dr.14909 | NM_001007349 | zgc:103473 | **5.89** | 4.78E-41 |
| Dr.81650 | Dr.18506 | NM_200891 | zgc:77880 | **5.90** | 0.00E+00 |
| Dr.3433 | Dr.3433 | NM_200189 | dia1 | **5.96** | 9.70E-42 |
| Dr.76805 | Dr.13970 | NM_200860 | arl4a | **6.08** | 1.40E-45 |
| Dr.6402 | Dr.6402 | CN841377 | LOC558422: Hypothetical LOC558422 | **6.09** | 0.00E+00 |
| Dr.77837 | Dr.6823 | NM_213316 | zgc:77004 | **6.22** | 0.00E+00 |
| Dr.77576 | Dr.2071 | NM_212726 | zgc:56011 | **6.36** | 0.00E+00 |
| Dr.428 | Dr.428 | NM_213439 | mid1ip1 | **6.61** | 0.00E+00 |
| Dr.79350 | Dr.7775 | NM_213289 | zgc:85807 | **6.61** | 0.00E+00 |
| Dr.428 | Dr.428 | NM_213439 | mid1ip1 | **6.62** | 0.00E+00 |
| Dr.82489 |  | TC297904 | LOC555395: Hypothetical LOC555395 | **7.10** | 3.15E-41 |
| Dr.80630 | Dr.7313 | NM_199743 | zgc:73340 | **7.13** | 0.00E+00 |
| Dr.86329 |  | BM776945 | pld1 | **7.15** | 0.00E+00 |
| Dr.4206 | Dr.4206 | NM_214757 | zgc:76977 | **7.37** | 0.00E+00 |
| Dr.79024 | Dr.13092 | NM_200123 | zgc:63565 | **7.40** | 0.00E+00 |
| Dr.79757 | Dr.6604 | NM_213180 | zgc:77112 | **8.06** | 0.00E+00 |
| Dr.75731 | Dr.12259 | NM_198121 | btg4 | **8.10** | 0.00E+00 |
| Dr.81135 |  | AW282069 | zgc:153424 | **8.70** | 0.00E+00 |
| Dr.104504 |  | AI384285 | wu:fb07a10 | **8.75** | 0.00E+00 |
| Dr.16053 |  | BM531631.1 | LOC797938: Hypothetical protein LOC797938 | **9.17** | 0.00E+00 |
| Dr.83207 | Dr.12352 | NM_200187 | paqr5a | **9.33** | 1.06E-34 |
| Dr.75731 | Dr.12259 | BC053242 | btg4 | **9.54** | 0.00E+00 |
| Dr.83474 | Dr.14499 | NM_200329 | zgc:56701 | **9.61** | 0.00E+00 |
| Dr.79394 | Dr.7938 | NM_201215 | zgc:56248 | **10.28** | 1.40E-45 |
| Dr.12572 | Dr.12572 | NM_205580 | mos | **10.35** | 1.04E-36 |
| Dr.134770 | Dr.29514 | CK397321 | Transcribed locus | **12.49** | 5.41E-14 |

**Bold** = >2-fold change
