# Supplementary material for: ERK1 and ERK2 MAPK are key regulators of distinct gene sets in zebrafish embryogenesis
Source: BMC Genomics. 2008 Apr 28;9:196. doi: 10.1186/1471-2164-9-196 (PMC2390552; doi:10.1186/1471-2164-9-196)
Supplement: Additional file 2 — ERK1 knockdown phenotype at 24 and 48hpf. Images show representative examples of surviving ERK1 morpholino injected embryos with a tailless phenotype at 24 and 48hpf. [file 1471-2164-9-196-S2.pdf]

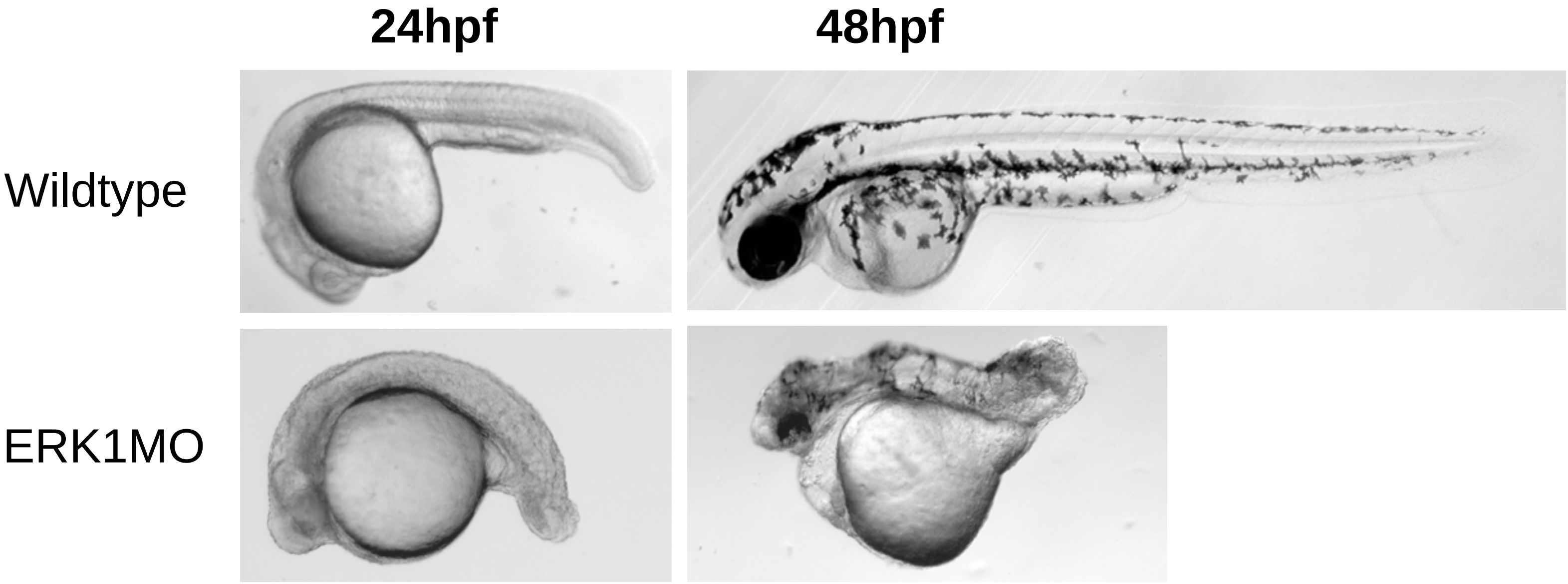

Supplemental figure: surviving ERK1 morpholino injected embryos, compared to wildtype embryos at 24 and 48hpf.
